# Double strand breaks drive toxicity in a Huntington’s disease mouse model with or without somatic expansion

**DOI:** 10.1038/s41467-026-72382-z

**Published:** 2026-05-06

**Authors:** Aris A. Polyzos, Ana Cheong, Jung Hyun Yoo, Lana Blagec, Zachary D. Nagel, Cynthia T. McMurray

**Affiliations:** 1 Division of Molecular Biophysics and Integrated Bioimaging, Lawrence Berkeley National Laboratory, Berkeley, CA USA; 2 Department of Environmental Health, John B Little Centre of Radiation Sciences, Harvard T.H. Chan School of Public Health, Boston, MA USA

**Keywords:** Molecular biology, Huntington's disease

## Abstract

Genome-wide association studies (GWAS) have provided strong evidence that modifiers of CAG tract length have a crucial influence on Huntington disease onset, but somatic expansion alone may not be sufficient to drive neuronal death. Here, we report that DSBs drive neuropathology in male *HdhQ(150/150*) mice, regardless of somatic expansion of the inherited disease allele. DSBs and somatic expansion occur simultaneously in the HD brain, but the two types of DNA damage drive disease by distinct mechanisms. The site-specific increases in CAG tract length are driven by active mismatch repair (MMR), while DSBs occur genome-wide and are driven by mutant huntingtin-mediated suppression of nonhomologous joining of DNA broken ends. DSBs and transcriptional dysfunction occur in animals that cannot somatically expand their inherited allele. Conversely, suppression of DSBs is sufficient to reverse neuropathology even when somatic expansion is active. We propose that CAG expansion and DSBs promote downstream neuronal pathology as separable drivers. The disease-length CAG tract leads to early inhibition of DSBR and accumulating DSBs over time ultimately kill neurons.

## Abstract (web summary)

The CAG tract length influences Huntington’s disease onset but alone may not be sufficient to drive neuronal death. Here, the authors report that double strand breaks induce neuronal death, regardless of somatic expansion of the inherited disease allele.

## Introduction

Huntington disease (HD) is one of a class of neurodegenerative diseases caused by an unstable expanded (>35) CAG repeat that underlies disease progression and severity^1,2^. HD is fatal. The pathophysiology of HD is characterized by a slow and steady loss of neurons, leading to a gradual deterioration of motor and cognitive abilities over time^1–4^. Despite years of effort, there is no cure^2^. Small molecule approaches to mitigate the clinical features of HD have been extensive^2,5–8^. While treatments that suppress chorea have had some clinical success^9^, they have had limited impact on neuropathology^5,6,10^. The CAG tract in the mutant protein codes for a long polyglutamine region in the expressed gene product^1–4^. Thus, it has made intuitive sense that lowering the level of the mutant huntingtin protein (mhtt) would be a good approach to offset disease^11,12^. Indeed, injection of a gene silencing miRNA, AMT-130, slows striatal death in a small study of HD patients (https://en.hdbuzz.net/the-first-domino-falls-amt-130-gene-therapy-slows-huntingtons-in-landmark-trials). In general, however, gene-silencing, including interference (RNAi) and microRNA strategies^13–15^, antisense oligonucleotides (ASOs) systems^16–20^, and Clustered Regularly Interspaced Short Palindromic Repeats/Caspase 9 (CRISPR/Cas9) systems^21,22^ have shown promise in model organisms but have not yet translated into successful clinical interventions^23–26^.

The strong inverse relationship between the age of motor onset and CAG repeat length has pointed to the expansion itself as the primary determinant of disease^27–30^. Longer CAG tracts are associated with earlier onset and a more severe phenotype. Some HD patient alleles contain CAG-to-CAA interruptions that reduce the uninterrupted CAG tract without altering the polyglutamine sequence; loss of these interruptions correlates with earlier disease onset^29–37^. These findings indicate that pure CAG length in the DNA, rather than polyglutamine length encoded in the protein product, is the most reliable predictor of age at onset—a relationship confirmed in mouse models^36–39^.

Given the importance of somatic tract length, shortening it by gene editing has been the focus of significant therapeutic efforts^40–42^. Tested approaches to shorten tract length include targeting the CAG repeat directly or by inhibiting DNA repair machinery that strongly modifies somatic expansion^29,30,32,37,43,44^. Genome-wide association studies (GWAS) have identified many key modifiers of onset in humans, including mismatch repair (MMR) proteins^43–47^ and the Fanconi Anemia nuclease FAN1^43,46^. Indeed, disease-accelerating loci in HD patients include *MLH1* (MutL homolog 1), *PMS1* (post meiotic segregation increased 1 homolog), MutS homologs *MSH2* and *MSH3*, which dimerize to form MutSβ), *PMS2* (post meiotic segregation increased 2) and *LIG1* (DNA Ligase 1)^32,43–46^. The remarkable convergence of the GWAS on the MMR components has validated years of study corroborating a role for them in promoting somatic expansion in mouse and cell models for disease^47–50^. Reducing somatic expansion, particularly via FAN1, has been linked to delayed clinical onset^51–53^. CRISPR-Cas9 editing in Huntington’s disease *Htt*^Q111^ mice has further identified repair genes that modify CAG tract length and influence disease progression^48^. Functional gene knockouts have been generated by delivery of adeno-associated virus expressing single guide RNAs to targeted genes of interest in Cas9-expressing *Htt*^Q111^ knock-in mice^48^. The CRISPR screen validated a strong correlation among repair genes that increase or decrease CAG tract length with acceleration or delay of clinical onset^48^, respectively. Loss of MSH3 or FAN1 displays similar effects in disease mice expressing expanded CGG^54^ and GAA^55^ repeats, indicating that the impact of DNA repair proteins on tract length is relevant for other expansion-related diseases. Relationships between the MMR pathway proteins and FAN1 are emerging^56–58^.

Strategies to reduce somatic CAG expansion are shaping new approaches to develop urgently needed therapeutics for HD^29,40–42,59^. While an expanded HD allele is necessary for disease, emerging evidence suggests that somatic expansion alone may not be sufficient to drive neuronal toxicity. For example, somatic expansion occurs throughout life in HD patients, but pathology is not obvious for decades^27,60^. CAG expansions are prominent in medium spiny neurons of the striatum (STR) and in cholinergic interneurons and cerebellar Purkinje cells (PC), but only striatal projection neurons are lost^61^. Thus, expansion alone does not fully account for neurotoxicity. A two-phase pathogenic process is possible where somatic expansion first increases CAG tract length to a level sufficient to alter cellular function, followed by neuronal damage. Indeed, by RNA Seq and modeling analysis, striatal projection neurons in HD patients become vulnerable to death only after expansion reaches a somatic threshold of 150–180 CAGs^28,60^. In mice, a threshold-length huntingtin allele containing 175 CAG repeats^62^ undergoes somatic expansion and mice develop early transcriptional dysfunction as early pathology^63^. Early transcriptional changes in susceptible neurons may contribute to subsequent degeneration. However, loss of MSH3 in *zQ175/MSH3(-/-)* crosses attenuates somatic expansion but does not rescue transcriptional abnormalities^28,63^. This implies that additional DNA-dependent processes may drive neuronal damage and may be influenced by DNA repair or other modifiers.

We have investigated expression and activity of five major DNA repair pathways for their ability to drive neuronal damage in male *HdhQ(150/150*) mice. We report here that error-prone repair of DSBs drives HD neurotoxicity in these animals by mechanisms that are independent of somatic expansion. The disease-length CAG tracts lead to early inhibition of DSBR and the accumulating DSBs, over time, ultimately kill neurons. Although they coexist, CAG expansion and DSBs drive HD neuronal toxicity by distinct mechanisms and are likely to be independent therapeutic targets.

## Results

### CAG expansion is progressive in the brains of *HdhQ(150/150*) mice

**Fig. 1:**
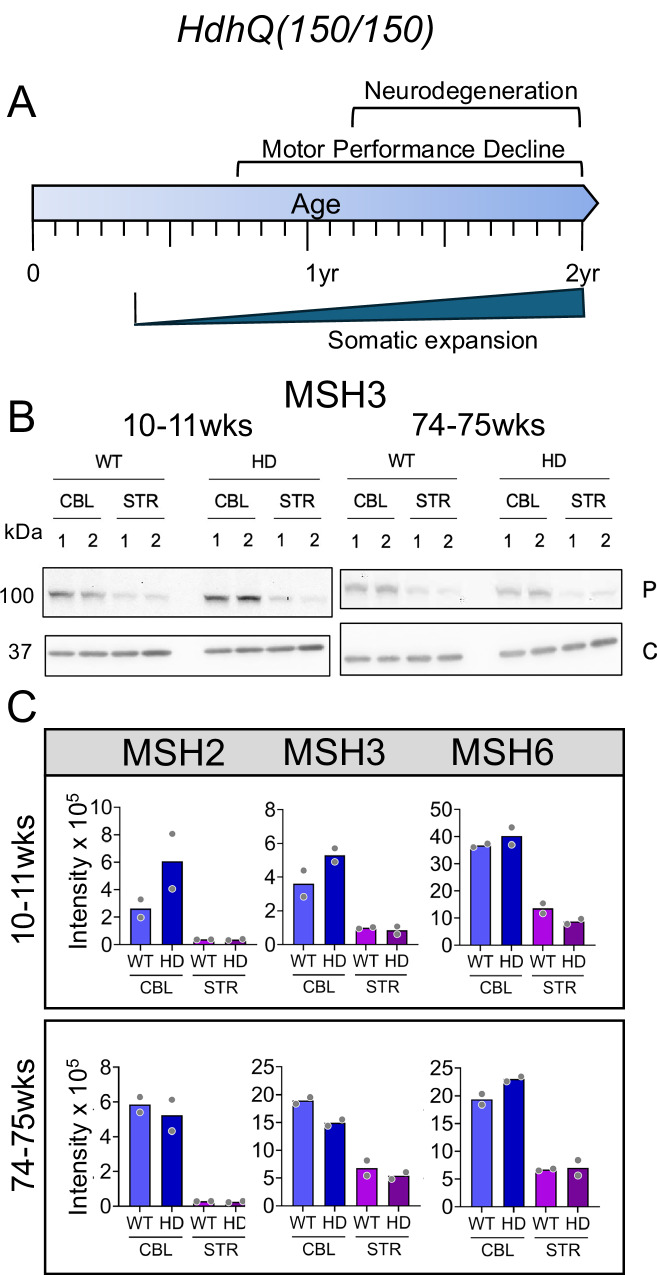
*HdhQ(150/150)* and WT mice express the MMR recognition machinery. **A** Schematic diagram of timing and properties of pathophysiology and somatic expansion in *HdhQ(150/150)*(HD) animals over 2 years, as summarized^7^. **B** Western analysis of MSH3 in brain extracts from the sensitive striatum (STR) and the resistant CBL of genetically identical congenic WT or *HdhQ(150/150*) animals, *n* = 2 at 10–11 weeks and *n* = 2 at 74–75 weeks. The brain extracts of each sample (indicated by numbers 1 and 2) at each age were resolved side by side by SDS-PAGE, transferred to membranes and probed with a specific antibody to one of the mismatch repair proteins (P), either MSH2, MSH3 or MSH6, or to GAPDH (**C**). Molecular weight markers (kD) are shown on the left side of each gel. Gels are shown for MSH3 alone (in B) or for MSH2, MSH3 and MSH6 together (in Supplemental Fig. 2A). **C** The average antibody band IF intensities were quantified and plotted in (**C**) (and in Supplemental Fig. 2) in the STR (purple) and CBL (blue) of WT or *HdhQ(150/150*) (HD) animals at the indicated ages. The bar indicates the average intensity of the points, which are shown as circles. Raw data and uncropped gels are provided as a Source File, and protein primary antibodies are listed in Supplementary Table 1. MSH3 and MSH6 expression was quantified and validated in additional animals at the single cell level in Fig. 2.

We have extensively evaluated and previously published the results of toxicity studies in *HdhQ(150/150*) mice^7,31,64,65^. The *HdhQ150KI* line harbors a long CAG repeat tract of roughly 150 “knocked into” the endogenous mouse gene^66^. Motor abnormalities are detected around 20 weeks (wks)^7,66^; neuronal death in the striatum (STR) was detected around 60 wks^7^, while pathology was spared in the cerebellum (CBL)^7^ (Fig. 1A). The progressive pathology is accompanied by CAG somatic expansion, as previously measured by us and others^33,34^ which begins around 11 wks^31,33,34^, is prominent by 30 wks (Supplemental Fig. 1) and is more extensive in the affected striatum (STR) relative to the resistant CBL^33^. Thus, CAG expansion in *HdhQ(150/150*) animals displays the genotype, age-, and region-specific features of disease toxicity observed in humans and in other mouse strains. To evaluate genetic drivers of neurotoxicity, we generated a congenic strain of *HdhQ(150/150*) mice, which was used in all experiments. The value of a congenic line is that each animal is a genetically identical clone with the same protein expression background, which simplifies the statistical considerations for their experimental use (see “methods”).

MSH3 drives CAG expansion, and, in other lines, mice that express the highest MSH3 level expanded the most^68–70^. MSH2 dimerizes with MSH3 to form MutSβ and with MSH6 to form MutSα^71,72^ but only MutSβ drives somatic expansion of CAG triplet repeats^49^. If expansion were sufficient to drive neuronal toxicity, we expected that MSH3 expression in *HdhQ(150/150*) mice would track with the age-, and region-specific features of disease toxicity. We therefore tested whether MSH3 was present in WT and *HdhQ(150/150*) lines at young (10, 11 wks) or old (74, 75 wks) ages (Fig. 1). Since the *HdhQ(150/150*) colony was genetically identical, we established whether MSH3 was expressed in *HdhQ(150/150*) mice in two sets of age-matched congenic littermates. Brain extracts were resolved by SDS-PAGE, side by side on the same gel (Fig. 1, numbered 1 and 2), transferred to membranes and probed with specific antibodies to MSH3, MSH2, and MSH6 (P) or to GAPDH (C)(Fig. 1B, C; gels shown in Supplemental Fig. 2A). Indeed, the GAPDH signal confirmed that the extracts were loaded in the gel lane at roughly equal amounts (Fig. 1B and Supplemental Fig. 2A), and the antibody signal established that MSH3 (Fig. 1B, C), MSH2, and MSH6 (Supplemental Fig. 2A) were present in the samples (Fig. 1C). MSH3 expression in *HdhQ(150/150*) animals, however, was low in the sensitive STR (Fig. 1B, C)(Supplemental Fig. 2), which expanded the most^33^.

**Fig. 2:**
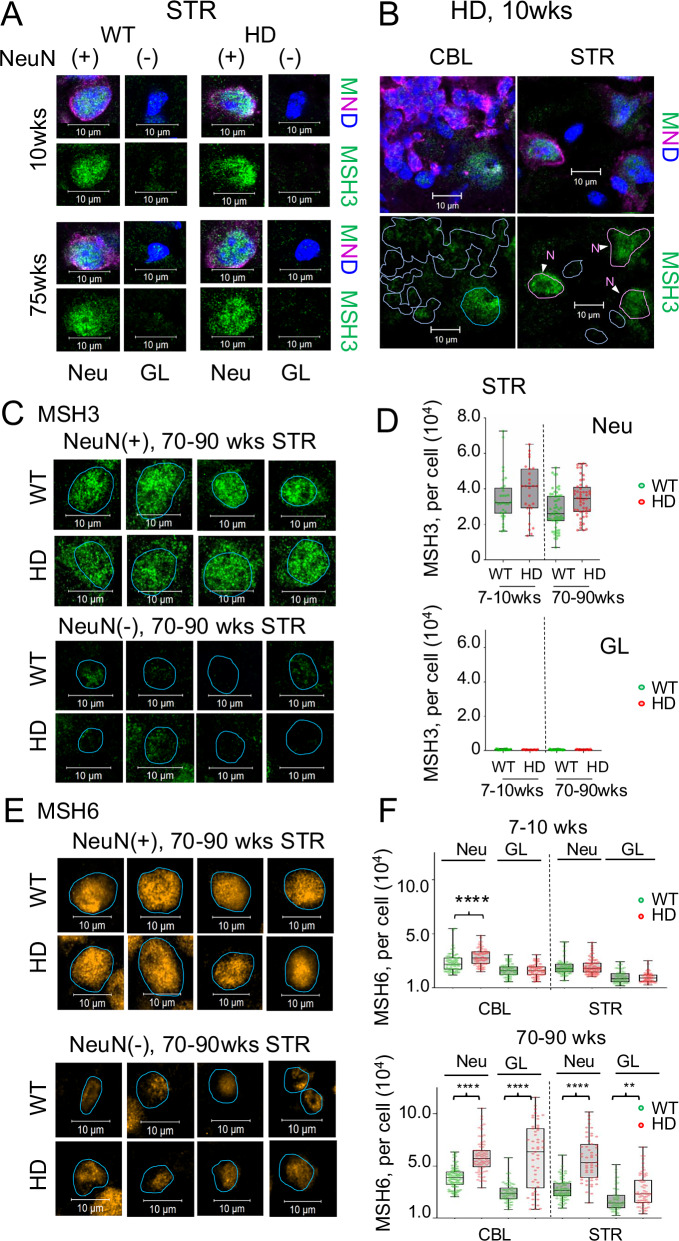
MSH3 expression is abundant in neurons. Immunofluorescence (IF) intensity of MSH3 and MSH6 was assessed in the vulnerable striatum (STR) and in the resistant cerebellum (CBL) of congenic WT and *HdhQ(150/150)* (HD) mice (*n* = 3 per genotype). All IF intensities are expressed in arbitrary units. **A** MSH3 IF intensity in magnified, individual cells from striatal tissue of WT and HD littermates at young (10 wks) and old (75 wks) ages. The MSH3 channel (green) is shown alone (MSH3) or merged (MND) with NeuN (N, magenta) and DAPI (D, blue). Scale bar, 10 µm. **B** Across genotypes, tissue regions and ages, MSH3 expression was ubiquitous, with the highest expression in NeuN(+) neurons (Supplemental Fig. 2B, C). Scale bar is 10 µm. Shown are the STR and CBL in the same tissue section from the 10-week HD mouse in (**A**). In the CBL, the blue outlines highlight ubiquitous MSH3 IF intensity in DAPI-stained cells; in the STR, the purple outlines highlight MSH3 IF intensity in NeuN(+) neurons (N) and blue outlines highlight weak MSH3 IF intensity in NeuN(-) glia. **C**, **E** Representative high-magnification images used for single cell quantification of MSH3 (**D**) or MSH6 (**F**) expression in NeuN(+) neurons (top) and NeuN(–) glia (bottom) from 70 to 90 wks animals. Scale bar, 10 µm. IF intensity for protein was measured with similar results in *n* = 4 random fields. **D** MSH3 was quantified in roughly *n* = 50 neurons (Neu) or glia (GL) in the STR for each genotype and age, 7–10 wks and 70–90 wks. The distribution and median difference was visualized in a box plot, where 50% of the values are in the box, the median intensity is designated by the line, and 25% minimum and 25% maximum values are below and above the box, respectively. **F** Same as (**D**) for MSH6 but in CBL and STR. Statistical differences between WT and HD cells for either protein was determined using one-way ANOVA. There were no significant differences in the median MSH3 intensity. Staining intensity for MSH6 increased with age in HD cells relative to age-matched WT controls in both neurons (****p = 0.0001) and in glia (**p = 0.01).

Somatic expansion is prominent in both neurons and in glia^34,73,74^. Thus, with age, it was possible that MSH3 changed its expression level or its cell type localization in *HdhQ(150/150)* animals in a manner that sensitized striatal neurons to toxicity. To test these possibilities, we quantified the cell type-specific expression of MSH3 and MSH6 from their IF staining intensities at the single cell level in brain tissue sections from additional WT or *HdhQ(150/150)* congenic mice (Fig. 2). Co-staining of protein IF intensity and NeuN(+) neuron-specific marker^75^ was used to quantify neuronal expression, while protein IF intensity in NeuN(-) cells was used to quantify glial expression (Fig. 2A–C). Mismatch repair proteins were ubiquitously expressed in neurons and glia throughout the tissue at both young and old ages (Fig. 2A–C and Supplemental Fig. 2B, C). For example, we probed tissue sections from a littermate pair of WT and *HdhQ(150/150)* at 10 wks, and another littermate pair at 75 wks for MSH3 neuronal and glial localization (Fig. 2A,B). MSH3 was identified by its green emission intensity in magnified images of striatal cells of either genotype (Fig. 2A, MSH3), and, in overlay images, co-stained with DAPI in both NeuN(+) and NeuN(-) cells of the STR (Fig. 2A, M,N,D). However, MSH3 was expressed primarily in neurons (NeuN+) (Fig. 2A), while glial expression (NeuN-) was weak at young (Supplemental Fig. 2B) or at old ages (Fig. 2A). The pattern was reproducible in all examined regions in the tissue slice, for example, in the sensitive STR and the more resistant CBL of the 10wk *HdhQ(150/150)* animal in (A)(Fig. 2B) and at 90 wks (Supplemental Fig. 2). MSH6 showed a similar pattern (Fig. 2E).

The mean expression level and cell-type distribution of MSH3 (Fig. 2C, D) and MSH6 (Fig. 2E, F) were quantified from IF signal intensity at the single-cell level among regions in approximately *n* = 50 randomly selected NeuN(+) neurons or NeuN(–) glia in WT or HD animals aged 7–10 wks or 70–90 wks. The single-cell results were displayed in box-and-whisker plots (Fig. 2D, F), where each point represents a scored cell expressing MSH3 or MSH6. Although neurons expressed more MSH3 than glia in the STR (Fig. 2C), there were no genotype-dependent changes in expression levels with age (Fig. 2D). The cell-type distribution of MSH6 also did not depend on genotype at young ages; however, expression increased with age in *HdhQ(150/150)* mice relative to WT (Fig. 2F). Collectively, these results confirmed that MSH3 was available to drive somatic expansion in neurons of the STR and CBL in *HdhQ(150/150)* mice. However, the level of MSH3 did not fully match the age- and region-specific patterns of expansion or HD toxicity. Neither the cell-type distribution nor the per neuron expression level of MSH3 change with age as expansion and toxicity progressed in these animals. Thus, it was plausible that additional processes, beyond CAG expansion, contributed to selective neuronal damage in male *HdhQ(150/150)* mice and may be influenced by DNA repair or other modifying factors.

### The machinery for common DNA repair pathways is expressed in the affected STR and resistant CBL of WT and *HdhQ(150/150*) mice

**Fig. 3:**
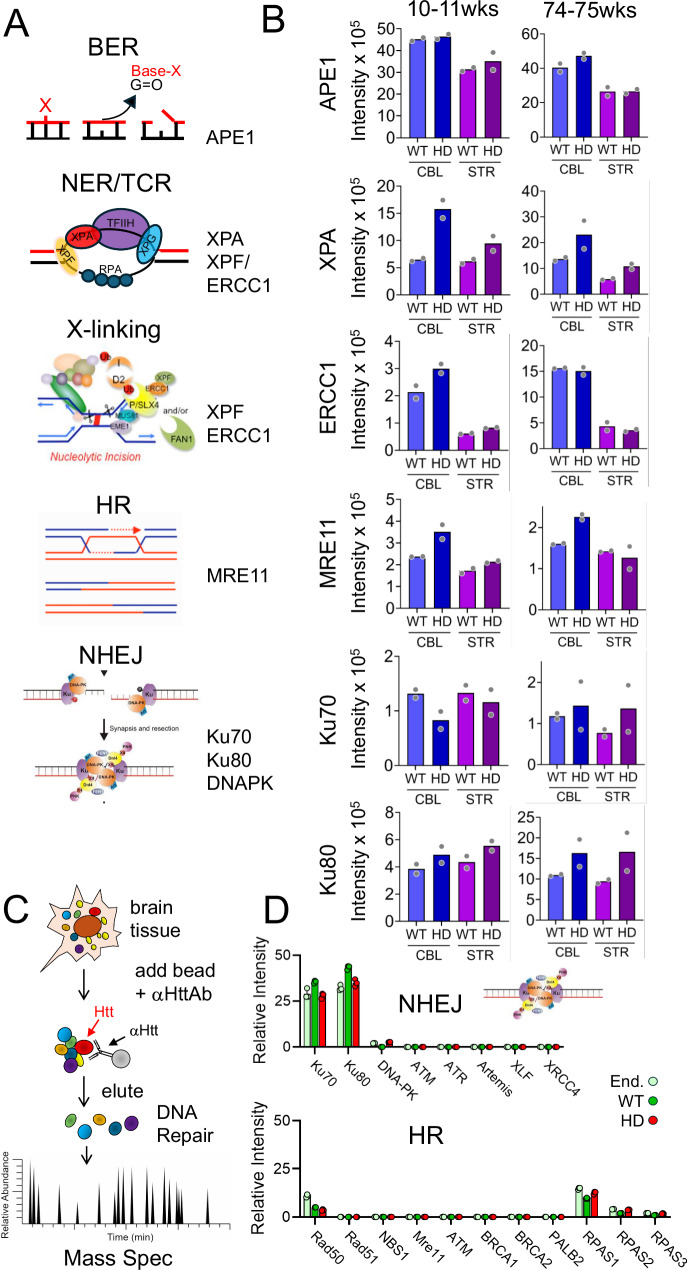
Htt and mhtt interact with the DSBR machinery in primary brain cells from WT and *HdhQ(150/150*) mice. **A** Schematic diagram of major DNA repair pathways^76–78^ for representative pathway proteins shown to the right of the schematic. Specific cartoons for X-linking and NHEJ pathways were taken from Brissett and Doherty^76^. **B** The expression intensity for proteins in (**A**) was determined from Western blot detection as described for MSH3 in Fig. 1B. The protein extracts from congenic animals (*n* = 2 at 10–11 wks and *n* = 2 at 74–75 wks) were resolved side by side on the same SDS-PAGE gel, indicated by the numbers 1 and 2 (gels shown in Supplemental Fig. 3). Bars indicate the average band intensity in the CBL and STR of each genotype and the circles indicate the values for each sample. Raw values and full uncropped gels are provided as Source Files. The protein primary antibodies are listed in Supplemental Table 1. **C**, **D** Immunoprecipitation-mass spectrometry (IP-MS) analysis for interactions of DNA repair proteins with htt or mhtt cells. **C** Schematic diagram of the IP-MS analysis. **D** The interactions between DNA repair proteins and htt were measured in *n* = 3 samples: endogenous NIH3T3 cells (End, light green) or for NIH3T3 cells overexpressing htt (dark green) or mhtt (red). Samples were run in triplicate (*n* = 3), and peak intensities of the capture products were plotted as mean ± standard deviation (Source Data file). The MS capture products from NIH3T3 cells were similar to those captured in striatal tissue extracts from *n* = 3 WT or *HdhQ(150/150*) mice (Supplemental Fig. 4). The Ku70 and Ku80 dimer partners in the NHEJ pathway were the primary capture products, with DNAPK as a minor product. Minor associations were also observed for Rad50 of the HR pathway, and with the general RPA single strand binding proteins. Shown in the inset is a schematic of a classic NHEJ complex taken from Brissett and Doherty^76^.

**Fig. 4:**
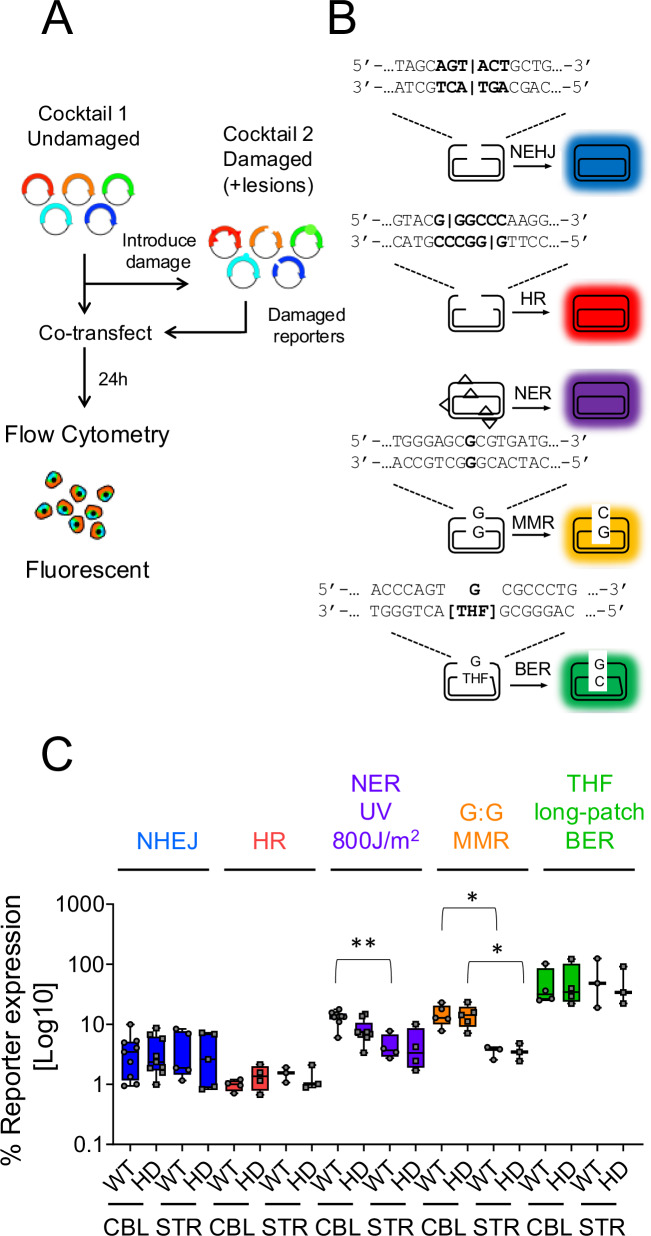
BER, NER/TCR, and MMR activities are not genotype dependent. **A** Schematic for fluorescence multiplex host cell reactivation (FM-HCR) assay. FM-HCR measures DNA repair activity using reporter plasmids, each containing a specific DNA lesion corresponding to one of five major repair pathways^86–88,134^. These lesions disrupt reporter gene expression; restoration of fluorescence indicates successful repair. Two plasmid cocktails are used: Cocktail 1 contains undamaged control reporters, and Cocktail 2 contains lesion-bearing reporters for all five pathways. Both include the same transfection efficiency control and are transfected separately. Repair activity is calculated as % reporter expression by comparing normalized fluorescence from damaged reporters (Cocktail 2) to the fully active undamaged controls (Cocktail 1). Full assay details are provided in the Methods. **B** Color-coded reporter plasmids for five pathways: NHEJ (blue), HR (red), MMR (orange), NER (purple), and BER (green). The lesion locations and nucleotide sequences surrounding each lesion site are shown. For NHEJ and HR reporters, the vertical bar (“|”) indicates the cleavage site that generates the double-strand break (DSB). **C** The primary glial cultures were derived from *n* = 8 embryos per genotype. The data were plotted as % reporter expression on a log10 scale. Similar % reporter activity was obtained in independent replicate transfections; *n* = 3–5 per reporter in the STR or *n* = 4–9 per reporter for CBL. (The exact n for CBL and STR cultures per reporter is provided in the source Data). The distribution and median difference in % expression from the transfections was visualized in a box plot, where 50% of the values are in the box, the median intensity is designated by the line, and 25% minimum and 25% maximum values are below and above the box, respectively. The significance in % reporter activity between WT and HD cells were analyzed using one-way ANOVA. NER and MMR had significantly less activity in the STR relative to the CBL (*p = 0.05), but there was no effect of genotype for any pathway. Full vector schematics are shown in Supplemental Fig. 6A, B.

Mechanisms for DNA repair are well characterized^76–78^. However, crosstalk has long been implicated between mismatch repair (MMR) and the machinery for double strand break repair (DSBR)^79,80^, interstrand cross-link (X-linked pathway)^56–58^, transcription-coupled repair (TCR)/Nucleotide excision repair (TCR/NER)^81,82^, and base excision repair (BER)^83,84^. If a DNA repair pathway other than MMR was impaired or failed to remove DNA damage, then inefficient DNA repair might drive, at least in part, the patterns of toxicity in *HdhQ(150/150*) mice, independently of MSH3-driven somatic expansion. It was also possible that *HdhQ(150/150*) mice lacked components of the repair machinery. Thus, we tested (1) whether the machinery for other major DNA repair pathways was expressed (Figs. 3) and (2) whether these pathways were active (Fig. 4) in the brains of *HdhQ(150/150*) mice and WT mice.

Protein expression was measured for the machinery needed to carry out Homologous Recombination (HR), Non-Homologous End Joining (NHEJ), BER, nucleotide- or transcription coupled excision repair (NER/TCR), and DNA crosslink repair (X-link repair) (Fig. 3A)^76–78^. Due to the complex and multi-component nature of repair complexes, we did not evaluate all proteins in each pathway. Rather, we selected one or two proteins that were representative of pathway function (indicated to the right in Fig. 3A). The protein expression for the excision pathways included the Apurinic/apyrimidinic Endonuclease 1 (APE1) of the BER pathway, Xeroderma Pigmentosum type A protein (XPA), Xeroderma Pigmentosum type F (XPF) and type G (XPG) nucleases for TCR/NER (Fig. 3A). Although the Excision Repair 1 Endonuclease Non-Catalytic Subunit (*ERCC1*) and its binding partner XPF serve as the 5’ core nuclease for bubble excision in NER/TCR, the dimer is also essential during steps of X-link repair (Fig. 3A). Proteins of classic double strand break repair (DSBR) pathways included the meiotic recombination 11 (MRE11) (HR), and Ku70 and Ku80, proteins of NHEJ, which are encoded by the X-ray repair cross-complementing 6 and 5, (*XRCC6)* and (*XRCC5*), respectively.

Brain extracts from the STR and CBL were analyzed at (10, 11 wks) or (74, 75 wks). Six replicate sets of SDS-PAGE gels were transferred to membranes and probed with specific antibodies for one of the representative pathway proteins (P) and GAPDH (C)(Fig. 3B; gels shown in Supplemental Fig. 3). GAPDH confirmed similar loading of samples and antibody staining confirmed that young and old animals of both genotypes expressed the representative proteins from all five major DNA repair pathways (Supplemental Fig. 3)(Fig. 3B). Although not used for plotting, staining for DNA repair proteins was also observed at an intermediate age (19, 20 wks) in a third animal. As judged by antibody staining, toxicity associated with *HdhQ(150/150*) animals did not appear to correlate with deficiencies in the expression level of common DNA repair pathway components (Fig. 3B).

### The normal and mutant huntingtin protein interact with the DSBR machinery

Since brain cells were equally well equipped with the machinery to repair DNA damage, the genotype-specific nature of toxicity raised the possibility that the mutant huntingtin (mhtt) might interact differently with or alter activity of the DNA repair machinery in *HdhQ(150/150*) animals. To determine whether the DNA repair machinery interacted with mhtt and htt, we immunoprecipitated binding partners in brain tissue using a specific htt antibody and identified the pull-down products in WT and *HdhQ(150/150*) animals by mass spectrometry (IP-MS) (Fig. 3C)(Supplemental Fig. 4A). In the brain tissue from *n* = 3 WT and *n* = 3 *HdhQ(150/150*) animals, the htt antibody “pulled down” Ku70 and Ku80, the core dimer for the end joining activity of NHEJ, together with DNAPK as a minor product (Supplemental Fig. 4B)(in source files). Other proteins included the replication protein A proteins (RPA70, RPA32 and RPA14) (Supplemental Fig. 4B), which serve as general single-strand annealing machinery. In brain tissue, the muti-functional DNA repair proteins, Poly {ADP-ribose} polymerase (PARP1) and ligase 3 was “pulled down” by the htt antibody, however, no pathway-specific components of BER, NER/TCR or MMR were otherwise detected in either WT or *HdhQ(150/150*) tissue. These results suggested that huntingtin interactions primarily involve the NHEJ machinery. The interactions were specific since no pulldown products were observed when a non-specific IgG control antibody was substituted in the reaction (Supplemental Fig. 4C). Indeed, Ku70-Ku80 dimer was recovered in the IP reaction whether cells expressed the full-length endogenous huntingtin protein (Supplemental Fig. 4B) or a small, truncated form^85^. To test for weaker huntingtin interactions, we repeated the IP-MS experiment in endogenous NIH3T3 cells (light green) or under conditions in which htt (dark green) or mhtt (red) were overexpressed (Fig. 3D). Cells were transfected with Cytomegalovirus–driven huntingtin cDNA plasmids containing 26 or 51 CAG repeats, respectively. Endogenous Htt in NIH3T3 cells (End) interacted with ligase 3 and PARP1 and weakly associated with RAD50 in the HR pathway (Fig. 3D), regardless of whether these cells overexpressed Htt or mHtt. However, as observed in mouse brain tissue, Ku70-Ku80 and RPA components remained the major pull down products in cells (Supplemental Fig. 4B and Fig. 3D). Although the interaction with the NHEJ machinery was robust in both brain tissue and in cells, the huntingtin interaction was not specific for the mutant form of the protein.

### Activity of BER, NER/TCR, and MMR pathways does not depend on genotype

Since the interaction with the DSBR machinery was not specific to mHTT, we tested whether it might nonetheless alter or impair DSBR activity. To test this possibility, we utilized fluorescent multiplex host cell reactivation (FM-HCR) assays^86–88^ in primary glia from WT and *HdhQ (150/150*) animals (Fig. 4A; Supplemental Fig. 5). Primary glial cultures express the same DNA repair proteins as neurons at lower levels and have served as a robust assay system for DNA repair in brain cells⁸⁷. The FM-HCR assay monitors recovery of fluorescent reporter expression from templates harboring pathway-specific DNA lesions and is evaluated by flow cytometry (Fig. 4A). Since the lesion interferes with the coding sequence and inhibits fluorescence, recovery of signal intensity is a measure of lesion repair (Fig. 4A). Repair activity is reported as % expression, which is the ratio of normalized fluorescence intensity from the damaged reporters (+lesion)(cocktail 2) to the undamaged reporters (no lesions)(cocktail 1) which retain full activity (Fig. 4A). Pathway-specific reporter lesions included an overhang or blunt double strand breaks (DSB) for HR and NHEJ, respectively, a G:G mismatch for MMR, or low ultraviolet (UV) radiation-induced DNA damage (thymidine dimers) for NER (Fig. 4B). BER has two major subpathways for long patch (LP) and short patch (SP) repair, which have overlapping specificity and share many components^89,90^. Thus, we chose LP-BER activity as a representative BER reaction using a tetrahydrofuran (THF) lesion in the reporter plasmid (Fig. 4B)^90^. A detailed published assay^86–88^ is provided in the “Methods” section. The vectors for pMax BFP_scaI and pMAX_mcherry_PspOMI (Supplemental Fig. 6A, B) and GenBank reporters are shown (Source files). A major strength of FM-HCR is that the damaged reporter plasmids for all DNA repair pathways are co-transfected, and their activity is measured simultaneously in the same cell (Fig. 4A). We refer to the collective pathway activity as a “DNA repair landscape”.

**Fig. 5:**
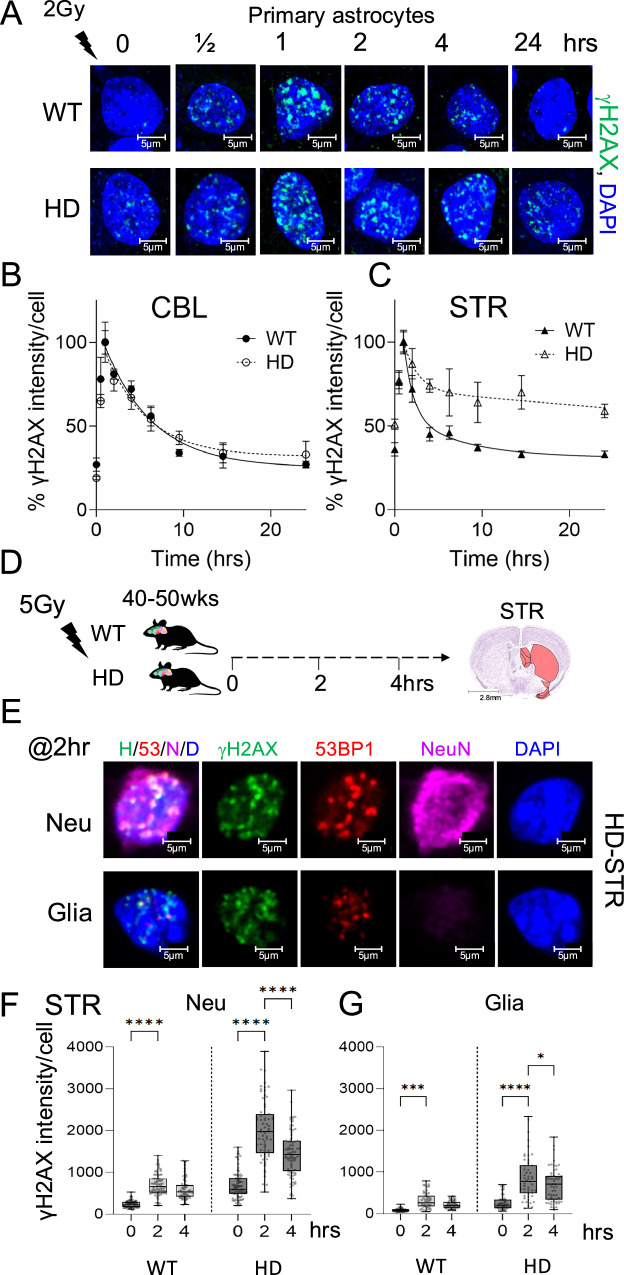
Repair of induced DSBs is inhibited in vitro and in vivo*.* **A** Representative images of γH2AX foci in primary glial cultures from the striatum (STR) of WT (top) and HD (bottom) mice following 2 Gy irradiation. DSBs were detected by γH2AX staining (green) in DAPI-stained nuclei (blue); merged images show foci in turquoise. Scale bar, 5 µm. Formation of γH2AX foci between 0–1 h indicates DSB induction (Supplementary Fig. 8A), whereas loss of foci between 1 and 24 h indicates repair. **B**, **C** Kinetics of γH2AX signal loss in irradiated glial cells derived from the cerebellum (CBL; circles) (**B**) and striatum (STR; triangles) (**C**) of WT (filled symbols, solid lines) and HD (open symbols, dashed lines) mice. Primary cultures were obtained from *n* = 8 embryos per genotype. Mean γH2AX intensity ± S.D. was calculated from *n* = 5 replicate plates per genotype. Intensities were quantified from five regions per plate and from 8–12 striatal or 8–18 cerebellar glial cells per field at each time point post-irradiation. Exact n for counted cells in each field is included in Source File. **D** Schematic of DSB induction in the STR of 40–50-week-old WT and HD mice following a single 5 Gy radiation dose. γH2AX foci were quantified in the STR of *n* = 12 animals per genotype, *n* = 3 at 0, 1, 2, and 4 h time points post-irradiation. The analyzed STR region highlighted in red is taken from the Allen Brain Atlas¹³⁵. **E** Representative IF images of striatal neurons (NeuN(+)) and glia (NeuN(–)) at 2 h post-irradiation. Individual channels: γH2AX (green), 53BP1 (red), NeuN (magenta), and DAPI (blue) or overlay of all four (H/53/N/D). Scale bar, 5 µm. Single-cell quantification of γH2AX intensity in striatal neurons (**F**) and glia (**G**). Cell counts included *n* = 45–88 neurons or *n* = 42–64 glial cells per animal, as listed per animal in source data files. Data are presented as box plots (median line; box = middle 50%; whiskers = quartiles). Statistical significance was assessed using one-way ANOVA (****p = 0.0001; ***p = 0.001; *p = 0.05).

We evaluated the activity of the DSBR machinery, which interacted with mhtt, alongside the lesion-specific activity of BER, NER/TCR and MMR (Fig. 4B)(Supplemental Fig. 5), which largely lacked interactions with htt (Fig. 3D and Supplemental Fig. 4B). In both the STR and the CBL, all DNA repair pathways in the landscapes of WT and *HdhQ (150/150)* animals were active to varying degrees in cultured glial cells (Fig. 4C). BER, NER/TCR, and MMR had robust activity in both WT and HD cells (Fig. 4C). Although there were some regional expression differences (CBL > STR), no significant genotype-specific effects were detected (Fig. 4C). Consistently, the HD/WT ratio of % reporter expression in the CBL and STR was close to 1.0 for all pathways (Supplemental Fig. 5A, B). Thus, the excision repair machinery with few specific htt pull-down products (Fig. 3D and Supplemental Fig. 4B) displayed no measurable deficit in DNA repair activity (Fig. 4C). DSBR activity, however, was low and variable, which precluded statistical distinctions for these pathways using the FM-HCR approach (Fig. 4V and Supplemental Fig. 5B). Therefore, the genotypic differences in DSBR activity were determined in vitro (Fig. 5A–C) and in vivo (Fig. 5D–G) using radiation, where DSBs were induced and repair was followed by the loss of the DSBs post irradiation.

### Induced DSBs *in HdhQ(150/150*) mice are inefficiently repaired in cells

DSBs in primary glial cells were induced in vitro by exposure to ionizing radiation (2 Gy). Low levels of radiation did not kill cells (Supplemental Fig. 7A–C), but elevation of oxidative DNA damage can lead to DSBs^88^ when radiolysis of water generates hydroxyl radicals (OH°)^91^. If HD brain cells had a functional deficit in repairing DSBs, we expected that γH2AX foci would form but, after exposure to radiation, loss of the γH2AX foci in the primary glia would be slower in cells isolated from *HdhQ(150/150*) mice relative to WT cells. Indeed, radiation exposure led to prominent formation of γH2AX foci (Fig. 5A), which occurred to a similar extent and at a similar rate in glia from both WT and *HdhQ(150/150*) mice by 1 hr post-irradiation (Supplemental Fig. 8A). Thus, induction of DSBs did not differ between genotypes. Over the next 23 h, the loss of γH2AX foci was rapid and equivalent in glial cultures from resistant CBL of both WT and *HdhQ(150/150*) cells (Fig. 5B), and in the STR of WT cells (Fig. 5C). However, γH2AX foci level remained elevated in *HdhQ(150/150*) cultures relative to WT cells (Fig. 5C), indicating that DSBR was suppressed and DSBs were inefficiently removed in the disease cells.

### Induced DSBs *in HdhQ(150/150*) mice preferentially form in neurons but are inefficiently repaired in vivo

Inhibition of DSBR also occurred in vivo (Fig. 5D-G). The radiation experiment was repeated in living WT and *HdhQ(150/150*) mice by exposing adult animals (40–50 wks, data in source files) to a higher radiation dose (5 Gy) (Fig. 5D). Animals (*n* = 3) of each genotype were sacrificed immediately after irradiation or at 2 or 4 h post-irradiation to evaluate DSB levels using immunofluorescence (IF) staining intensity of γH2AX (green) or 53BP1 (red) DSB markers (examples shown in Fig. 5E). In magnified tissue sections (Fig. 5E), each antibody or stain is detected 2 h post-irradiation by its emission in its own channel (Fig. 5E, panels 2–4 as marked) or in an overlay of all four stains/antibodies (Fig. 5E, H/y/53/N/D, panel 1). Cell nuclei were visualized with DAPI (blue), DSBs by γH2AX (green) or 53BP1 (red), and cell type by NeuN (pseudo-colored magenta), where NeuN(+) cells are neurons and NeuN(−) cells are glia. Co-staining for γH2AX, 53BP1, and NeuN confirmed the presence of DSBs after irradiation in the STR of HD animals in both NeuN(+) neurons (Fig. 5E, top panels) and NeuN(−) glia (Fig. 5E, bottom panels). Additional images are shown over the entire time course in both tissue (Supplemental Fig. 8B, top panel) and in magnified cells from the tissues (Supplemental Fig. 8B and Supplemental Fig. 9).

The staining patterns were the same in all regions of the tissue sections and in each animal tested. Thus, we quantified DSBs at the single-cell level in the STR by measuring γH2AX signal intensity in *n* = 30–70 NeuN(+) neurons and *n* = 30–70 NeuN(−) glia, randomly sampled among regions at each time point (Fig. 5F, G). Single-cell data from WT or HD tissue at each post-irradiation time were displayed as box-and-whisker plots (WT or HD), where each dot represents one scored γH2AX-positive cell (Fig. 5F, G). By 2 h, DSB levels were significantly higher in the STR of *HdhQ(150/150)* tissue relative to WT in both neurons (Fig. 5F) and glia (Fig. 5G), confirming formation of DSBs in both genotypes. As observed in vitro (Fig. 5A–C), DSB markers preferentially co-stained with NeuN(+) neurons in *HdhQ(150/150)* tissue (Fig. 5E, F), while staining was weaker in NeuN(−) glia (Fig. 5E, G). Thus, in vivo, HD cells—particularly neurons—appeared more susceptible to DNA damage than WT cells.

The efficiency of DSBR was determined from the loss of γH2AX signal intensity staining between 2 and 4 h (Fig. 5F, G). By 4 h, the DSBR activity was apparent by the decline of the γH2AX signal intensity in both genotypes (Fig. 5F, G). However, DSBs remained significantly elevated (more than 2-fold) in both neurons and in glia in the STR of HD animals relative to WT tissue. As judged by the antibody signal, DSBs were inefficiently repaired in disease tissue. This was visually obvious by the enhancement of γH2AX staining intensity in HD neurons relative to WT in overlay IF images, where γH2AX was pseudo-colored red (Supplemental Fig. 9). Although DSBs formed in the brains of WT and HD animals, DSBs in the latter were poorly repaired, particularly in neurons. Collectively, the FM-HCR (Fig. 4), radiation (Fig. 5) and IP-MS (Fig. 3D) analyses suggested that htt/mhtt interactions altered the activity of the DSBR machinery. Htt had few interactions with components of NER, BER and MMR pathways (Fig. 3D), and their activities were not significantly altered by genotype (Fig. 4C and Supplemental Fig. 4B). In contrast, DSBR pathways, whose components interacted with htt, were selectively inhibited in vitro (Fig. 5B, C) and in vivo (Fig. 5D–G). The suppression of DSBR was genotype, cell type- and region-specific, consistent with features of human disease.

### DSBs accumulate in the brains of *HdhQ(150/150*) mice

**Fig. 6:**
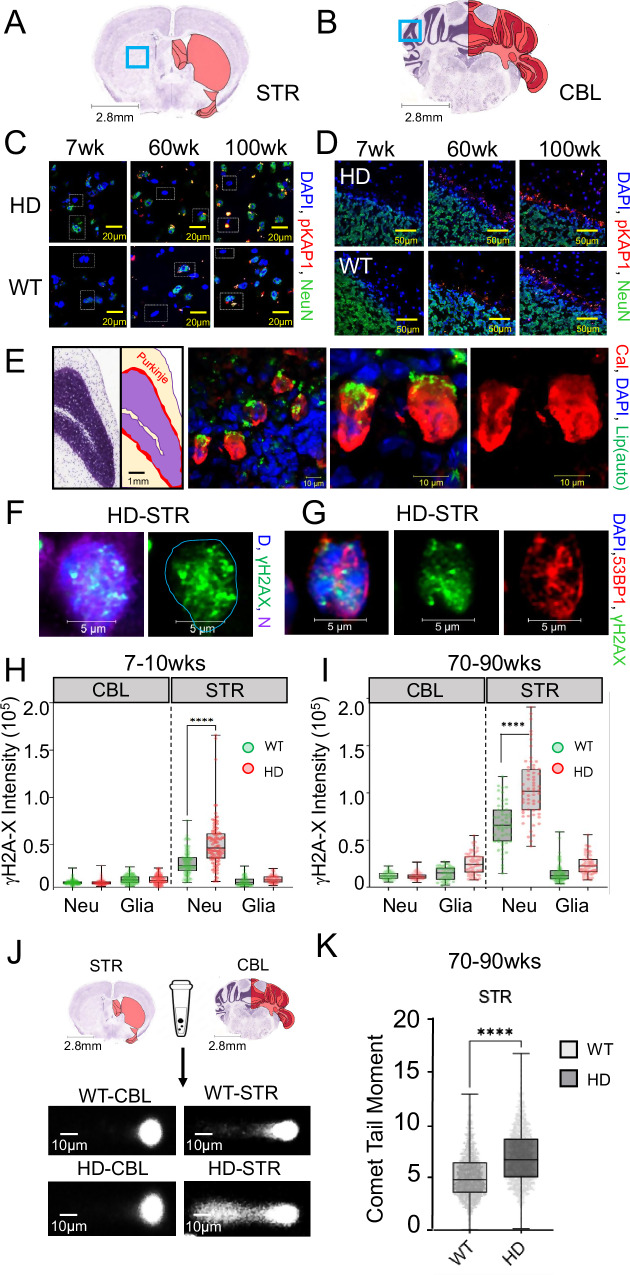
Double-strand breaks (DSBs) accumulate in neurons of HD mice. **A**, **B** Allen Brain Atlas images of the striatum (STR) and cerebellum (CBL) are shown alongside corresponding H&E-stained experimental sections. Scale bar is 1 mm. NeuN staining of the STR is presented at left. All IF intensities are expressed as arbitrary units. Analyzed regions are highlighted in the red and blue boxes. **C** IF images of striatal sections from HD (top) and WT (bottom) male mice at 7, 60, and 100 weeks, stained for pKAP-1 (red), DAPI (blue), and NeuN (green). Increased pKAP-1 staining is observed in HD striatum with age. Scale bar, 20 µm. **D** pKAP-1 staining in the CBL is restricted mainly to a single Purkinje cell (PC) layer adjacent to dense granule neurons. Scale bar, 50 µm. **E** Panel 1 shows a schematic of PC organization. Scale bar is 1 mm. Panel 2 presents a five-fold magnification of the PC layer (100 weeks) stained with DAPI (blue) and calbindin (red); green represents lipid autofluorescence. Higher magnification of cells in panel 2 with (panel 3) or without (panel 4) lipofuscin autofluorescence. Scale bars for panels 2–4, 10 µm. **F** Left: overlay of DAPI (blue), NeuN (green), and γH2AX (red) in a magnified striatal neuron from an HD mouse (70–90 weeks). Right: γH2AX alone. Scale bar, 5 µm. **G** Same as (**F**) but co-stained with two DSB markers in the same cell. Scale bar is 5 µm. Single-cell quantification of γH2AX intensity in NeuN(+) neurons and NeuN(–) glia from STR and CBL of WT (green) and HD (red) mice at 7–10 weeks (**H**) and 70–90 weeks (**I**) (*n* = 3 per genotype). Significant increases in HD neurons were detected by one-way ANOVA (****p = 0.0001). Results for γH2AX and 53BP1 staining were reproducible (Supplemental Fig. 10A, C). **J**, **K** Comet assays for DNA DSBs in STR and CBL at 70–90 weeks. **J** Representative comet images and assay schematic are shown. Scale bar, 10 µm. (see also Supplementary Fig. 10D, E). **K** Tail moments were calculated using imaging software. Quantification from STR (*n* = 3 WT, *n* = 3 HD; ~300 comets per mouse (source files) shows significantly increased DSBs in HD mice (****p = 0.0001).

If inhibition of DSBR contributed to HD toxicity, we expected DSBs to be elevated beyond normal levels in neurons of *HdhQ(150/150)* mice relative to WT animals. Furthermore, we expected that DSBs would accumulate to the highest extent in the STR of the disease animals with age. We probed for them using additional DSB-associated markers (Fig. 6A–G). Indeed, in the STR (Fig. 6A) and CBL (Fig. 6B) of whole-brain sections, neurons in *HdhQ(150/150)* mice exhibited nuclear and perinuclear staining for phosphorylated ATM-dependent KRAB-associated protein 1 (pKAP-1)^92^, which cooperates with γH2AX in recruiting chromatin-remodeling activities (Fig. 6C, D). pKAP-1 staining was prominent in the STR of *HdhQ(150/150*) mice (Fig. 6C, HD) relative to WT mice (Fig. 6C, WT) and increased with age in animals from 7 to 100 wks in all brain regions. In the CBL of *HdhQ(150/150)* animals (Fig. 6D), pKAP-1 staining was largely restricted to a single layer of large cells adjacent to a dense neuronal layer (Fig. 6E, panel 1), which stained with calbindin (Cal), a marker of Purkinje cells)^61^ (Fig. 6E, panels 2–4). Calbindin and pKAP-1 staining was accompanied by significant autofluorescence (Fig. 6E, green) from oxidized lipofuscin lipids, as reported previously)^7^ Thus, striatal neurons—preferentially targeted for toxicity—accumulated pKAP-1 with age, whereas staining was infrequent in more resistant cerebellar neurons (Fig. 6D).

To confirm that pKAP-1 staining reflected DSBs, IF experiments were repeated using γH2AX^93^ and 53BP1^94^ antibodies (Fig. 6F–I). γH2AX forms extended chromatin domains surrounding DSBs, while 53BP1 promotes NHEJ through the Shieldin complex and suppresses HR. Indeed, γH2AX staining in 70-90 wk animals co-localized with NeuN-positive neurons (Fig. 6F), and γH2AX-positive cells also stained with 53BP1 (Fig. 6G). Co-staining was reproducible in *n* = 4 regions in each brain slice and quantified in around 50 neurons (NeuN(+)) cells and 50 glia (NeuN(-)) in the STR and in the CBL of WT and *HdhQ(150/150*) animals at young and old ages (Fig. 6H, I and Supplemental Fig. 10A–C). The γH2AX signals from the tissue of test animals per genotype were displayed in box plots (WT or HD), where each dot is one scored γH2AX-stained cell. DSB levels were modest in the CBL of both genotypes at all ages (Fig. 6H, I, CBL) but increased significantly in NeuN(+) striatal neurons of *HdhQ(150/150)* (red) mice relative to WT(green) at both ages (Fig. 6I, STR). These findings were highly reproducible across *n* = 4 animals using either γH2AX (*n* = 3 in Supplemental Fig. 10A,B) or 53BP1 antibodies (*n* = 1 in Supplemental Fig. 10C). Digital images (using Fiji) identified DSBs in both neurons (red) and glia (blue) within large fields of cells, but few foci were obvious in NeuN(-) glia (Supplemental Fig. 10B).

To confirm that staining with γH2AX and 53BP1 antibodies reflected actual broken DNA, it was measured directly using a neutral comet assay^95,96^ in the STR of *n* = 3 animals of each genotype at 70–90 wks (Fig. 6J, K). We have previously reported detailed analysis of CometCHIP applied to the normal mouse brain and these published methods were used here (see “methods”)^88^. Dispersed striatal cells from both genotypes were subjected to electrophoresis under neutral conditions, producing comet-like structures proportional to DNA breakage (Supplemental Fig.10D, E). The computer software delineates the comet heads and tails in digital images of neurons and glia and quantifies their intensity (Fig. 6J and Supplemental Fig. 10B). Physical examples of comets from dispersed tissue are shown in gel images (Supplemental Fig. 10D) or as magnified images (Fig. 6J and Supplemental Fig. 10E) for both genotypes. The number of breaks is estimated by the % DNA in the trailing tails and reported as comet tail moments (Fig. 6K)^88^. At least 100 comets per genotype were pooled from the gels of *n* = 3 animals and displayed together (around 300) in the box plot, where each dot is one scored comet (Fig. 6K, 70–90 wk animals). Consistent with the IF analysis (Fig. 6H, I), comets tails were elevated in the affected STR of HD animals relative to WT at 70–90 wks (Fig. 6K). Thus, DSBs co-existed with somatic CAG expansion in *HdhQ(150/150)* mice, appeared earlier (7–10 wks) than the onset of expansion (~12 wks)^31^, and preceded both motor dysfunction (~20 wks) and neurodegeneration (~60 wks)^7^ in *HdhQ(150/150*) mice (Fig. 1A).

### DSBs rise concomitantly with transcriptional pathology, independent of somatic CAG expansion

**Fig. 7:**
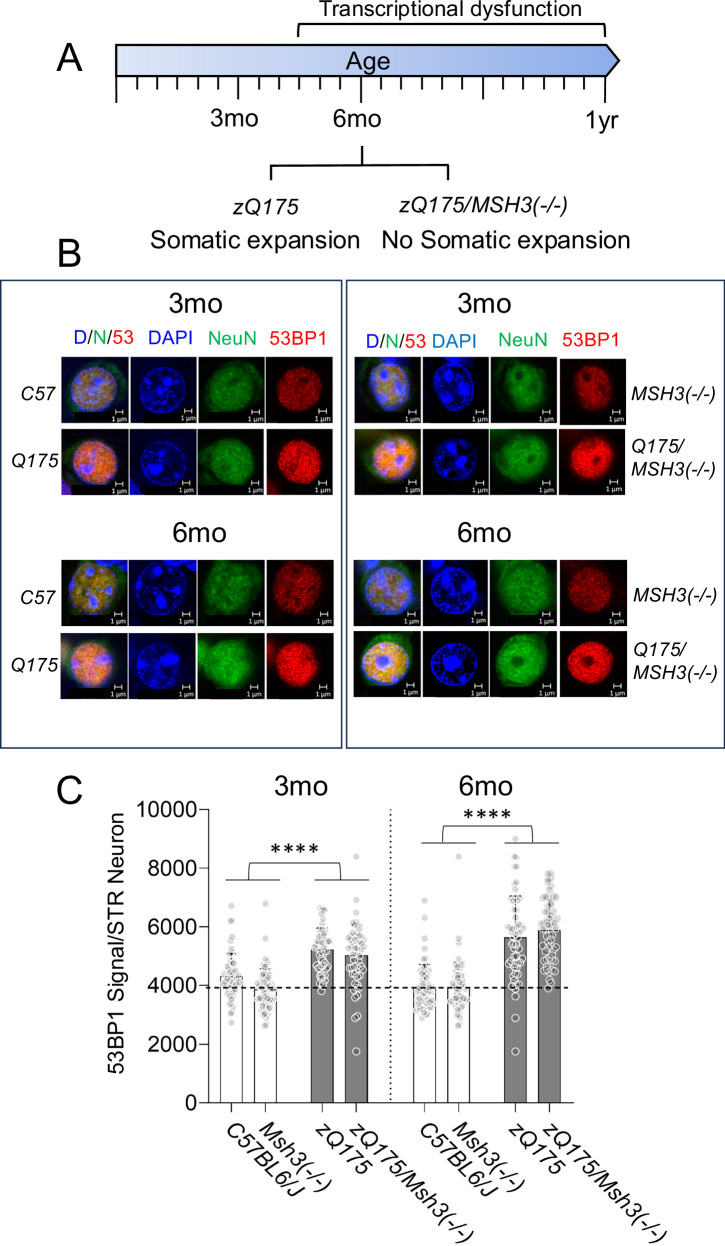
DSBs increase together with progressive transcriptional dysfunction in *zQ175/MSH3(-/-)* animals that cannot expand their CAG tract. **A** Summary of measured pathology and somatic expansion quantified in *zQ175* and *zQ175/MSH3(-/-)* animals as previously published^62,63^. Somatic expansion is attenuated in *zQ175/MSH3(-/-)* animals. **B** DSBs were detected by 53BP1 antibody staining in neurons in brain tissue sections from *n* = 3 animals per genotype: *C57BL/6 (C57), zQ175, MSH3(-/-), and zQ175/MSH3(-/-)* animals at 3 mo (top) and 6 mo (bottom). Similar results were obtained in *n* = 4 regions in each tissue slice per genotype. All IF signal intensities are expressed in arbitrary units. (Left) neurons from WT and zQ175 mice and (right) neurons from MSH3(-/-) and *zQ175/MSH3(-/-)* mice, as indicated. Individual channel images for nuclear DAPI (blue) (panel 2), NeuN neuronal marker (panel 3, green), the 53BP1 DSB marker (panel 4, red), and an overlay of all three (D/N/53, panel 1). The genotypes are designated on the sides of the IF images. Scale bar, 1 µm. **C** Quantification of DSBs in the genotypes from (**B**) as measured by 53BP1 staining intensity. Plotted are the mean ± SD for at roughly *n* = 50 cells per genotype across *n* = 4 regions of each section per genotype. Black bars represent DSBs in *zQ175* and *zQ175/MSH3(-/-)* animals at 3 and 6 months; white bars represent the comparable genetic controls, *C57* or *MSH3(-/-)*, respectively. Error bars indicate standard deviation. Significance was determined using a one-way ANOVA (****p = 0.0001) for all comparisons.

Since they occurred together with somatic CAG expansion in *HdhQ(150/150*) mice, it was not possible to fully determine whether DSBs contributed independently to toxicity in this line. Thus, we constructed two in vivo experiments to test whether somatic expansion and DSBs could act independently as drivers of neuropathology. In the first experiment, we used *zQ175*^62^ and *zQ175/MSH3(-/-)* mice^63^ as separation of function mutants to segregate the impacts of the two types of damage (Fig. 7). Both *zQ175*^62^ and *zQ175/MSH3(-/-)* mice harbor a CAG tract of 175 and exhibit significant HD-like phenotypes and seizures^62^. Because expansion requires MSH3, however, only *zQ175*^62^ can undergo somatic expansion (Fig. 7A). Although neurons do not die at early ages in either strain (Supplemental Fig. 11), both *zQ175*^62^ and *zQ175/MSH3(-/-)* mice develop transcriptional dysfunction and protein aggregation as early pathology by 6 months^62,63^. (Summarized in Fig. 7A). Thus, transcriptional dysfunction does not depend on somatic expansion in these animals.

To test whether DSBs could act as independent drivers of neurotoxicity, we asked whether they were present in *zQ175* and *zQ175/MSH3(-/-)* mice. DSB level was measured in *zQ175* and *zQ175/MSH3(-/-)* mice and compared to their genetic background controls by the staining intensity of antibodies to γH2AX (green) and 53BP1(red) (Fig. 7B), compare *C57Bl6* to *zQ175* (on left) and *MSH3(-/-)* to *zQ175/MSH3(-/-)* (on the right). Representative examples of the antibody staining are shown at 3 and 6 months (Fig. 7B). In magnified cells from the tissue fields, DSBs were measurable in all four mouse genotypes at any age, as detected using either γH2AX or the 53BP1 markers (Fig.7B). The same pattern was reproducible in all regions of the tissue. However, γH2AX (green) and 53BP1(red) staining intensity was higher in *zQ175* and *zQ175/MSH3(-/-)*, which express mhtt, compared to their respective genetic controls at either 3 (upper panels) or 6 (lower panels) months (Fig. 7B, compare *C57Bl/6 J* to *Q175* and *MSH3(-/-)* to *Q175/MSH3(-/-))*.

To quantitatively compare the DSBs in all four genotypes, we measured them at the single-cell level in the STR by quantifying their 53BP1 IF intensity in at least 50 NeuN (+) cells randomly selected over tissue field from *n* = 3 animals per genotype per age (Fig. 7C). Results were plotted as mean ± standard deviation, where each dot in the plot is a scored 53BP1-stained cell (Fig. 7C). Mhtt-expressing strains, (*zQ175* and *zQ175/MSH3(-/-)*), were represented as black boxes, while the genetic background controls strains were represented as white boxes (Fig. 7C). DSB levels in control strains were similar and did not change significantly with age (Fig. 7C, white boxes). In contrast, both mHTT-expressing strains (*zQ175* and *zQ175/MSH3(-/-)*) exhibited elevated DSB levels that were higher in disease animals at 3 months and increased by approximately 30–50% by 6 (Fig. 7C, black boxes). MSH3, which is necessary for somatic expansion, did not promote the DSBs since they were present in *zQ175/MSH3(-/-)* animals that could not expand. Whether DSBs promoted the transcriptional dysfunction was not tested in the experiment. However, DSB accumulation coincided with transcriptional dysfunction in both *zQ175* and *zQ175/MSH3(−/−)* mice, regardless of the presence or absence of somatic expansion. Thus, DSBs and somatic expansion acted as independent pathways in disease cells.

### XJB-5-131 treatment rescues DSBs and toxicity in *HdhQ(150/150*) animals, with minimal effects on somatic expansion

**Fig. 8:**
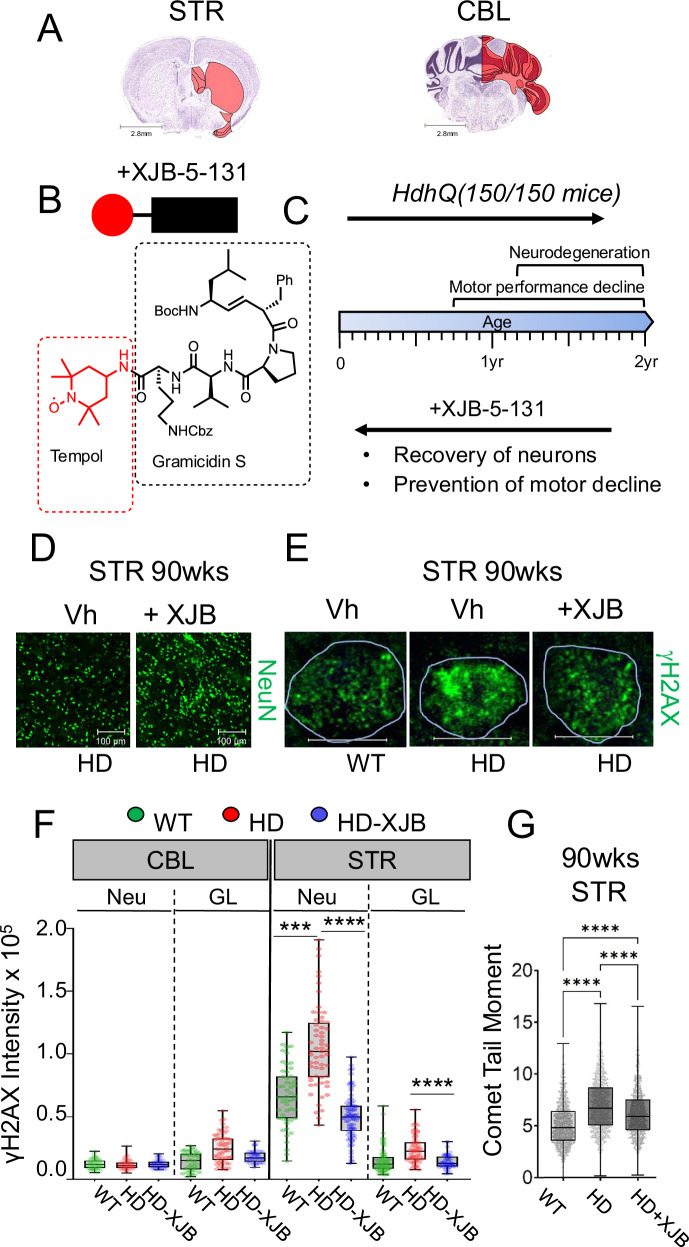
XJB-5-131 treatment of *HdhQ(150/150*) mice attenuates DSBs and prevents neuronal death without significant suppression of somatic expansion. **A** Allen Brain Atlas^136^ map highlighting the affected striatum (STR, red; left) and the resistant cerebellum (CBL; right) for reference^136^. **B** Schematic and chemical structure of the ROS inhibitor XJB-5-131. The antioxidant tempol moiety (red) is linked to a mitochondria-targeting carrier peptide derived from gramicidin S (black), enabling localization to mitochondrial membrane. XJB-5-131 prevents base oxidation and suppresses SSB-to-DSB conversion⁸⁷. Untreated WT or HD mice (*n* = 3 per genotype) were aged to 60 weeks to allow DSB accumulation, then treated with saline vehicle (Vh) or XJB-5-131 for 30 weeks until sacrifice at 90 weeks. **C** Summary of DSBs and reversible pathology in HdhQ(150/150) mice before and after XJB-5-131 treatment⁷. **D** Representative striatal sections from 90-week-old HD mice treated with vehicle or XJB-5-131, stained for NeuN (green). XJB-5-131 rescues neuronal loss, indicated by increased the NeuN IF signal⁷. Scale bar, 100 µm. **E** Magnified γH2AX stained striatal neurons in tissue from WT (Vh), HD (Vh), and HD + XJB-5-131 mice in (D). Scale bar, 10 μm. **F** Similar patterns were observed across *n* = 4 regions per section. Single-cell quantification of γH2AX IF intensity in NeuN(+) neurons and NeuN(–) glia from STR and CBL of WT (Vh, green), HD (Vh, red), and HD + XJB-5-131 (blue) mice (*n* = 3 per group). Approximately 50 cells per region per cell type were analyzed and displayed as box-and-whisker plots (box = middle 50%, line = median, whiskers = min/max quartiles). Significance was assessed by one-way ANOVA (*** *P* = 0.001; **** *P* = 0.0001). **G** Neutral comet assay for samples in (**F**). Dispersed brain cells from 90-wk old animals were analyzed for DNA breaks (Methods). Approximately 300 comets were quantified per genotype. Comet tails increased in Vh-treated HD mice compared with WT controls and were reduced by XJB-5-131 treatment (HD + XJB-5-131). Additional comet images are shown in Supplemental Fig. 13. Data are presented as box-and-whisker plots as in (F); significance was evaluated by one-way ANOVA (**** *P* = 0.0001).

If DSBs were an independent driver of HD toxicity, then reducing DSB formation should be beneficial, even in the presence of ongoing somatic expansion. Thus, in a second in vivo experiment, we suppressed DSBs using XJB-5-131 and tested whether toxicity was reversible in the STR (Fig. 8A) in a second strain of HD mice (Fig. 8*HdhQ(150/150*)). XJB-5-131 (Fig. 8B) targets the mitochondrial membrane and suppresses ROS and SSB to DSB conversion^88^. Indeed, XJB-5-131 treatment has been shown to suppress DSB formation in the brains in normal mice^88^ and to reduce DSB formation in peroxide treated NIH3T3 cells^88^. XJB-5-131 treatment blocked the degradation of mitochondrial DNA^64,65^, prevented the decline in motor function, and blocked decline of neuronal number (observed at 60 wks) in *HdhQ(150/150*) animals (Fig. 8D)^7^ relative to comparable saline treated (Vh) animals^7^. To determine whether DSBs and somatic expansion had separable impacts on reversible pathology, we measured them together in the same tissues during rescue^7^ (Fig. 8D-G). HD or WT animals were aged to 60 wks to allow the formation of DSBs and treated for an additional 30 wks with either XJB-5-131 or a saline vehicle (Vh) control. The animals were sacrificed at 90 wks and tested for the level of DSBs. DSBs were estimated by antibody staining with γH2AX (green in Fig. 8E, F). Representative examples of γH2AX staining are shown in magnified cell images of the STR (Fig. 8E, green), but the same pattern was observed throughout the tissue fields. Indeed, DSB increased in the STR of *HdhQ(150/150*) animals treated for 30 wks with saline (Fig. 8E, HD-Vh) relative to WT animals (Fig. 8E, Vh-WT) and were reduced by XJB-5-131 treatment for the same period (Fig. 8E, HD + XJB-5-131). This occurred in the same tissues where XJB-5-131 treatment of *HdhQ(150/150*) animals blocked the decline of striatal neurons^7^ (Fig. 8D, HD + XJB-5-131). Although XJB-5-131 suppressed DSBs (Fig. 8E) and neuronal loss in the STR (Fig. 8C, D, NeuN staining, green), it had no substantial impact on somatic expansion (Supplemental Fig. 12). Thus, suppression of DSBs rescued neuropathology in *HdhQ(150/150)* mice independently of somatic expansion.

The cell type distribution of DSBs was quantified in the same tissue sections by γH2AX IF intensity in roughly *n* = 50 NeuN(+) neurons and *n* = 50 NeuN(-) glia in the STR and in the CBL of both genotypes in both treatment groups (Fig. 8F). DSB levels were low in the resistant CBL of either WT or *HdhQ(150/150)* mice (Fig. 8F, CBL) and in glia from the affected STR of *HdhQ(150/150)* animals (Fig. 8F, STR GL). However, DSBs doubled in striatal neurons in Vh-treated *HdhQ(150/150*) animals (Fig. 8F, Neu STR, red) relative to comparable Vh-treated WT controls (Fig. 8F, STR, green), and XJB-5-131 treatment inhibited the increase (Fig. 8F, blue). To confirm that γH2AX staining reflected actual DNA breaks, DSBs were measured directly by neutral comet assay in dispersed cells from the same tissues (Fig. 8G). Indeed, comet tail moments were reversible by XJB-5-131 in the STR of *HdhQ(150/150*) animals (Fig. 8G)(Supplemental Fig. 10D). A larger set of amplified images for XJB-5-131 comet reversibility are shown (Supplemental Fig.13). DSBs were rescued in the comet analysis and reflected the same XJB-5-13-reversible pattern as did γH2AX staining (Fig. 8F, STR, Neu). The reduction in DSBs by XJB-5-131 promoted rescue of disease toxicity, e.g., XJB-5-131 increased NeuN staining and improved motor performance without substantial impact on somatic tract length (Supplemental Fig. 12). Thus, DSBs were key drivers of reversible neuropathology independent of somatic expansion.

## Discussion

**Fig. 9:**
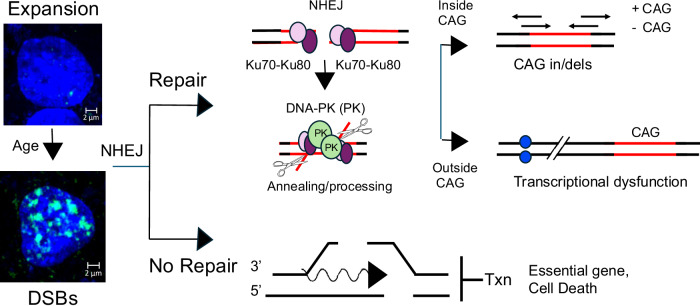
Model for the separable impacts of DSBs and somatic expansion on HD neuropathology. (Left) Cells with an expanded CAG tract (upper) develop DSBs with age (lower) as indicated by accumulation of γH2AX foci staining. Scale bar is 2 µm. (Right) Model for the role of DSBs in promoting HD toxicity, as discussed in main text. (Repair, NHEJ). NHEJ uses Ku70/Ku80 dimer to recruit DNAPK and the nuclease Artemis (scissors) to prepare two broken DNA ends for ligation. Extensive DNA end resection is prevented by Ku binding, and Artemis (scissors) typically removes short regions of DNA to expose patches of microhomology. (Inside CAG tract) If the DSBs occur within a CAG repeat tract (red lines), microhomology is high and nuclease processing results in no change, or small insertions/deletions of CAG repeats (small arrow). (Outside CAG tract) DSBs occur preferentially outside the CAG tract in random genomic DNA (black lines) in HD animals and therefore have no expected impact on CAG tract length. In the absence of homologous recombination, annealing is imperfect at random genomic sequences (black). Artemis typically removes short regions of DNA to remove incompatible bases to improve homology, often resulting in somatic mutations in the genome (blue circles). The resulting somatic mutation occurs most frequently in gene enhancers. influencing transcriptional regulation^97^. (No repair) If an unrepaired DSB occurs within a gene that is essential for life, transcription will terminate and cause death. Thus, an outcome of transcriptional dysfunction or death, respectively, depends on whether error-prone NHEJ is successful at repairing the damage and on the location of the DSBs (Fig. 9, Repair and No repair).

Here, we report that double-strand breaks (DSBs) drive neuropathology in *HdhQ(150/150)* mice both in the presence and absence of somatic CAG expansion. Although DSBs and somatic expansion co-exist in the HD brain, these two forms of DNA damage promote disease through distinct mechanisms. Active mismatch repair (MMR) mediates site-specific increases in CAG tract length within the HD allele. Somatic expansion does not require DSBs and proceeds independently if MSH3 and its associated factors are expressed (Fig. 7). Conversely, a reduction of DSBR activity drives DSB formation, which accumulates throughout the genome. MMR is not required for the formation of DSBs (Fig. 7), as they accumulate in HD animals that exhibit pathophysiology but are incapable of somatic expansion (Fig. 7). Importantly, suppression of DSBs by XJB-5-131 is sufficient to reverse neuropathology (Fig. 8) even when somatic expansion remains active (Supplemental Fig. 12). We propose that CAG expansion and DSBs are separable drivers of disease pathology: disease-length CAG tracts promote early inhibition of double-strand break repair (DSBR; Fig. 5), leading to progressive accumulation of DSBs that ultimately trigger neuronal death (Figs. 6 and 8). Since somatic expansion is not required for neurotoxicity, DSBs and inhibition of DNA repair are likely to depend most on the inherited disease-length allele, with somatic expansion exacerbating the effect over time. Because XJB-5-131 suppresses DSBs and reverses toxicity, DSBs emerge as both prominent drivers of neuropathology and as independent targets of therapeutic rescue. Previously, modest effects on CAG expansion from loss of DSBR machinery argued against a central role for DSBs in HD. However, three key features establish a specific and independent role for DSBs in driving disease toxicity in *HdhQ(150/150)* mice, separate from somatic expansion (Fig. 9).

First, if a DSB occurs inside a CAG tract, there is no expectation that its length will substantially change if repaired by NHEJ (Fig. 9, Repair, inside CAG tract). NHEJ pulls together DNA to find homology for annealing and ligation of the broken ends^97–100^. Within repetitive DNA (Fig. 9, red lines), microhomology is high and annealing may be accurate. If overhangs are formed at single-strand/double-strand junctions during annealing, the Artemis nuclease (Fig. 9, scissors) removes them^97–100^, including fold back structures that may arise between the two DNA ends being joined^97,99^. Importantly, Ku70-Ku80 blocks removal of large DNA segments (typically limiting changes of 4 nucleotides or less)^97,99^. Thus, at CAG tracts, repair by NHEJ is expected to result in only small CAG insertions or deletions (Fig. 9, repair inside CAG, in/dels), with contractions being more likely than expansion if the overhangs are removed. HR does not compensate for NHEJ since there is no homologous chromosome in non-dividing neurons. Consistent with this expectation, CGG repeat expansion in a fragile X model is independent of HR-related machinery including Pol θ, RAD52, RAD54 or RAD54B^101^, and loss of Rad 52 or Rad54 has little effect on somatic CTG expansion in the brains of myotonic dystrophy type 1 (DM1) mice^102^.

Second, if DSBs form outside the CAG tract, they will have no impact on its length (Fig. 9, repair outside CAG). Indeed, we find that estimates of unrepaired DSBs in the striatum of 70–90 wk *HdhQ(150/150*) mice are on the order of 10^5^ (Fig. 6I), yet the expanded CAG repeat tract at the HD locus represents less than 10^−7^ of genome. The number of DSBs is likely to be higher than estimated since a snapshot of time does not include those that have been made and repaired. Based on their frequency, some, if not most of the DSBs will reside outside the CAG tract (Fig. 9, repair outside CAG). In random genomic DNA, however, homology for annealing and ligation of the broken ends is likely to be poor (Fig. 9). Artemis processing during NHEJ can improve homology by removing incompatible sequences (Fig. 9, scissors), but error-prone repair of a DSB often occurs at the expense of introducing nucleotide variants at the repair junction in somatic cells (Fig. 9, repair outside CAG, blue circles)^97–99,103^. Indeed, error-prone NHEJ is a common source of nucleotide variants throughout life and often alters gene transcription.

DSBs can arise from multiple processes. However, in the absence of a replication fork, random endogenous base damage (alkylation, oxidation or deamination) is a major source of damage^84,104^. During removal of chemically modified DNA bases, BER produces transient SSB intermediates^104^, which can convert to a DSB if closely spaced SSBs lie simultaneously on opposite sides of the DNA helix^88^. Indeed, recent sequencing technologies confirm that SSBs are closely clustered in the enhancers of neuronal genes. Repair-Seq^105^ or Synthesis-associated with repair sequencing (SAR-Seq)^106^ utilizes incorporation of the thymidine analog EdU to provide a biotinylated capture tag for DNA damage. In neurons, SAR-seq peaks co-localized with Poly (ADP-ribose) polymerase (PARP) and X-Ray Repair Cross Complementing 1 (XRCC1), which is a protein scaffold required for BER and SSBs^106^. The clustered SSBs suggest that conversion to DSBs may occur most frequently in transcriptional regulatory sites and error-prone repair may leave behind somatic mutations^107,108^ which may alter gene activity^109–111^.

Transcriptional dysfunction is a prominent feature of neuropathology in both HD patients and in mouse models^60,112–114^. Whether NHEJ-mediated nucleotide variants and DSBs drive transcriptional dysfunction in the genomes of HD patients remains to be validated. However, our results from “separation of function” mice confirm that DSBs accumulate together with progressive transcriptional dysfunction in *zQ175/MSH3(-/-)* animals that cannot expand their alleles somatically (Fig. 7). The patterns of transcriptional alterations in HD are somewhat reproducible^60,112–114^, which is unlikely to derive from largely random DSBs throughout the genome. However, clustered signatures of single nucleotide variants (SNVs) display recurrent patterns of damage that is shared among neurodegeneration diseases^115,116^ at hotspots for SSBs^105,106,117,118^, DSBs^119,120^, and oxidation^121–123^. Thus, over decades, we envision that DSBR at preferred sites is likely to provide at least some reproducible somatic variants in active genes, while the effects of random DSBs forming at other locations would be too small to be detected.

Third, because NHEJ is inefficient in mhtt expressing cells, this pathway sometimes fails to correct DSBs (Fig. 9, No repair). Unlike cancer cells, persistent DSBs in non-dividing cells are tolerated if a gene is dispensable, redundant, or has functional backup pathways. However, as DSBs increase in *HdhQ(150/150*) animals (Fig. 6), they present an increasing risk that unrepaired DSBs will terminate transcription of an essential gene, leading to death (Fig. 9, No repair). Indeed, the HD gene itself is required for life and an unrepaired break there would be toxic. There is substantial evidence that loss of DNA repair leads to persistent DSBs and progressive neurodegeneration in spinocerebellar ataxia with axonal neuropathy (SCAN1), ataxia-oculomotor apraxia 1 (AOA1), and spinocerebellar ataxia-3 (SCA3)^124–129^, among others. While there are no inactivating mutations in DNA repair genes associated with HD, inhibition of end joining activity is a shared feature of neuropathology in both HD and SCA3^130,131^. In the latter, the expanded ataxin 3 gene product suppresses the activity of polynucleotide kinase/phosphatase (PNKP)^132^, inhibiting its end joining activity on nascent RNA of transcribed genes, and elevates DSBs^130,131^. End joining machinery may be a common target of polyglutamine proteins, and DSBs may be a common feature of neuropathology in multiple disorders^133^. Elevated DSBs, when repaired under disease conditions, may alter or disrupt transcription, which contributes to neuronal abnormalities (Fig. 9, transcriptional dysfunction) or lead to neuronal death if DSBs in key genes are not repaired (Fig. 9, no repair).

Although DSBs appear to be independent drivers of neuropathology, the machinery for DSBR has not been identified in GWAS analysis of HD patients. DSBs, however, form randomly throughout the genome at loci outside the CAG tract, and, therefore, will vary among patients. It is also not clear whether a reduction in NHEJ activity can be detected as a classic modifier. Since NHEJ activity is inefficient but not absent, partial effects may not provide sufficiently robust statistical correlations among DSBR genes and disease onset in HD patients. Nonetheless, it is notable that Ligase IV of the NHEJ pathway has been identified recently as a repressor of CAG expansion in a HttQ^112^ mouse CRISPR knockout screen^48^. Since ligase IV can also operate in NHEJ outside the CAG tract, a role for ligase IV in mediating neuropathology may be complex and warrants further experimental clarification. Collectively, we find that DSBs co-exist with CAG somatic expansion but have different effects. Each DNA-dependent process has a distinct driver, a distinct mechanism, and a separable location-dependent impact on toxicity. As such, these two types of DNA damage may be independent therapeutic targets. Our data suggest that simultaneous suppression of somatic CAG expansion and DSBs is likely to be a beneficial therapeutic strategy for HD and may even be necessary to offset disease.

## Methods

### Animals

The Institutional Animal Care and Use Committee approved all procedures. Animals were treated under guidelines for the ethical treatment of animals and approved by IACUC protocol #274005 at Lawrence Berkeley Laboratory. All animal work was conducted according to national and international guidelines. C*57BL/6J* mice were obtained from Jackso Laboratory; the *HdhQ(-/150)* parental strain was a gift from P. Detloff. All experimental results were generated using male mice. Mice are typically maintained on a 12-h light /12-h dark cycle in controlled animal housing rooms. Mouse housing rooms are generally maintained at approximately 22 ± 2 °C. The Relative humidity is usually controlled at 55% in animal housing rooms.

### Breeding and statistical consequences of congenic *HdhQ(150/150)* and C*57BL/6 J* controls lines

For use in this study, we generated a congenic mouse line *HdhQ(150/150)* starting from *HdhQ(-/150)* mice. A congenic line ensures that all animals are genetically identical (clonal)—a process that requires years of careful breeding. The resulting congenic *HdhQ(150150)* strains essentially express the same proteins to the same extent, which significantly suppresses animal to animal variability in DNA repair protein expression in their tissues. To make congenic strains, *HdhQ(-/150)* mice were bred with C57Bl6 and backcrossed for 5 years (>15 generations) to generate *HdhQ(-/150)* congenic mice. The animal line harbors a long CAG repeat tract of roughly 150 “knocked into” the endogenous mouse gene^66^. This line is a late onset model for HD and undergoes progressive somatic expansion and progressive pathology. Motor abnormalities are detected around 20 wks^7,66^; neuronal death in the striatum (STR) was measurable around 60 wks^7^. *HdhQ(150150)* lines were derived from congenic *HdhQ(-/150)* by crossing them and selecting *HdhQ(150150)* progeny in the F1 generation. The *HdhQ(150150)* progeny were crossed for another 3 years to create congenic strains of *HdhQ(150/150)* mice of identical background. The C57Bl6 animals were bred in parallel as a separate strain and were also congenic. As in humans, the onset of somatic expansion in *HdhQ(-/150)* and *HdhQ(150/150)* mice is unaffected by the number of expanded alleles in a 1200 animal study^31^. DSBs in homozygous *HdhQ(150/150)* animals were compared directly to *C57Bl6* lines, which but were bred side by side but had not seen the HD allele for several generations.

### Statistical and sample size considerations for congenic condition

The biological replicates (n) we used in the congenic mouse studies are smaller than the more common condition of variable colony backgrounds but are grounded on well-established statistical principles. By definition, the number of biological replicates (n) is determined based on the expected differences in the outcome. The great value of using a congenic strain lies in its near-zero inter-animal variability. Indeed, because the genetic background is identical in a congenic strain, gene expression profiles are effectively the same across animals. Because each animal is identical, the expected difference in outcome of DNA repair protein expression profiles is negligible across animals, which dramatically reduces the number of animals needed to achieve statistical significance. The statistical considerations are well developed^67^.

For a simple two-group comparison, the biological sample size per group is determined:

1 $$n=\,\frac{2{\sigma }^{2}{({z}_{1-\alpha /2}+{z}_{1-\beta })}^{2}}{{\delta }^{2}}$$

Where:

- $${\sigma }^{2}$$= biological variance
- $\delta$= true mean difference (the size of the expected differences in the outcome)
- $$\alpha$$= significance level
- $\beta$= Type II error (1 − power)
- z= score for the number of standard deviations from the mean that captures probability

In a congenic condition, the samples are identical, and there is no difference in expected outcome among animals, i.e., δ = 0.

2 $$\begin{array}{c}n=\frac{2{\sigma }^{2}{({z}_{1-\alpha /2}+{z}_{1-\beta })}^{2}}{0}\\ \Rightarrow n\to \infty \end{array}$$

An infinite number of samples is needed to disprove the null hypothesis if the strain is congenic (i.e., the observed data are inconsistent with what the null hypothesis predicts (that there is no protein in the sample**)**. Thus, for western experiments in Figs. 1 and 3, where the result is a yes-no binary question, band intensity from antibody staining in two congenic animals is sufficient to determine whether a protein is there or not. One congenic animal establishes the effect (protein is present), which is confirmed in at least one more clonal animal from the colony. No significance is reported. When δ = 0, increasing sample size only increases precision around a null effect. Any “significant” technical replicate would be due to random sampling error, not biology. The statistical logic does not change if the sample is a tissue or cell from a congenic animal. However, in quantitative single cell experiments, comparisons are being made between genotypes, and accuracy matters. In these quantitative experiments, minimally *n* = 3 (up to six in some cases) congenic mice are used to reduce the chance of sampling error. Thus, in Figs. 5–8, we used minimally *n* = 3 (up to six in some cases) congenic mice in the analysis. No behavioral testing is conducted in these studies. However, it is notable that the n needed for measuring protein expression is distinct from biological n that would be needed if behavior were being tested—even for congenic strains. That is because animal handling, testing geometry/equipment, room temperature and testing schedule among others can impose variability, and more animals would be needed for validating significance (i.e., δ is no longer 0).

### Reagents and resources

Reagents used are presented in Supplementary Table 1. Antibody testing results are presented in Supplementary Table 2. Uncropped gel images are provided in Source Data file.

### Brain Tissue Lysate Preparation

Dissected brain tissues were flash frozen in liquid nitrogen and were preserved at −80 °C until lysate preparation. Brain tissue was placed in a microcentrifuge tube and thawed on ice for approximately 10 min before adding lysis buffer consisting of T-PER Tissue Protein Extraction Reagent (Thermo Scientific, Cat No 78510), ~8.7x Halt protease inhibitor cocktail (from 100x, Thermo Scientific, Cat No 78430), ~4.3x Halt phosphatase inhibitor cocktail (from 100x, Thermo Scientific, Cat No 78420). After adding the lysis buffer, the tissue sat on ice for 2–3 min before being triturated with a Bel-Art extended handle pestle (Millipore-Sigma, Cat No BAF199210001). The triturated tissue sample was centrifuged at 16,100 × *g*, 4 °C for 1.5 min in Eppendorf 5425 R refrigerated microcentrifuge. Following the centrifugation, the sample was subject to 4 cycles of pulse sonication on ice, performed in which 2 cycles of 10s-on and 30s-off followed by centrifugation at 16,100 × *g*, 4 °C for 1.5 min, 1 cycle of 10s-on and 30s-off followed by centrifugation at 16,100 × *g*, 4 °C for 1.5 min, and then 1 cycle of 10s-on and 30s-off. Sonication was performed using Branson Sonifier Cell Disruptor 185. The resulting homogenate was centrifuged once more at 16,100 × *g*, 4 °C for 30 min and supernatant was used for the protein quantification assay, SDS-PAGE and western blotting analyses.

### Protein quantification assay

The protein concentration of each lysate was measured using Pierce 660 nm Protein Assay reagent (Thermo Scientific, Cat No 22660). Briefly, lysates were diluted 1:10 in phosphate-buffered saline (PBS). 10 μL of diluted lysate was mixed with 340 μL of Pierce 660 nm Protein Assay reagent in a 1.7 mL microcentrifuge tube and then incubated at room temperature (RT) for approximately 5 min before loading 150 μL of each mixture in each of 2 wells of a flat bottom, transparent 96-well plate. For each lysate, a mixture was prepared and analyzed by Infinite M1000 microplate reader (Tecan) using Tecan i-control software.

### SDS-PAGE and western blot

SDS-PAGE samples were prepared with 60 μg total protein and probed with a test proteins or glyceraldehyde 3-phosphate dehydrogenase (GAPDH), NuPAGE LDS Sample Buffer, and reducing agent. The former was T-PER Tissue Protein Extraction Reagent (1x NuPAGE LDS Sample Buffer (from 4x, Invitrogen, Cat No NP0007) and the latter was 1x NuPAGE Sample Reducing Agent (from 10x, Invitrogen, Cat No NP0009). Each sample was boiled at ~95 °C for 10 min and centrifuged at 15,000 × *g*, at RT for 5 min. Total proteins in each SDS-PAGE sample were subsequently resolved along a Novex WedgeWell Tris-Glycine 4-12 % Mini gel (Thermo Fisher, Cat No XP04205BOX) in XCell SureLock Mini-cell Electrophoresis system (Thermo Fisher). The resolved proteins were transferred to a nitrocellulose membrane using Trans-Blot Turbo Transfer System (Bio-Rad), with a standard protocol (25 V, 1.0 A, 30 min). The resulting nitrocellulose membrane was blocked in 5% non-fat dry milk in PBS + 0.05 % Tween-20 (PBST) for 1 h at RT. The membrane was then incubated in 5 % non-fat dry milk in PBST + primary antibody for 1 h at RT. The membrane was then washed 3 times with PBST. The membrane was subsequently incubated in 5 % non-fat dry milk in PBST + secondary antibody for 30 min at RT. The membrane was next washed 3 times with PBST and once with PBS. Lastly, Amersham ECL Select Western Blotting Reagent (Sigma-Aldrich, Cat No GERPN2235) was applied to the membrane and the western blot image was developed using VersaDoc MP 4000 Imaging System (Bio-Rad) and Quantity One 1-D Analysis software (Bio-Rad). Band quantification was performed by phosphporimaging using Image Lab software (Bio-Rad). The specific antibodies used in the analysis are listed in key resources Supplementary Table 1 and antibody testing is provided in Supplementary Table 2.

### Immunoprecipitation

Brain regions or cultured cells were lysed and homogenized, clarified by centrifugation, and incubated with Dynabead Protein G conjugated to anti-Htt or control IgG antibodies. Brain regions from mice (striatum and cerebellum) or cell pellets from mouse cell cultures were used to isolate proteins associated with mouse huntingtins protein (Htt). Briefly, cell tissue (15–20 mg) or cell pellets (from 80% confluent cultures in T75 flasks) were minced and lysis in buffer (1 mL Pierce IP Lysis Buffer (ThermoScientific cat #87787), with 20 µL Halt Protease Inhibitor (ThermoScientific cat # 78429) on ice using homogenization with a pestle grinder (Kontex Pellet Pestle Grinder, VWR cat# KT749521-1500). Homogenization was done with 40–50 up and down strokes per sample, followed by vortexing at maximum speed (20 s each, x2). The suspension was clarified using centrifugation (spun down at 12,000 × *g* 15 min at 4 °C) and the supernatant was used for protein pulldown. For bead attachment, 50 µL of Invitrogen Dynabead Protein G (ThermoScientific cat#10-004-D) was added to the supernatant on ice, and briefly vortexed. The beads were then immobilized on a magnetic stand, the supernatant removed, and a solution of 1:10 dilution of capture antibody was added (dissolved in 100 µL Dynabead Wash/Binding buffer with 2x Halt Protease Inhibitor). Capture antibody was either anti-Htt mixture (1:1:1 of ThermoScientific cat#MAB-2166, MAB-2168, and MAB2170) or a control antibody (mouse anti-Goat IgG, ThermoScientific cat# 31107). Proteins were incubated for 18 h at 4 °C using rotating mixing (Intellimixer RM-2L, Thomas Scientific cat#1185W35). The beads were washed twice with 100 µL and stored ice for MS as described and performed by the UC Davis Core Facility (https://proteomics.ucdavis.edu/).

### Mass spectrometry (https://proteomics.ucdavis.edu/)

Protein digestion, DIA mass spectrometry, and data analysis were performed using Spectronaut 17 with a targeted library-based approach against the Uniprot Human database supplemented with contaminants. Protein intensities were summarized using the MaxLFQ algorithm. Proteins from the bead pull-down samples were alkylated without reduction and incubated with Lys-C/trypsin mixture (Promega, V5073) (200 ng for pull-down samples and 1 µg for total lysate samples) for 2 h at 37 °C. Afterwards, the samples were diluted to decrease the maximum concentration of urea (up to 1 M) and supplemented with additional amounts of sequencing-grade trypsin (Promega, V5111) (200 ng for pull-down samples and 1 ug for total lysate samples), followed by overnight incubation of the samples at 37 °C. Before mass spectrometry analysis samples were de-identified and randomized. Peptides were directly loaded on an Evosep C18 tip and separated using the Evosep One with the 100 spectral power distribution (SPD)-High. For proteomic data analysis, Data-independent acquisition (DIA) mass spectrometry data were analyzed with the Spectronaut 17 software package (Biognosys) using the targeted library-based approach. A spectral library was generated by searching the data for both pull-down and total lysate samples using the Spectronaut pulsar search engine with the default setting against the Uniprot UP000005640 Human database (20,607 entries) supplemented with the common contaminants database (38 entries). Pulldown samples were separately matched with the resulting library using the Spectronaut default settings. Briefly, “trypsin/P specific” was selected to allow for two missed cleavages. Fixed modifications were set to cysteine carbamidomethylation, and variable modifications were set to peptide N-terminal acetylation and methionine oxidation. For DIA search parameters, PSM and Protein Group decoy false discovery was set to 1%. Protein level intensities were summarized using the MaxLFQ algorithm. The integrated area of the eluted peptides relative to columns standards were used to compare native WT(green), and HD(red) striatal tissue and the endogenous levels in NIH3T3 cells (Light green) alone or NIH3T3 cells overexpressing htt and mhtt (which were prepared separately but evaluated in parallel on the same day). University of California at Davis commonly deposits datasets in ProteomeXchange Consortium partner PRIDE repository.

### Brain tissue sections from *HdhQ(150/150)* and C57Bl6 animals

Brains from male mice were cut into 4 coronal sections and arranged in a holder filled with OCT (Tissue-Tek O.C.T. from Sakura) and immediately frozen in isopentane bath cooled by liquid nitrogen, prior to storage at −80^o^C. This arrangement of the tissue permitted concurrent cutting of all 4 sections at a time. Cuts were made so that all the relevant regions (caudoputamen of striatum, CA1 region of hippocampus, the granular and molecular layer of Crus1 of the cerebellum, and the entorhinal area of the cortex) were present in each cut. Sectioning onto slides (Histobond from VWR) was performed on a cryostat (Leica CM1950) using cut settings (chuck = −14 °C, blade = −15 °C) and cutting 10–15 μm thick sections. Slides were air dried (15 min) and stored at −80 °C until use. In parallel, the dissected tissue of equal weight before freezing was dispersed for cell number counting on a hemocytometer and comet assays.

### Mouse brain fixation and sectioning of *zQ175 and zQ175/Msh3(-/−)* mice and controls

Tissue slices from the *MSH3(-/-), zQ175* and *zQ175/MSH3(-/-)* mice were provided as a gift from G. Bates. The received tissue sections from zQ175 mice were transcardially perfusion fixed with 4% paraformaldehyde (Pioneer Research Chemical Ltd). Brains were removed and further post-fixed for 6 h at 4 °C before cryoprotection with gradient steps of 20% and 30% sucrose in 0.01 M PBS (Sigma). Once the brains reached equilibrium (where they sunk to the bottom of the sucrose solution), they were washed in PBS, embedded in OCT (CellPath Ltd.) and stored at −80 °C. Coronal brain sections were cut at 30 μm on a cryostat and stored free floating at −20 °C in tissue protective solution [30% ethylene glycol, 25% glycerol and 0.05% sodium azide in PBS] until staining. Free floating sections were gently brushed onto glass slides and dried before staining with DSBR antibody markers.

### Antibodies for Immunofluorescence

IF intensity in all Figures is reported in arbitrary units. Antibodies used are listed in Supplemental Table 1. Primary antibodies included mouse anti-NeuN Alexa-488 conjugate (Millipore #MAB377X) (1:500), mouse anti-GFAP Cy3 conjugate (Abcam #ab49874) (1:500), mouse anti-APE1 (Novus #13B8E5C2) (1:500), mouse anti-Ku80 (Santa Cruz #515736) (1:500), mouse anti-ERCC1 (Santa Cruz #17809) (1:500), rabbit anti-MSH2 (Abcam #92473) (1:500), mouse anti-MSH3 (Millipore #MABE324) (1:500), rabbit anti-MSH6 (Abcam #ab92471) (1:500), rabbit anti-XPA (AbClonal #A1626) (1:500) and rabbit anti-MRE11 (Novus #NB100-142) (1:500). (Table S1) Secondary antibodies used were donkey anti-mouse Alexa-488 (Jackson #715-545-150), goat anti-mouse Alexa-568 (Invitrogen #A21124), donkey anti-rabbit Alexa-488 (Jackson #711-545-152), and goat anti-rabbit Alexa-555 (Invitrogen #A32732). Anti-mouse antibodies were tested (Supplemental Table 2) and selected for those having the least amount of background staining, which was typically visible as staining of blood vessels amongst various commercially available options.

### IF measurement of DSBs in tissues

IF intensity in all Figures is reported in arbitrary units. Methods are the same as for IF detection of proteins except an antibody for γH2AX or 53BP1 and NeuN were used (Supplemental Table 1). We note that the 53BP1 antibody marker (and not phospho-53BP1) was used in all DSBR experiments and is indicated by the label 53BP1. Brain sections on slides were thawed and fixed with 4% PFA for 20 min at 4 °C, then washed once with PBS. They were then pre-extracted with RNase in CSK buffer, i.e., 0.3 mg/mL RNase A (New England Biolabs) in (10 mM PIPES pH 7.0, 100 mM NaCl, 300 mM sucrose, 3 mM MgCl_2_, 0.7% Triton X-100). Lipofuscin autofluorescence was quenched by soaking in 1x TrueBlack (CellSignal #92401) in 70% EtOH (30 s) (care was taken not to allow sections to dry out), prior to 3 washes in PBS. Sections were then blocked with Fc Receptor Blocker (Innovex #NB309) for 15 min at RT and then with Background Buster (Innovex #NB306-50) for 15 min at RT, prior to washing once with PBS. Sections were then coated with 200 μL of primary antibodies (1:500 diluted 1:500 in 10% Background Buster: PBS) and incubated for 1 h at 37 °C or overnight at 4 °C, prior to 2 washes of 10 min each with PBS. Sections were then stained with 200 μL of secondary antibodies (1:1000 diluted in 10% Background Buster: PBS) and DAPI (10 μg/mL) for 30 min at 37 °C. Finally, the slides were washed 2 times with PBS, 15 min each, and refixed with 4% PFA for 10 min. Sections were mounted using Immu-Mount (Epredia) and #1 coverslips (Electron Microscopy Services), sealed with nail polish, and stored at −20 °C until they could be imaged. DSBs in neurons were detected as co-staining of γH2AX and NeuN. Quantification of IF staining intensity for γH2AX in NeuN (+) and NeuN(-) cells was determined from 25-70 randomly selected cells within the tissue sections.

### Brain cell preparations for CometChip assay

WT or HD animals at the indicated ages were sacrificed and 2–3 mm samples of the four brain regions of interest were collected, flash frozen and stored in liquid nitrogen until use. Samples were minced with scissors in 30 µL of buffer (HBSS, 20 mM EDTA, 10% DMSO) on ice. Next, 400 µL of buffer was added (200 µL for the HIP region, as it usually has the least cells). The mix was added on a 40 µm filter-top tube and centrifuged for 3 min at 150 × *g* and 4 °C. The cell concentration for each region was estimated by mixing 10 µL of each sample with 1 µL of 100x SYBR Gold (Invitrogen, Cat. no. S11494), pipetting 10 µL on a hemocytometer (Hausser Scientific, PA, USA) secured on a glass slide and counting cells at 5× magnification using an inverted LED fluorescence motorized microscope (Zeiss LSM 710 microscope, Carl Zeiss Microscopy, GmbH, Germany). Tissue samples were brought to 300,000 cells/mL in 25 µL with PBS and used for the Comet assay.

### Quantification of DSBs using the Comet assay

Neutral Comet assays were performed to quantify the amount of DSBs in brain regions (CBL and STR) or genotypes. Dispersed cells from tissue of three WT or HD animals were pooled and adjusted per genotype to 300,000 cells/mL A 25 µL aliquot of each sample was mixed with 250 µl molten low-melting agarose (R&D Systems, Cat no. 4250-050-02). 40 µL of this mixture was pipetted onto a 3-well FLARE slide (R&D Systems, Cat no. 3950-075-02) and spread with the pipette tip. For the Neutral Comet, slides were immersed in cool neutral buffer (0.1 M Tris, 0.5 M Sodium Acetate, pH 9) for 30 min, before electrophoresis was performed at 21 V for 45 min at 4 °C in 850 mL of neutral buffer. Slides were put in DNA Precipitation Solution (1 M Ammonium Acetate in 95% EtOH), then 70% EtOH for 30 min each. For Comet assays, slides were dried at 37 °C for 10–15 min. 100 µL 1x SYBR Gold (Invitrogen, Cat no. S11494) were placed onto each well for 30 min in the dark, before being rinsed with distilled water. Slides were dried completely at 37 °C, and fluorescence 5 × 5 tilescan (0.6 zoom) images of the comets were captured at 5× magnification using an inverted LED fluorescence motorized microscope (ZEN 2.1 SP3 FP3 (black) Zeiss LSM 710 microscope, Carl Zeiss Microscopy, GmbH, Germany). Comet images were analyzed using Trevigen comet analysis software (R&D Systems, MN, USA). We scored at least 100 cells per sample. We have calculated DSBs using tail length and tail moment with equivalent results^88^. The tail moments were used as the parameters for estimating DSB levels.

### Preparation and culturing of primary glia from brain regions

Intact brains were collected from newborn litters (P1-3) of C57BL/6J mice. Four to eight pups in each litter were used to isolate brain regions (cerebellum and striatum). The number of pups collected was necessary due to the small size of the embryonic brain regions and low yield of primary cells. One-two WT pregnant females were typically used to collect 4–8 embryonic WT pups and, due to smaller litter sizes, 3 (or more) pregnant HD females. The tissue samples were suspended in a solution of HBSS supplemented with 1mM L-glutamine, 1 mM sodium pyruvate and 1x Non-Essential Amino Acids and digested in 5 mL 0.025% Trypsin-EDTA (Gibco 25300056) for 20 min at 37 °C with gentle rocking. Tissue pieces were pelleted (5 min, 300 g, room temperature (RT)) and then gently triturated 20–30 times in pre-warmed astrocyte media (Neurobasal A base media (Thermo-Fisher #10888022), 10% FBS (JRS 43635), 2% B27 Supplement (Themo-Fisher #1504044), 25 mM glucose, 2 mM sodium pyruvate, 2 mM GlutaMax, 1x non-essential amino acids (Quality Biologicals 116-078-721EA), 1x antibiotic/antimycotic (Gibco #15240062) using a 5 mL pipet, to dissociate into cells. Each cell suspension was tested for mycoplasma and negative cultures were passaged 3 times to expand cell number. Each passage was cultured for 6–10 days (at 37 °C, 5% CO_2_) with media exchanges every 2–3 days. Astrocyte cell purity and homogeneity was established by immunofluorescent analysis using anti-GFAP antibody-Cy3 conjugate (Abcam ab49874).

### Transfections in primary glia cultures

Primary mouse glia was cultured in prewarmed DMEM high glucose medium supplemented with 20% fetal bovine serum, GlutaMax, non-essential amino acids, plasmocin, normocure, and penicillin/streptomycin. For transfection experiments, 0.05 million cells were seeded per well in 12-well plates and allowed to attach overnight. All cells were passages to achieve about 80% confluency. On the day of transfection, cell culture medium was changed to 1 ml OPTI-MEM, and the cells were transfected with 1 µg plasmid cocktails using lipofectamine 3000 (Thermofisher Scientific) according to the manufacturer’s protocol. The transfection medium was changed to cell culture medium at 4 h post transfection. At 24 h post transfection, the cells were washed with PBS, trypsinized, and filtered through a 40 µm strainer cap tubes for flow cytometry analyses.

### FM-HCR reporter vectors

All reporter plasmids used for FM-HCR were pMax vector-based. They were engineered with site-specific DNA lesions as previously described and interfere with expression of the fluorescent reporter^86–88,134^. The reporters checked for the presence of lesions using restriction analysis and functionally checked in knockout lines. Some of the published testing is provided in the source files. Correction of the lesion restores fluorescent intensity of the reporter. For NHEJ reporter, pMax BFP with a ScaI recognition site (BFP-ScaI)(Supplemental Fig. 6) was linearized with ScaI restriction to generate a blunt end that is resolved by NHEJ. For the NER reporter, pMax_mPlum was irradiated with UV-C light at 800 J/m^2^ and was validated by analytical digest with T4PDG enzyme. pMax_mOrange G:G reporter expresses morgange on removal of a common G-G mispaired base. The PspOMI HR reporter was described previously^134^. To generate the double strand break, the PspOMI mCherry was linearized with PspOMI restriction enzyme to create the overhangs that disrupt mCherry expression (Supplemental Fig. 6). In the HR assay, a non-fluorescent promoterless mCherry reporter was used as the donor plasmid, such that mCherry expression is restored only with HR. For BER, pMax_GFP_THF reporter measures APE1 activity for long-patch BER activity. Tetrahydrofuran is the stable analog of the abasic site, which serves as a lesion that can be processed by APE1^135^. All vectors were sequenced to confirm the correct nucleotides and the sequences of the reporter vectors submitted to GenBank are included in Source files. Features of the reporter sequences shown in Fig. 4B.

### The transfection and analysis of two FM-HCR reporter cocktails

FM-HCR measures DNA repair activity in cells using a set of five reporter plasmids, described above, each harboring specific lesions corresponding to one of the five major DNA repair pathways. Two cocktails are made. Cocktail 2 (cocktail 2 (+lesion)) comprising all five reporters harboring their pathway-specific lesions and are transfected together with a transfection control plasmid. The reporters in Cocktail 1 are the same five genes as in cocktail 2 but contain no lesion and retain full activity + the transfection control plasmid. Cocktail 1 and Cocktail 2 are transfected separately, but the intensities from both cocktails are normalized using the same transfection control. Because the lesion disrupts the coding sequence and its expression, recovery of reporter expression serves as a measure of pathway-specific lesion repair. All transfected cells were analyzed by flow cytometry (Attune NXT Flow cytometer, Thermofisher Scientific) at 24-h post transfection as previously published^86–88^. Repair activity is expressed as % reporter expression, calculated from the ratio from the normalized fluorescence intensity of the damaged reporters (in cocktail 2) and fully active undamaged reporters (in cocktail 1). The FM-HCR assays have been validated in other cells lines, e.g., human lymphoblastoid cell lines^86,87,134^, and plasmid activities have been validated against in most other DNA repair assay including knockout lines (select published examples are shown in source files for Fig. 4).

### XJB-5-131 treatment

XJB-5-131 (gift form P Wipf, University of Pittsburgh) was used as previously described^7,65,88^. Lyophilized, powdered XJB-5-131 was reconstituted in DMSO at a concentration of 1 μg/μL. These samples were aliquoted and kept at −80 °C. On the day of injection, the XJB-5-131 solution was mixed with 0.2 μm filtered and pre-warmed PBS (100 °C) to reach a final concentration of 2 mg/kg mouse body weight in 200 μL solution. This was heated for 10 s, and the solution (200 μL) was injected, within 30 min of preparation, by intraperitoneal injection (IP). Administration started at 60 wks of age and continued three times per week for 30 wks. Vehicle treatments were identical except that XJB-5-131 was replaced by filtered PBS.

### Statistical analysis

Statistical analysis is reported as appropriate in each Figure. The statistical treatment of biological n for congenic strains is presented in the animal breeding methods. For single cell analysis, results from the total number of tested animals or cells are plotted together in box and whisker plots, where each point in the plot is an individual measurement. The box represents 50% of the data points and the remaining 50% are in the whiskers, distributed as the 25% maximal values above the box and 25% minimal values below the box. The line indicates the median value. The protein expression, cell radiation kinetics, DSBs in zQ175 analysis, the graphical representations were expressed as Mean ± SD. For all results, the significance was determined using a one-way ANOVA using GraphPad PRISM version 9.5.1 for Mac, GraphPad Software, San Diego, California USA. A *p* < 0.05 was considered as statistically significant using ANOVA models with post-*hoc* multiple comparison tests by GraphPad PRISM (GraphPad Software, LLC).

### Reporting summary

Further information on research design is available in the Nature Portfolio Reporting Summary linked to this article.

## Supplementary information

Supplementary Information

Reporting Summary

Transparent Peer Review file

## Source data

Source Data

## Data Availability

There are no restrictions on the availability of these data. Source data and uncropped gels are provided for all Main text figures and Supplementary figures. Source data are provided with this paper.
