# Supplementary material for: Double strand breaks drive toxicity in a Huntington’s disease mouse model with or without somatic expansion
Source: Nat Commun. 2026 May 6;17:6381. doi: 10.1038/s41467-026-72382-z (PMC13377174; doi:10.1038/s41467-026-72382-z)
Supplement: Supplementary file 1 — Supplementary Information [file 41467_2026_72382_MOESM1_ESM.pdf]

Double strand breaks drive toxicity in Huntington's disease mice model with or without somatic expansion.

Aris A Polyzos[1], Ana Cheong[2], Jung Hyun Yoo[1], Lana Blagec[1], Zachary D Nagel[2] and Cynthia T McMurray[1*]

[1]Division of Molecular Biophysics and Integrated Bioimaging,

Lawrence Berkeley National Laboratory,

Berkeley, CA USA 94720.

[2]Department of Environmental Health

John B Little Centre of Radiation Sciences

Harvard T.H. Chan School of Public Health

Boston, MA USA 02115.

*To whom correspondence should be addressed:

Tel: (510) 486-6526; Fax: (510) 486-6880

Email: ctmcmurray@lbl.gov or aapolyzos@lbl.gov

Genescans
(+ = repeat gain from tail midpoint)

ID: 668

HD150KI(-/+) 668
Tail, 4wks

HD150KI(-/+) 668
Brain, 30 wks

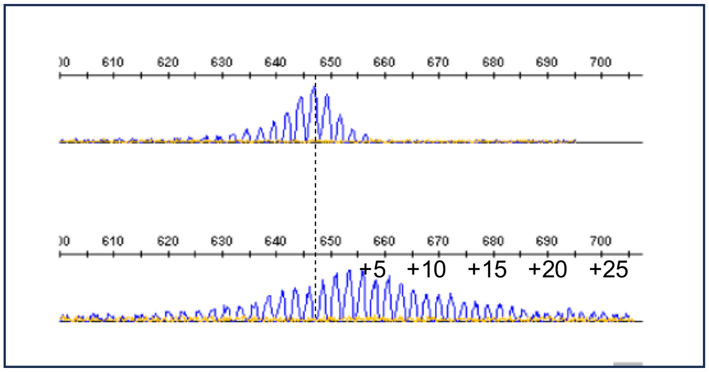

ID: 621

HD150KI(-/+) 621
Tail, 4wks

HD150KI(-/+) 621
Brain, 30 wks

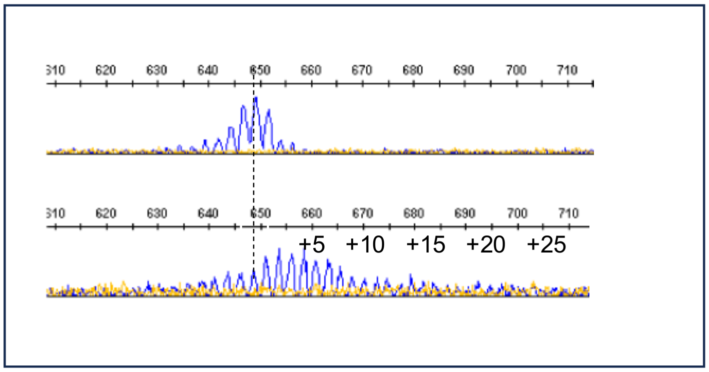

ID: 162

HD150KI(-/+) 162
Tail, 4wks

HD150KI(-/+) 162
Brain, 30 wks

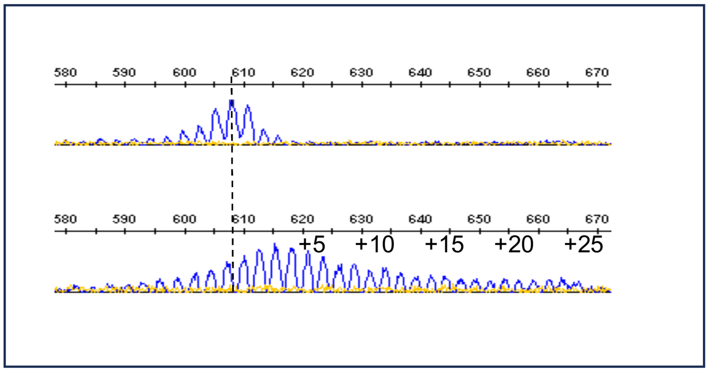

ID: 163

HD150 (-/+) 163
Tail, 4wks

HD150 (-/+) 163
Brain, 30wks

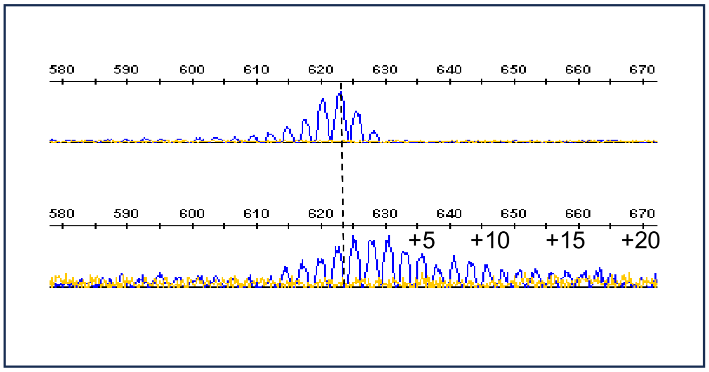

Supplemental Figure 1

Supplemental Fig. 1. Somatic expansion occurs in the brains of *HdhQ(150/150)* mice.

Purified DNA from tail or brain-derived tissue was assessed for quality by PCR. High-quality DNA was submitted to TransnetYX for CAG sizing by high-resolution capillary electrophoresis. Representative Genescan traces illustrating somatic expansion are shown. Paired scans compare tail DNA at 4 weeks and cortical brain tissue at 30 weeks from four *HdhQ(+/150)* animals (IDs: 668, 621, 162, and 163). The dotted line denotes the midpoint of the tail allele prior to the onset of somatic expansion and represents the inherited CAG repeat length. Each peak corresponds to a single CAG repeat. The (+ numbers) in the plot to the right of the Genescan indicate the increase in somatic CAG repeat length at 30 wks relative to the tail midpoint at 4 wks.

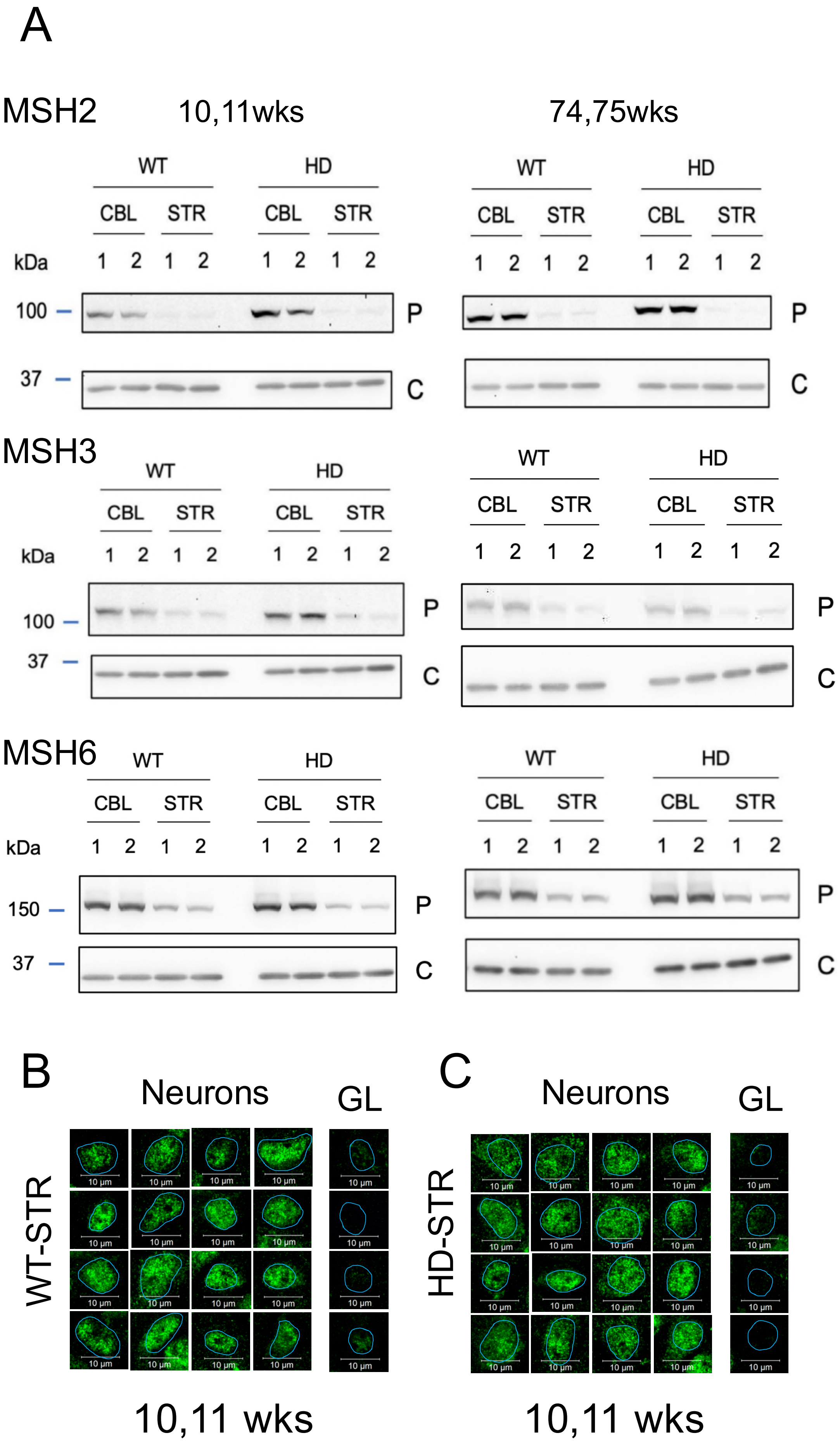

Supplemental Figure 2

Supplemental Fig. 2. *HdhQ(150/150)* and WT mice express the MMR recognition machinery. (A) Western blots for MSH2, MSH3, and MSH6 resolved by SDS–PAGE, corresponding to the quantified IF intensities as shown in Fig. 1C. The same as Fig. 1B, except that MSH2, MSH3, and MSH6, are visualized together. Brain extracts from affected striatum (STR) or resistant cerebellum (CBL) were prepared from congenic, genetically identical C57BL/6J (WT) and *HdhQ(150/150)* (HD) mice at 10–11 weeks and 74–75 weeks of age. Extracts from n=2 animals per genotype (labeled 1 and 2) were resolved side by side by SDS-PAGE, transferred to membranes, and probed with antibodies against MSH2, MSH3, or MSH6 (P), or GAPDH (C). Molecular weight markers (kDa) are shown to the left. IF band intensities in arbitrary units were quantified by phosphorimaging. The bars reflect the average IF intensity of the two sample values, which are shown as circles. Antibodies used are listed in Supplementary Table 1. (B) Representative images of MSH3 immunostaining in NeuN(+) striatal neurons (Neurons) from brain sections of WT (B) and *HdhQ(150/150)* (C) mice at 10-11 weeks. Each magnified neuron is positive for both MSH3 and NeuN+ throughout the brain section. N=16 magnified images were randomly selected from n=4 tissue fields per genotype. Shown only is the green channel for MSH3. For both WT and HD animals, n=4 magnified NeuN(–) glial cells (GL) are shown, which had the same staining pattern in all tissue fields. Blue outlines denote nuclei defined by DAPI. Scale bar, 10 μm. Full uncropped gels are provided in the Source Files. Antibodies are listed in Supplementary Table 1.

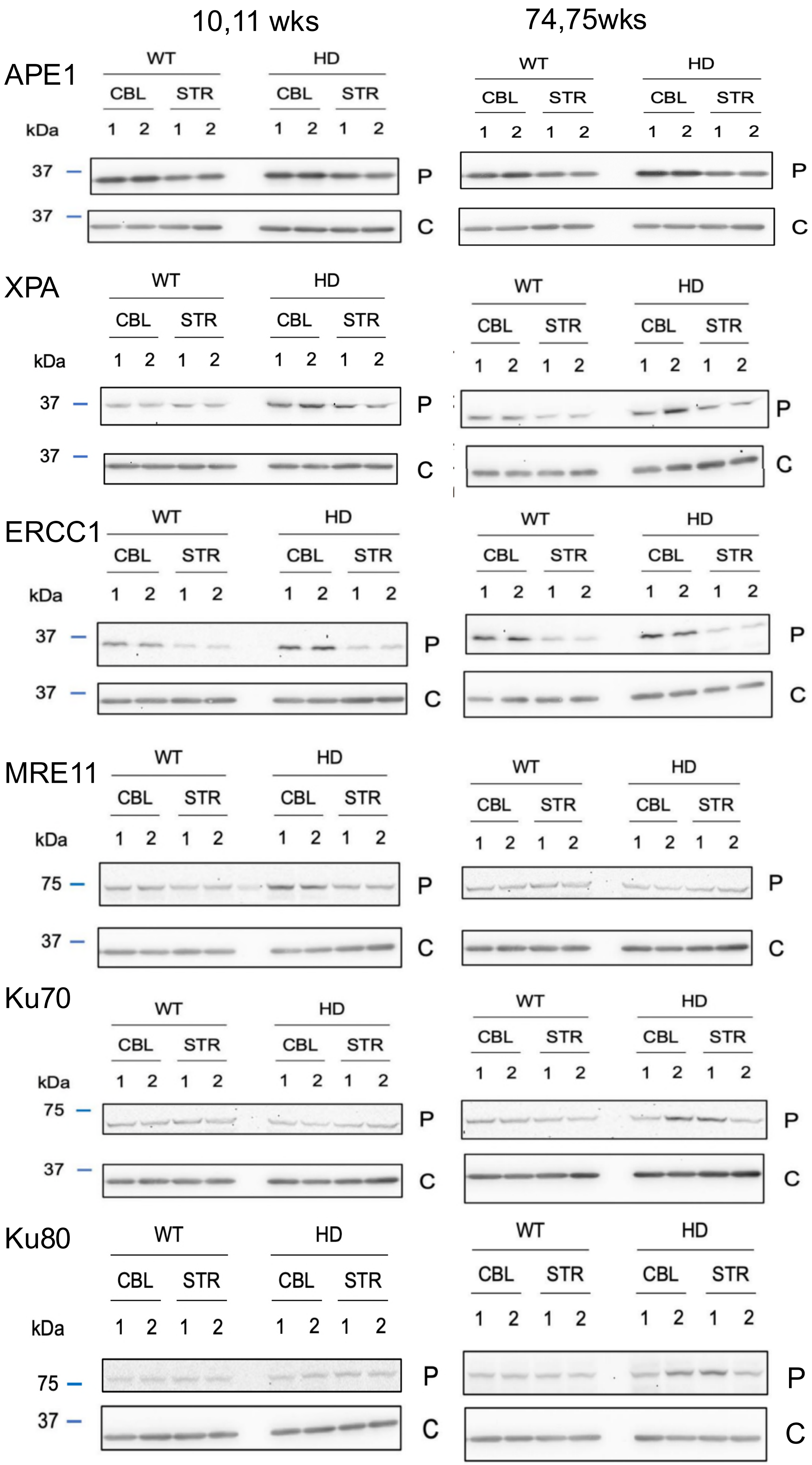

Supplemental Figure 3

Supplemental Fig. 3. WT and *HdhQ(150/150*) mice express the machinery to carry out DNA repair.

The SDS–PAGE resolved proteins in WT and HD animals, corresponding to IF intensity plots in Fig. 3B for the multiple DNA pathways shown in Fig. 3A. The gels and controls for the five DNA repair pathways are the same as those described for the MSH3 antibodies in Fig. 1 and Supplementary Fig. 2. Protein extracts from n= 2 animals were resolved on n-12 replicate blots, which were probed with antibodies to one of six proteins measured in 10-11 wks (left) or 74–75-wk animals (right) of both genotypes. As in Supplementary Fig. 2, two protein samples were loaded side by side on the same gel and are indicated by numbers 1 and 2. Representative pathway proteins include apurinic/apyrimidinic (AP) endonuclease (APE1); xeroderma pigmentosum group A protein (XPA), xeroderma pigmentosum group F protein (XPF), and excision repair cross-complementation group 1 (ERCC1); meiotic recombination 11 homolog 1 (MRE11); X-ray repair cross-complementing protein 6 (Ku70); and X-ray repair cross-complementing protein 5 (Ku80). Full uncropped gels are provided in the Source Files. Protein antibodies are listed in Supplementary Table 1.

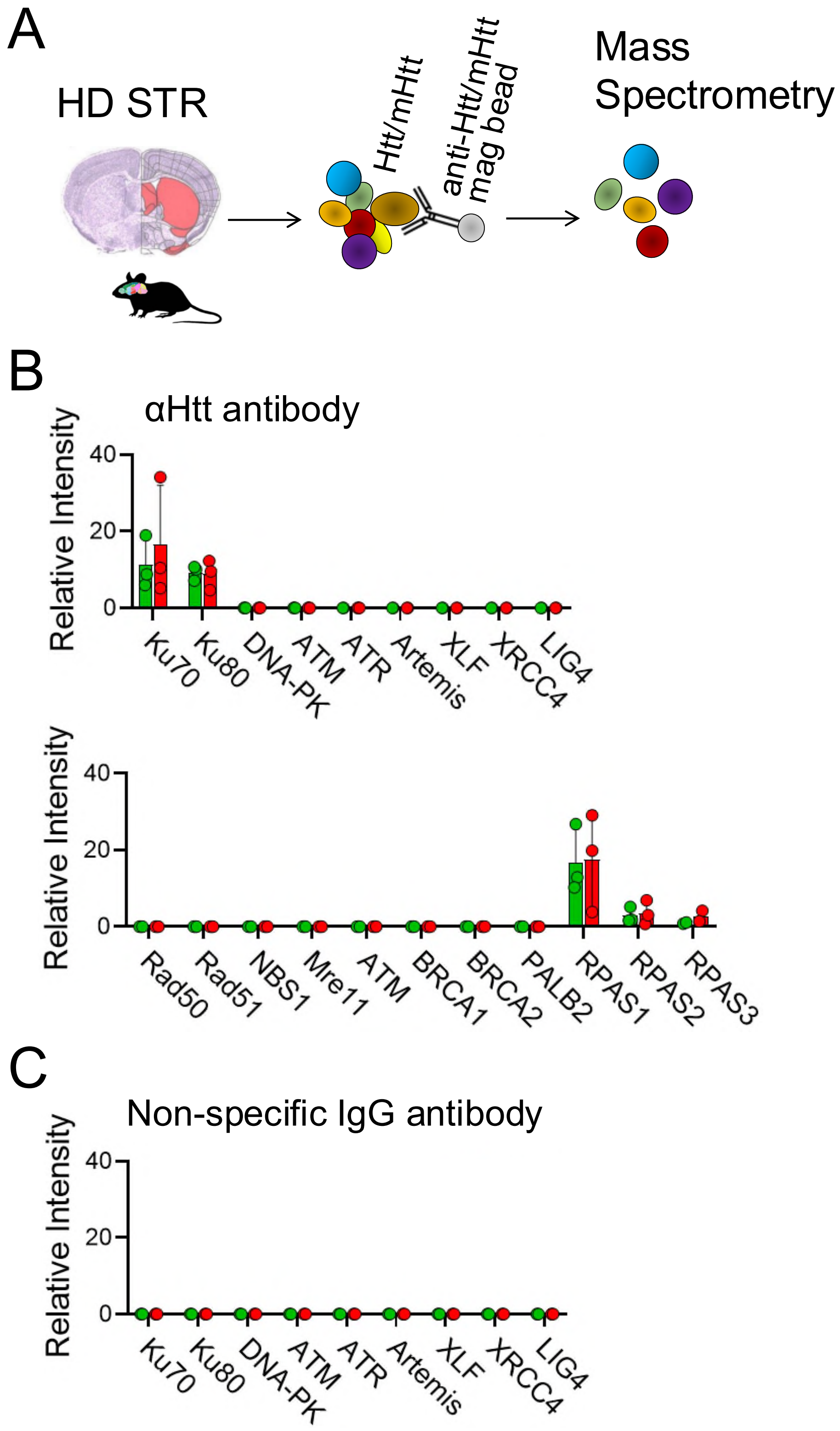

Supplemental Fig. 4

Supplemental Fig. 4. Htt and mhtt interact strongly with Ku70/Ku80 of the NHEJ pathway in STR from WT and *HdhQ(150/150*) mice.

Mass spectrometry (MS) analysis was performed by the Mass Spectrometry Facility at the University of California, Davis (UC Davis). Methods were the same as those described for Fig. 3C, D of the main text, except that samples are striatal extracts from brain tissue of WT or *HdhQ(150/150)* mice per independent experiment. (A) A schematic diagram of immunoprecipitation/mass spectrometry (IP–MS) analysis illustrates the identification of DNA repair proteins interacting with an htt antibody in STR of n=3 WT and n=3 *HdhQ(150/150)* mice. MS analysis was performed in triplicate. Source Data provided. STR is illustrated in red in the Allen brain atlas map[135] to the left. **(B)** Results of the IP–MS analysis in **(A)** for the STR of WT (green) and *HdhQ(150/150)* (red) mice, as described in Fig. 3C,D. The results of n=3 replicates are plotted as mean peptide level ± standard deviation (SD), where error bars are standard deviation (SD). As observed in cell extracts (Fig. 3D), the major pull-down products from brain tissue include Ku70 and Ku80 of the NHEJ pathway and replication protein A (RPA) single-strand binding proteins (right). (C) Same as B substituting IgG antibody for the htt antibody. Peptides in the samples A and B were separately matched to the resulting library using Spectronaut default settings. Briefly, “trypsin/P specific” was selected to allow up to two missed cleavages. Fixed modifications were set to cysteine carbamidomethylation, and variable modifications included peptide N-terminal acetylation and methionine oxidation. For DIA search parameters, PSM and protein group decoy false discovery rates were set to 1%. Protein-level intensities were summarized using the MaxLFQ algorithm. The integrated area of eluted peptides was used to compare captured proteins from WT (green) and *HdhQ(150/150)* (red) samples, which were prepared separately but analyzed in parallel on the same day.

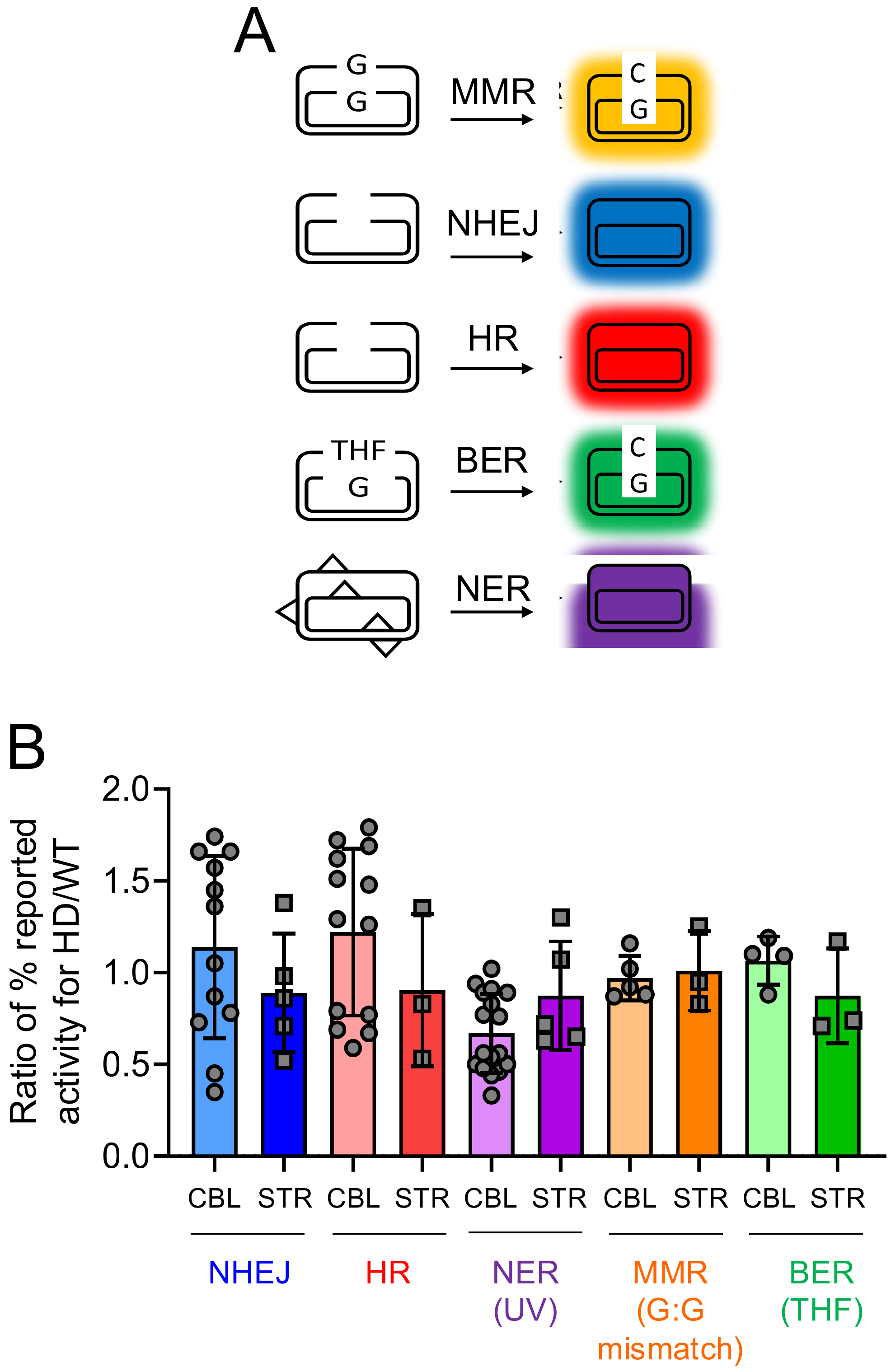

Supplemental Figure 5

Supplemental Fig. 5. HD and WT glial cells have similar DNA repair activities.

The methods and plasmids used for FM-HCR are described in detail in Fig. 4A,B of the main text. (A) Brief summary of the color-coded reporter plasmids for HR, NHEJ, MMR, NER, and BER used in (B). FM-HCR measures DNA repair activity using a set of reporter plasmids, each harboring specific lesions corresponding to one of the five major DNA repair pathways, as described in Fig. 3A. Because the lesion disrupts the coding sequence and reporter expression, recovery of fluorescence intensity of reporter expression serves as a measure of pathway-specific lesion repair. (B) The ratio of % reporter expression in HD relative to WT cells is plotted, each determined from the average % reporter expression for HD and WT glia in the independent transfections shown in Fig. 4C. The error bars are standard deviation. Raw data are reported in the Source data files.

A

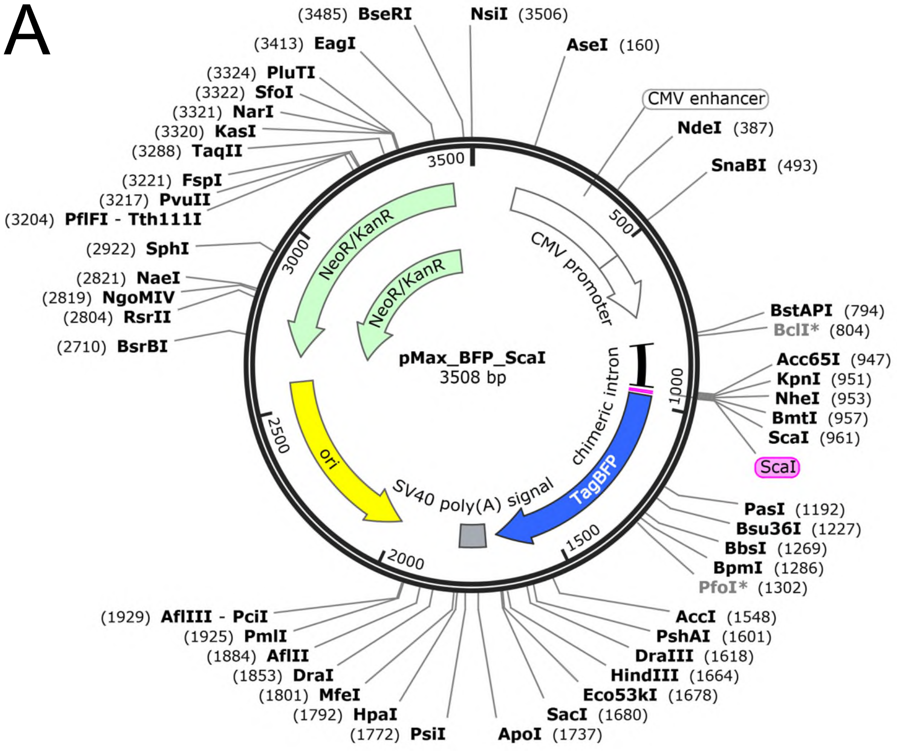

SCA site: 5′-…TAGC**AGT|ACT**GCTG…-3′
3′-…ATCG**TCA|TGA**CGAC…-5′

B

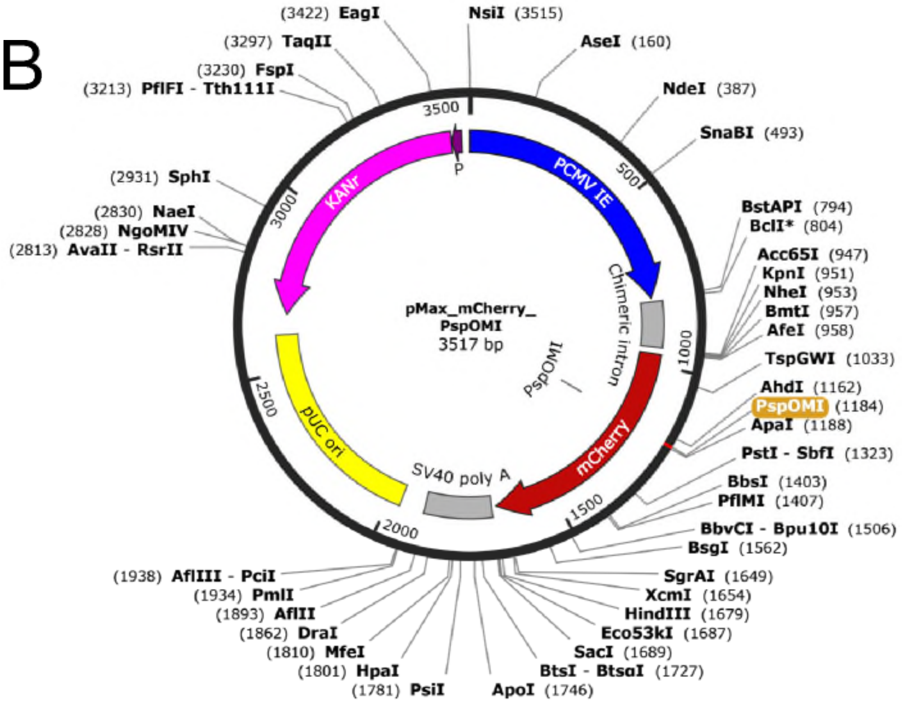

PspOMI 5′-…GTAC**G|GGCCC**AAGG…-3′
3′-…CATG**CCCGG|G**TTCC…-5′

Supplemental Fig. 6

Supplemental Fig. 6. Schematic diagrams of vector maps for NHEJ and HR reporters used in the FM-HCR.

(A) Vector maps for the pMax_BFP_ScaI reporter for NHEJ. The ScaI recognition site (highlighted in pink) is upstream of the 5' end of BFP fluorescent gene (TagBFP). Enzymatic digestion with ScaI yields a blunt end double strand break that is recognized and repaired by NHEJ. The ScaI restriction sequence is shown below the vector map; "|" refers to the cleavage site. (B) The vector map of pMax_mCherry_PspOMI HR reporter. A PspOMI recognition site (highlighted in orange) is located in the mCherry fluorescent gene sequence (in red). Enzymatic digestion with PspOMI yields an overhang DSB that is resolved by HR and not NHEJ. The PspOMI restriction sequence is shown in the vector map; "|" refers to the cleavage site. Sequences for all reporters have been submitted to GenBank (see Source Files).

A

Glia-CBL (24hrs post irradiation)

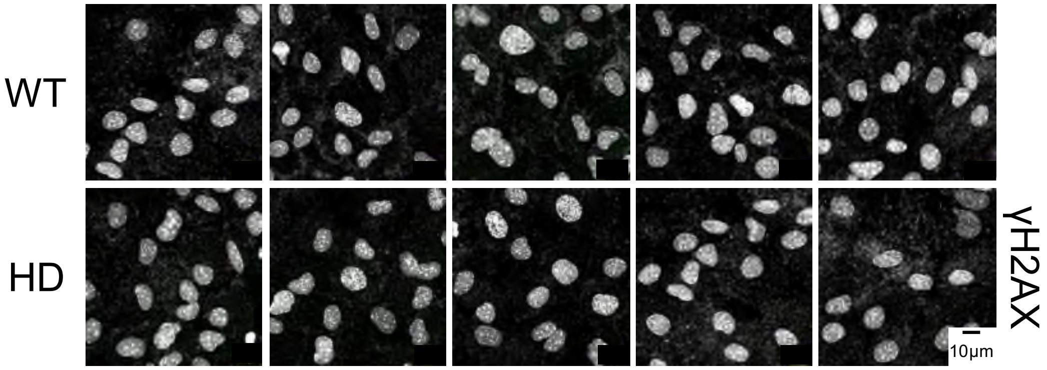

B

Glia-STR (24hrs post irradiation)

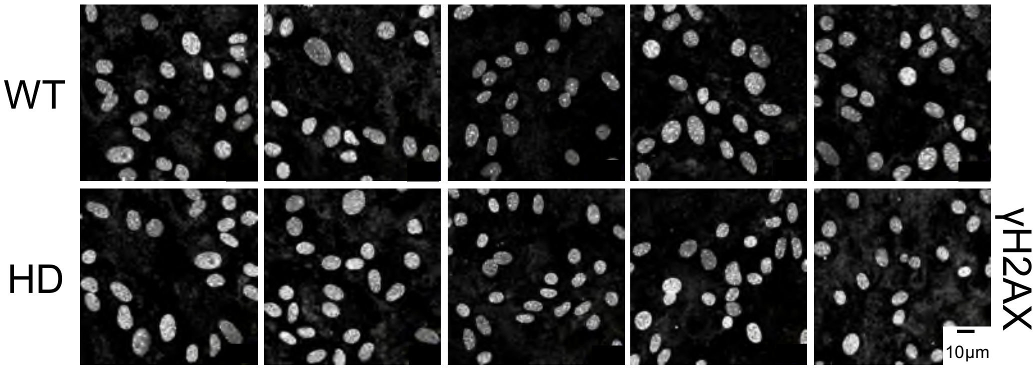

C

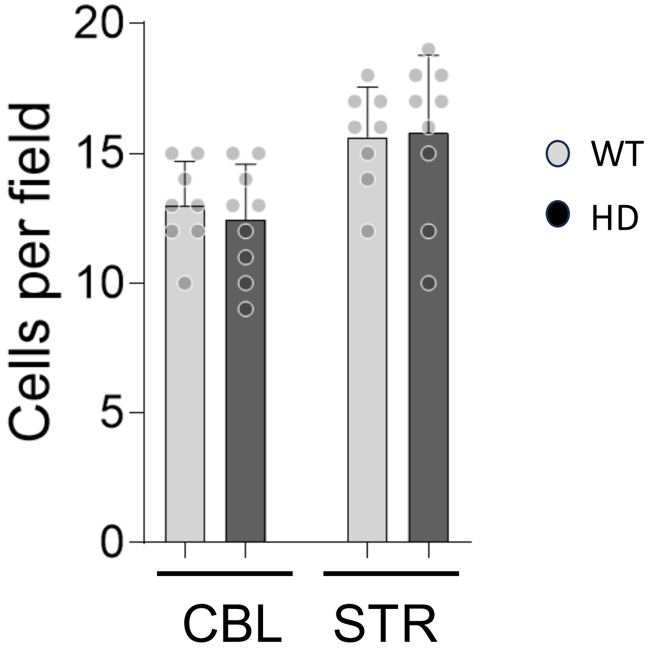

Supplemental Fig. 7

Supplemental Fig. 7. Radiation exposure does not kill brain cells.

(A,B) Purified glia from CBL **(A)** or STR **(B)** were isolated from n = 8 dissected embryos obtained from pregnant WT or *HdhQ(150/150)* females and exposed to 2 Gy irradiation. Shown are images from *n* = 5 random fields of γH2AX stained cells acquired 24 hours post-irradiation in the CBL (A) and the STR (B) for WT (top panels) and *HdhQ(150/150)* mice (bottom panels). Raw data included in source files. Cells developed γH2AX foci but remained intact and exhibited normal morphology. Scale bar, 10 μm. **(C)** Quantified cell numbers per field for STR (right) and CBL (left) cultures in (A): WT (light gray) and HD (dark gray). Data are presented as mean ± SD. No statistically significant differences in cell numbers were observed between genotypes in either brain region.

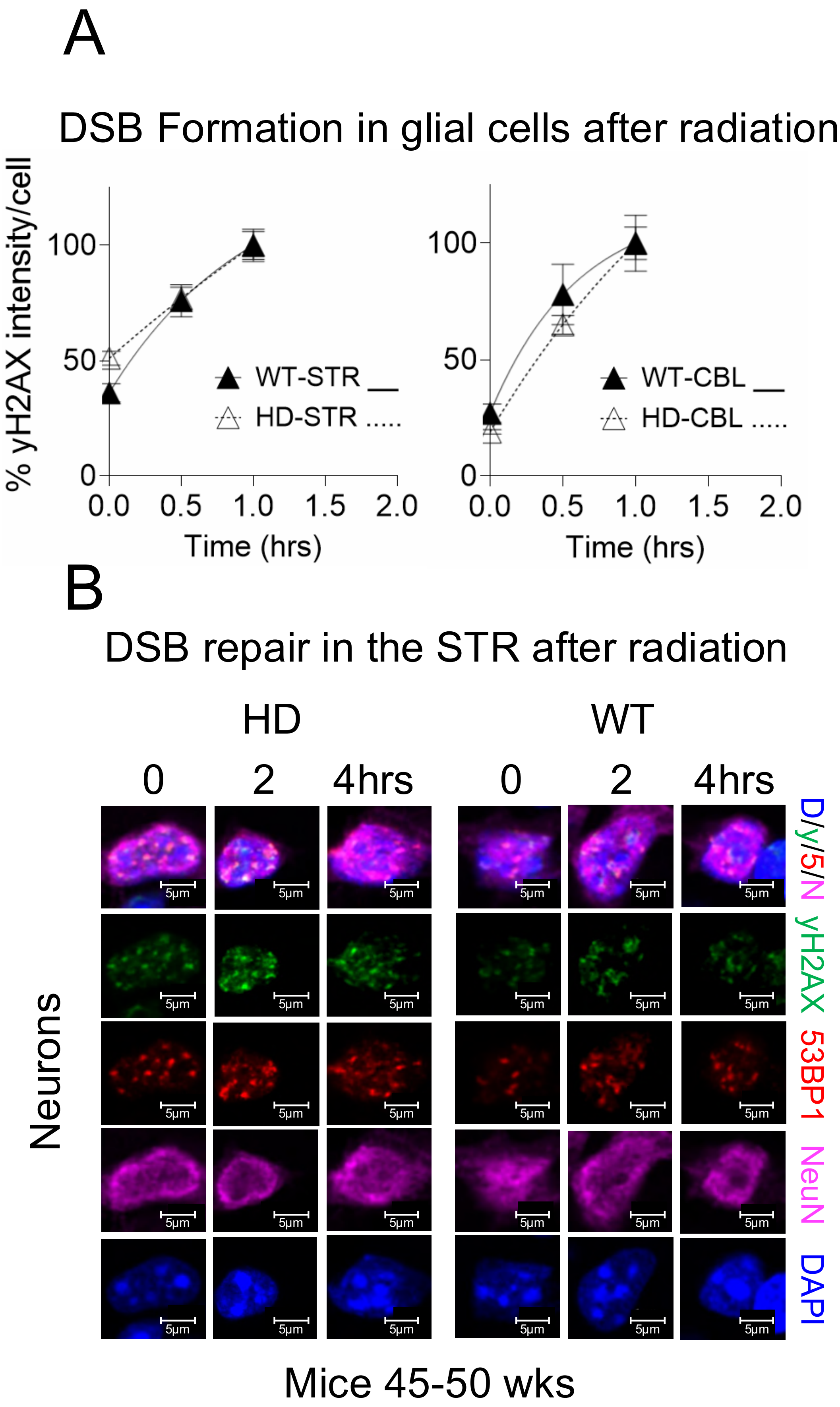

Supplemental Figure 8

Supplemental Fig. 8. γH2AX foci are removed slowly in *HdhQ(150/150)* animals relative to WT animals post irradiation.

(A) Rate of γH2AX foci formation over time in irradiated glial cultures from Supplemental Fig. 7. Glial cultures from WT and *HdhQ(150/150)* animals were evaluated immediately after exposure to 2Gy irradiation (0hrs), and at 0.5hrs and 1h post-irradiation. WT is shown as solid triangles and solid lines; HD is shown as open triangles and dotted lines. The data for γH2AX foci formation at 0, 0.5 and 1hr time points were analyzed using a polynomial fit to derive the DSB formation rate. γH2AX foci per cell increased to a similar extent and at comparable rates post-irradiation in cultures derived from the STR (left) and CBL (right) of both genotypes. In the STR, WT is 63.4/hr and the HD rate is 49/hr. R^2 is 0.7. In the CBL, the WT rate is 72/hr and the HD rate is 81/hr. R^2 is 0.8. **(B)** Magnified images of γH2AX foci formation in striatal neurons from brain tissue sections of 45–50 week old WT (right) or *HdhQ(150/150)* (left) mice irradiated with 5Gy, corresponding to the plots in Fig. 5F,G. The same staining pattern was observed in n = 4 fields in the tissue section. Scale bar is 5.0µm. DSBs were measured by γH2AX or 53BP1 immunofluorescence at 0, 2, and 4 hours post-irradiation. Shown in columns are magnified NeuN(+) neurons from tissues at the indicated post-irradiation time. Tissues were co-stained with antibodies against γH2AX (green), 53BP1 (red), NeuN (purple), and DAPI (blue), arranged as individual color channel (panels 2-4), or as a merged overlay image of all four markers (DAPI, γH2AX, 53BP1, NeuN) (D/y/5/N, Panel 1). γH2AX immunofluorescence intensity remained elevated in neurons from *HdhQ(150/150)* mice (left) relative to WT mice (right) at 2 or 4 hours post irradiation, consistent with a suppression of DSBR in the *HdhQ(150/150)* mice, as measured in Fig. 5 F,G of the main text.

STR, 4 hours post-irradiation
in 45-50wks animals

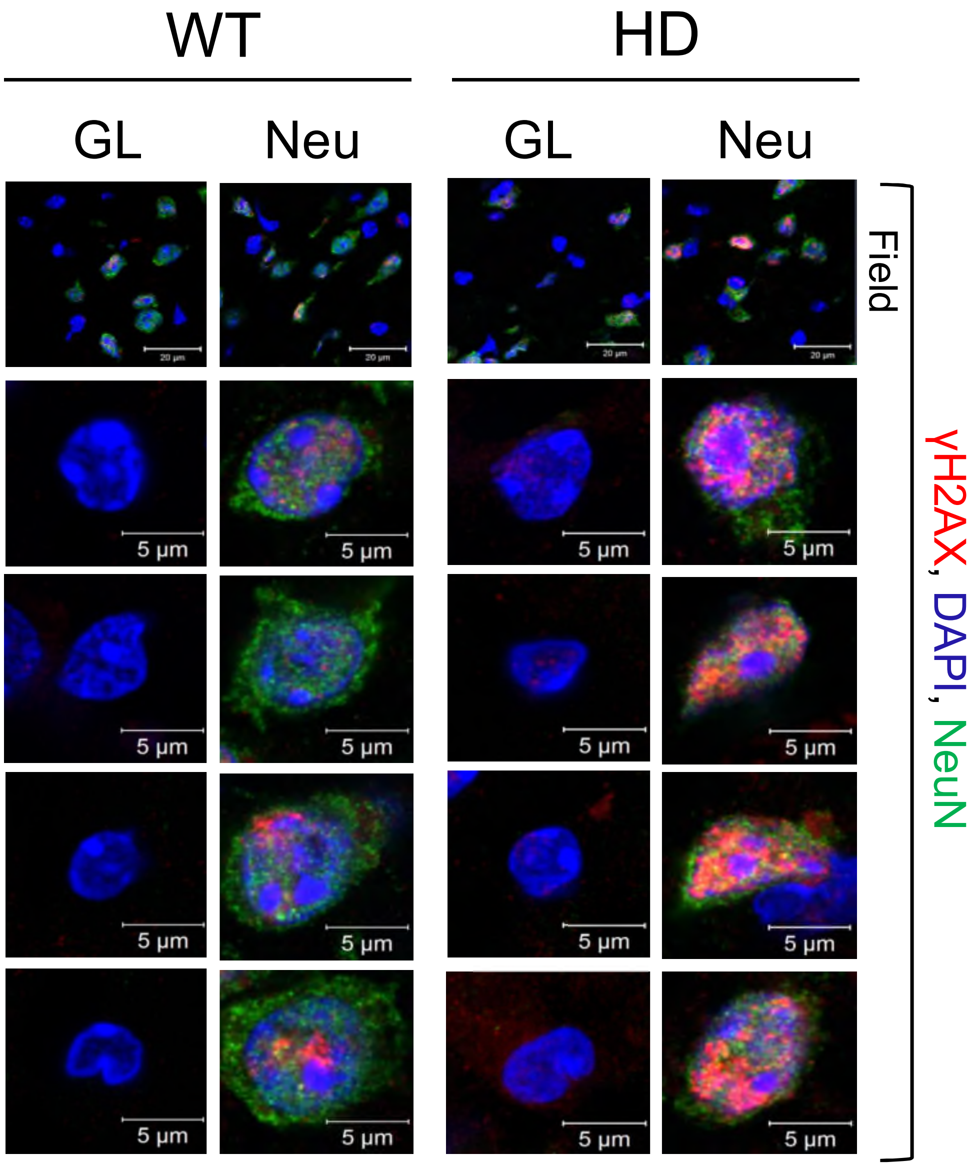

Supplemental Figure 9

Supplemental Fig. 9. Neurons are preferentially sensitive to DSBs in HD brains.

(Top panel) Shown is one of the n=4 randomly selected fields in the striatal tissue sections from 45-50 wk animals, corresponding to Fig. 5F,G in the main text. WT (left) and HD (right) tissue at 4 hrs post-irradiation with 5Gy. The field of cells in the brain tissue is co-stained with γH2AX (red), NeuN (green), and DAPI (blue), and presented as overlay images to identify DSBs in NeuN+ neurons (right)(Neu) and NeuN(-) glia (GL) (left) for WT (left) or HD (right) animals. The scale bar is 5µm. n = 4 Neu or n = 4 GL cells are magnified. Scale bar is 5µm. Each of the n = 4 cells is an overlay image of γH2AX (red), NeuN (green), and DAPI (blue) staining. DSBs were detected by γH2AX staining (red), which is weak in GL and preferentially observed in cells that co-stain with NeuN (green) in both genotypes. γH2AX staining (red) is higher in HD cells relative to WT cells, consistent with DSBR inhibition in HD cells.

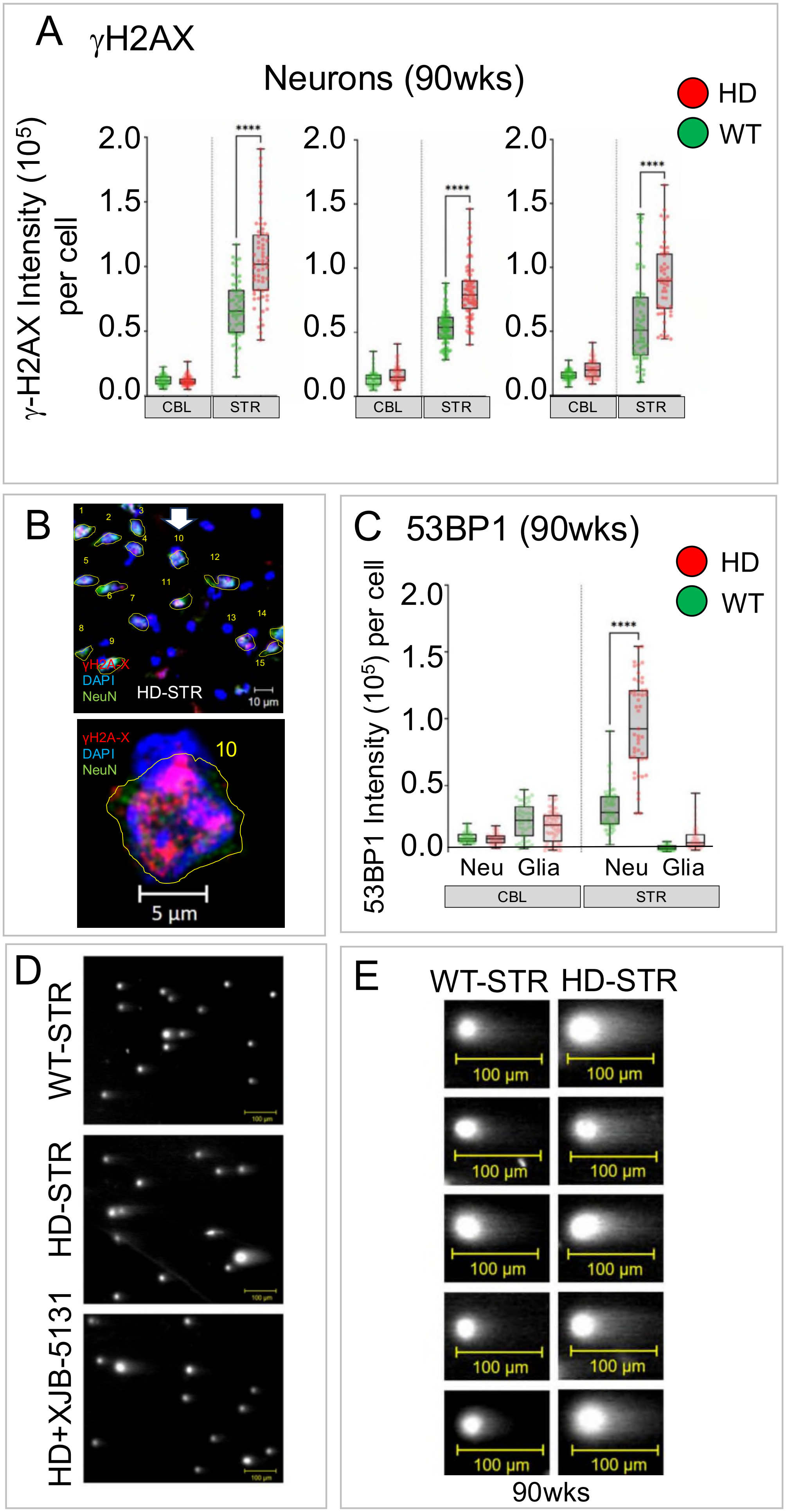

Supplemental Figure 10

Supplemental Fig 10. DSBs increase with age in *HdhQ(150/150)* relative to WT neurons. (A) Reproducibility of γH2AX single-cell quantification in the CBL and STR from *n* = 3 independent WT or HD animals aged 70–90 weeks. Brain tissue from n = 3 WT or HD animals was stained with γH2AX, with a similar pattern in n = 4 tissue field in the section. Roughly 50 γH2AX(+) cells in the CBL and STR were quantified per genotype. WT is shown in green and HD in red. γH2AX immunofluorescence (IF) intensities were pooled and plotted as box-and-whisker plots, where each point represents an individual γH2AX-stained cell. Statistical significance in the mean DSB levels between in WT or HD animals in each brain region was determined by one-way ANOVA (****P = 0.0001). (B) Examples of method for digital detection of γH2AX foci in NeuN(+) neurons and NeuN(–) glial cells. Tissue sections were stained with γH2AX (red), NeuN (green), and DAPI (blue) to distinguish neurons and glia. Representative fields from the STR of an HD animal aged 70–90 wks were selected by image analysis (Fiji). Cells are outlined in yellow and numbered in the top panel. The white arrow indicates NeuN(+) neuron #10, which is magnified in the lower panel and co-stains with γH2AX. Scale bar, 5 µm. **(C)** Same analysis as in **(A)** using the 53BP1 DSB marker**. (D)** Examples of comet analysis. Dispersed brain cells from WT (Vh), HD (Vh), or HD + XJB-5-131 animals at 90 weeks were resolved on individual neutral agarose gels (quantified in Fig. 6j,K and in Fig. 8G for XJB-5-131 in the main text). Broken DNA appears as slowly migrating material trailing behind genomic DNA, forming a comet tail. Scale bar, 100 µm. **(E)** Magnified images of n = 5 comet tails from WT (Vh) and HD (Vh) samples shown from the gel images in **(D).** Scale bar, 100 µm.

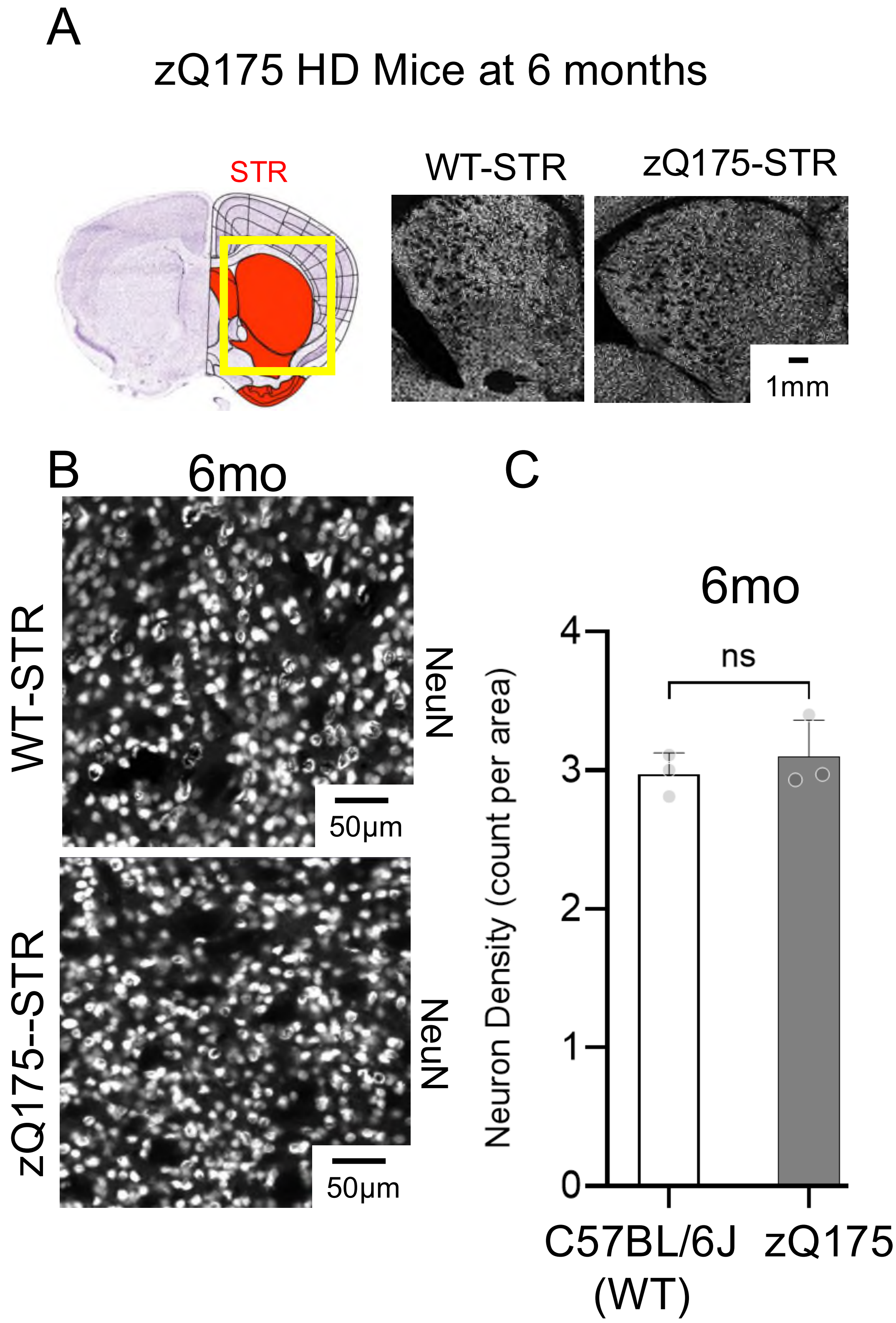

Supplemental Figure 11

Supplemental Fig. 11: No loss of neurons in *zQ175 or zQ175/MSH3(-/-)* animals by 6 months.

(A) (Left) Allen Brain Atlas[135] reference section showing the position of the striatum (STR, red) and the tissue used for immunofluorescence (IF) measurements (yellow box). **(Right)** Grayscale images from tissue sections of WT and zQ175 mice at 6 months of age from the two genotypes evaluated in Fig. 7 of the main text. **(B)** Magnified images from **(A)** stained with an antibody against the neuronal marker NeuN. Scale bar, 50 µm. **(C)** Quantification of neuronal density in striatal tissue sections from WT (left, white box) and *zQ175* (right, gray box) mice at 6 months of age. The entire STR was imaged using ImageJ, and neuronal number per unit area was determined from NeuN staining intensity. NeuN signal intensity was normalized to tile area to calculate neuronal density. Data were obtained from $n = 5$ random fields across n = 3 tissue sections from the STR of $n = 3$ animals per genotype (WT vs *zQ175*). No statistically significant differences were observed in neuronal counts (ns).

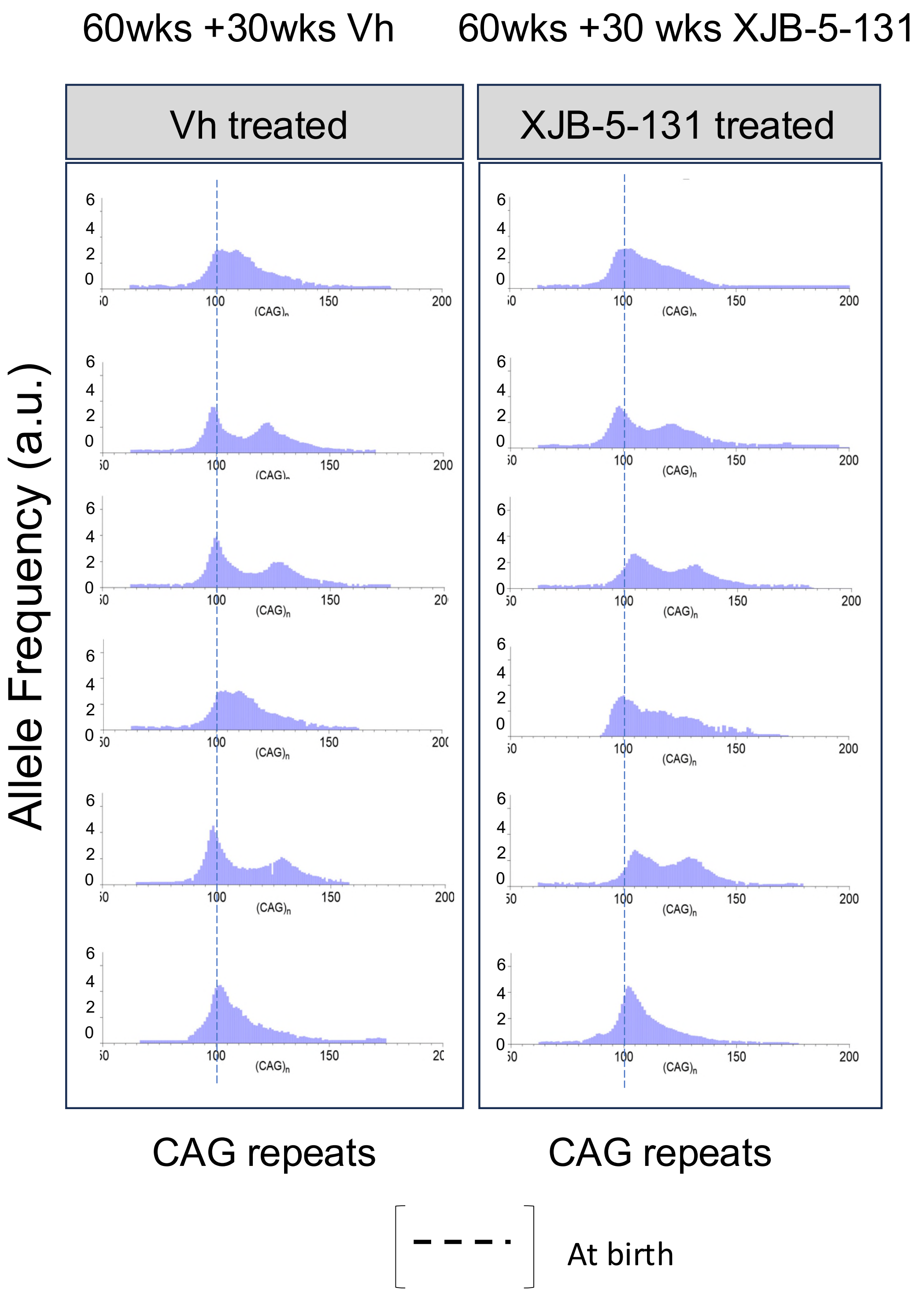

Supplemental Figure 12

Supplemental Fig. 12. XJB-5-131 treatment of *HdhQ(150/150)* mice has minimal effects on somatic expansion. Examples of Genescan traces for CAG repeat tract length in tissues from the saline vehicle (Vh)- or XJB-5-131- treated animals at 90wks (60wks aging + 30 weeks treatment) from Fig. 8D. Dotted line indicates the CAG tract in tail at birth. No significant differences in CAG tract lengths were obvious after XJB-5-131 treatment.

Comet, 90wks in STR

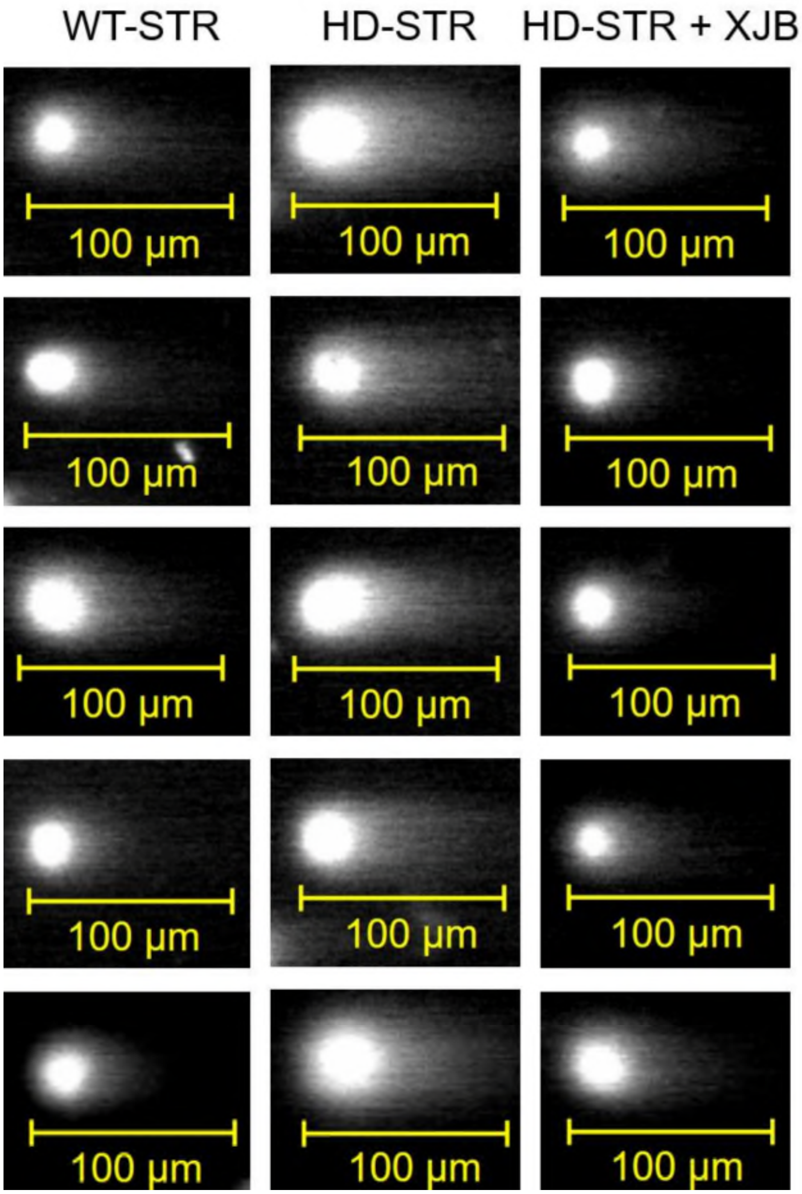

WT-STR , HD-STR = Saline vehicle (@90wks)
HD-STR +XJB = XJB (in saline) (@90wks)

Supplemental Figure 13

Supplemental Fig. 13. Broken DNA in comet tails in the STR of *HdhQ(150/150*) mice is reversible by XJB-5-131 treatment.

Magnified images of comet tails from the STR of 90-week vehicle (Vh)- and XJB-5-131–treated HD animals relative to WT (Vh) mice (from Fig. 8G). Dispersed brain cells from 90-week-old animals were collected for direct detection of DNA strand breaks using neutral comet assays (gels shown in Supplementary Fig. 10D). Approximately 100 comets per animal were quantified from dispersed brain cells from *n* = 3 animals per treatment group: WT (Vh) (light gray), HD (Vh) (dark gray), and HD + XJB-5-131 (blue), as indicated. Consistent with the DSB antibody markers, comet tail length increased in Vh-treated HD mice relative to Vh-treated WT mice and was reduced by XJB-5-131 treatment in HD animals.

Supplementary Table 1: Key Resources.

Reagent or Resource		Source	Catalogue ID
**Antibody**			
Mouse anti-NeuN alexafluor-488 conjugate	1:500	EMD Millipore	MAB377X
Rabbit anti-NeuN alexafluor-647 conjugate	1 :1,000	Abcam	190565
Mouse anti-GFAP Cy3 conjugate	1:500	Abcam	ab49874
Mouse anti-APE1	1:500	Novus	13B8E5C2
Mouse anti-Ku80		Santa Cruz	515736
Mouse anti-ERCC1		Santa Cruz	17809
Rabbit anti-MSH2		Abcam	ab92471
Mouse anti-MSH3		EMD Millipore	MABE324
Rabbit anti-MSH6		Abcam	Ab92471
Rabbit anti-XPA		AbClonal	A1626
Rabbit anti-MRE11		Novus	NB100-142
Mouse anti-yH2AX	1:400 – 1:1,000	ThermoFisher	MA1-2022
Rabbit anti-53BP1	1:1,000	Bethyl	A300-237A
Donkey anti-Mouse alexafluor-488 conjugate		Jackson Immunores.	715-545-150
Goat anti-Mouse alexafluor-568 conjugate		Invitrogen	A21124
Donkey anti-Rabbit alexafluor-488 conjugate		Jackson Immunores.	711-545-152
Goat anti-Rabbit alexafluor-555 conjugate		Invitrogen	A32732
Goat anti-Mouse alexafluor-488+ conjugate	1:1,000	Invitrogen	A48286
Goat anti-Rabbit alexafluor-555+ conjugate	1:1,000	Invitrogen	A48283
**Chemicals, peptides, and recombinant proteins**			
Fc Receptor Block		Innovex	NB309
Background Buster		Innovex	NB306
Tissue-Tek O.C.T. Compound		Sakura	4583
TrueBlack		Biotium	23007
Monarch RNase A		New England Biolabs	T3018L
ImmuMount		Epredia	9990402
Nuclease P1		New England Biolabs	M0660
Quick Calf Alkaline Phosphatase		New England Biolabs	M0525
CMV-cDNA26Q		constructed in house	
CMV-cDNA51Q		constructed in house	

T-PER Tissue Protein Extraction Reagent		Thermo Scientific	78510
Halt protease inhibitor cocktail		Thermo Scientific	78420
Pierce 660 Protein Assay reagent		Thermo Scientific	22660
NuPAGE Sample Reducing Agent		Invitrogen	NP0009
Novex WedgeWell		Thermo Fisher	XP04205BOX
Amersham ECL Western Blotting Reagent		Sigma-Aldrich	GERPN2235
CometAssay LMAgarose		R&D Systems	4250-050-02
CometAssay Lysis Solution		R&D Systems	4250-050-01
**Critical commercial assays**			
DNA/RNA Oxidation Assay		Cayman chemicals	589320
DNeasy Blood and Tissue Kit		Qiagen	69504
**Experimental models: Organisms/strains**			
C57Bl/6J male mice		Jackson Labs	000664
NIH/3T3 fibroblasts		ATCC	CRL-1658
**Software and algorithms**			
ImageJ:Fiji		imagej.net/software/fiji/	Version 2.15.0
Prism Graph Pad		www.graphpad.com/features	version 9.5.1
CometAssay Analysis Software		R&D Systems	4260-000-CS
VersaDoc MP 4000 Imaging System		Biorad	Quantity One 1-D
Image Lab software		Biorad	V1
ZEN Black		Zeiss	2.1 SP3 FP3

Supplementary Table 2. Antibody testing

DNA Repair Pathway	Protein	WB Tried?	Antibodies Tried (Species)	Vendor	Catalog Number	Worked? (Mouse brain tissue lysates)	Worked? (Mouse astrocyte P1 culture lysates)
Homologous Recombination (HR)	BRCA1	Yes	Ms	Novus Biologicals	MAB22101		No
	CtIP	Yes	Ms	Active Motif	61141	No	No
	BRCA2	No					
Non-homologous End Joining (NHEJ)	Ku70	Yes	Ms	Invitrogen	MA5-13110	No	No
			Ms	SCBT	sc-17789	No	
			Rb	CST	4588S		Yes
	Ku80	Yes	Rb	Thermo Fisher	PA5-17454	Not sure; many bands detected	
			Ms	SCBT	sc-515736	Yes	Yes
	DNA-PK	Yes	Rb	Abcam	ab32566	Yes, but band around 150 kDa	Yes, but smaller band around 150 kDa also detected
	Artemis	Yes	Ms	Bethyl Laboratories	A304-902A-M	Not sure; many bands detected	Yes, but band appears near 50 kDa
Base Excision Repair (BER)	APE1	Yes	Ms	Novus Biologicals	13B8E5C2	Yes	
	OGG1	Yes	Rb	Novus Biologicals	NB100-106	Yes, but lots of bands	
	PARP-1	Yes	Ms	Bio-Rad	MCA1522G	No	
Nucleotide Excision Repair (NER)	ERCC1	Yes	Ms	SCBT	sc-17809	Yes	Yes
	XPF	Yes	Ms	SCBT	sc-136153	No	No
	XPG	No					
Mismatch Repair (MMR)	MSH2	Yes	Rb	Abcam	ab92473	Yes	Yes
	MSH3	Yes	Ms	BD Biosciences	611390	No	
			Rb	Thermo Fisher	PA5-75306	No	
			Ms	EMD Millipore	MABE324	Yes	Yes
	MSH6	Yes	Rb	Abcam	ab92471	Yes	Yes
