# Supplementary material for: Double strand breaks drive toxicity in a Huntington’s disease mouse model with or without somatic expansion
Source: Nat Commun. 2026 May 6;17:6381. doi: 10.1038/s41467-026-72382-z (PMC13377174; doi:10.1038/s41467-026-72382-z)
Supplement: Supplementary file 3 — Transparent Peer Review file [file 41467_2026_72382_MOESM3_ESM.pdf]

nature portfolio

Peer Review File

Double strand breaks drive toxicity in Huntington's disease mice with or without somatic expansion.

Corresponding Author: Dr Cynthia McMurray

This file contains all reviewer reports in order by version, followed by all author rebuttals in order by version.

Version 0:

Reviewer comments:

Reviewer #1

(Remarks to the Author)
Numerous lines of evidence now strongly support the contention that mismatch repair driven somatic expansion of the HTT CAG repeat is a key driver of disease onset in Huntington disease in humans. Indeed, recent evidence from post-mortem human brains supports the concept that in HD patients typically inheriting 40 to 50 CAGs, somatic expansion is absolutely necessary to mediate the disease and that striatal neurons do not die until they acquire somatically expanded alleles > 150 repeats. Nonetheless, it remains possible that, somatic expansion is not absolutely required, but acts as an accelerant. Either way, it is generally assumed that the expanded CAG repeat at the DNA level is not in and of itself inherently toxic, but that toxicity is mediated via a second step in the pathway via a toxic RNA, or most likely an effect of the mutant HTT protein that contains an expanded polyglutamine domain encoded by the CAG repeat. Notably, mouse models inheriting alleles of 40 to 50 repeats, the size range typically observed in humans, do not develop abnormal pathology within the lifetime of the mouse. However, mouse models in which alleles of > 100 repeats have been introduced do usually develop symptoms and a wide array of cellular process have been demonstrated to be dysregulated in these animals. Importantly, although somatic expansion is observed in these animal models, several analyses have revealed that in mice inheriting such large expansions, somatic expansion is not required to generate pathology. The most credible interpretation of these data is that alleles of 40 to 50 repeats are insufficiently large to cause pathology in the lifetime of a mouse and that alleles >> 100 repeats are already sufficiently large to cause pathology without a requirement for additional somatic expansion. Thus, whilst somatic expansion is recognised as a key driver of disease onset in humans, the precise nature of the downstream toxic elements and the pathway from the CAG repeat in the DNA to cellular dysfunction and death remain unresolved. A key issue here is that in mouse models inheriting >> 100 repeats, a wide variety of cellular processes have been observe to be dysregulated, but the relative importance and order of events, and which are truly critical in humans and the best targets for therapy is unclear.

In this study the authors present data that in homozygous mice inheriting alleles > 150 CAG repeats there is evidence for the accumulation of double stranded DNA breaks (DSBs), possibly mediated by an abnormal interaction between mutant HTT protein and components of the double strand break repair pathway (DSBR). The authors also observe that in a genetic background deficient for the DNA mismatch repair gene Msh3, in which somatic expansions do not occur, the accumulation of double strand breaks is still observed. The authors interpret this observation as evidence that the DSBR defect is independent of somatic expansion. Whilst this may be true in the animal models used, the authors present no data from heterozygous humans that the DSBR phenotype is observed in cells expressing HTT with non-somatically expanded alleles at endogenous levels. The implication therefore that DSBR is as an important target as somatic expansion in humans may be premature. Of course, even, if as seems likely, the DSBR defect is secondary to somatic expansion in humans, this does not preclude the possibility that the DSBR defect may make a good therapeutic target. However, the authors data are largely correlative, and they have not established a direct cause and effect relationship. Thus, where these defects may rank relative to the many other defects observed in HD remains unclear. In this context, the title of the manuscript is highly misleading. In addition to the generalisability of the results and their relative importance, there are a number of other issues the authors should address:

1) The authors need to confirm clearly in the manuscript the genetic background, zygosity and numbers of all the mice used. It appears to me that wild-type mice were not litter-mate controls, but rather derived from a separate colony of C57Bl6 mice. Given the very small number of biological replicates utilised, mostly only three, but as few as a single mouse in some experiments, this is a major concern and renders it difficult, if not impossible, to separate background effects from effects of

the expanded HTT allele. Given the known hypomorphic nature of the HTT expansion how do we know these are not HTT loss of function effects? Where any of these effects observed in heterozygous Hdh150 mice? The authors also need to clearly justify and delineate how they accounted for the biological and technical replicates in their statistical analyses. In particular the authors should comment on: 1.1) The authors claim that NHEJ and HR were down in HD mutant mice (Figure 4C), but there are no stats to support this, and looking at the high degree of variance and the relatively low number of biological and technical replicates I assume none of these are significant, especially after correcting for multiple testing. 1.2) What are the stats for Figure 5F though? There are only three mice, yet dozens of points? The key difference here is that between wt and HD mice, are these differences statistically significant? 1.3) the data in Figure 6K are from a single mouse of each genotype, and whilst the data suggest that the two mice are different from each other, without additional biological replicates we can draw no inferences about the degree of variance in the mouse population and the driver of this difference.
2) The authors show data that the mutant HTT protein binds to various components of the DSBR pathway, and that the DSBR appears to be dysregulated, but there is no direct link. How do we know that DSBR dysfunction is not a common feature of polyglutamine toxicity independent of the HTT interactions?
3) The introduction is not well written, argued and very poorly referenced. The data on the impact and direction of effects of the human genetic modifiers is particularly unclear and frequently conflates genetic associations with clinical phenotypes and those with somatic expansion directly. For instance, “The DNA repair machinery are strong modifiers of somatic expansion, with MMR proteins being perhaps the most important enhancers (41-45).” This is vague and not supported by the references. Refs 41-43 are reviews of CRISPR based therapeutic approaches, and while refs 44 and 45 identify genetic variants in MMR genes as modifying clinical phenotypes, they contain absolutely no data on somatic expansion. In addition to the bountiful mouse data, references supporting a robust role for MMR gene variants in modifying HTT somatic expansion in humans are limited to [1-3]. And, “in humans and in model systems for disease, the longer tracts have earlier onset and more severe HD phenotypes, which appear to have a greater dependence on the somatic tract rather than the encoded polyglutamine protein.” This is unclear. The data reveal that inherited pure CAG length is the best predictor of age at onset and is more informative that the inherited polyglutamine length encoded. The interpretation of these findings is that as inherited pure CAG length is also the best predictor of the rate of somatic expansion, that somatic expansion is an important part of the disease process. This needs to be explained much more clearly. The authors also need to much more clearly set out the presumed the role of somatic expansion in driving the DNA to a level that mediates downstream pathology as two potentially separable stages of the disease process.
4) The first results section looking at cell type specific protein expression of MSH3 and MSH6 is generally nice, although not clear it tells us much over and above the Matlik paper [4] which had more details on different types of neurons. Of particular interest which neurons were detected in the cerebellum samples and were the ultra-sensitive Purkinje cells assessed? The authors data should also be compared to similar previous analyses in HD mouse models [5].
5) How do the authors data relate to the data of the Truant lab that have also postulated a role for HTT in various aspects of non-MMR DNA repair?
6) Throughout the authors suggest that the HTT CAG repeat is somatically expanded in Purkinje cells, yet these cells are relatively spared in HD setting this up as a major dichotomy. What they fail to note however, is that although the repeat is more somatically unstable in Purkinje cells than other cells in the cerebellum, the actual degree of somatic expansion in Purkinje cells is very modest compared to that observed in the medium striatal neurons that are the primary target of neurodegeneration in HD.
7) The authors have published a series of papers investigating the protective role of XJB-5-131 in which they suggest that is an “efficient” suppressor of somatic expansion and attribute its activity to this effect. Yet here, they see only a “small effect”, and even that claim is not supported by visual inspection of the data or by any statistical analyses. How do the authors explain these discrepancies?
8) The authors postulate that the DSBs mediate transcriptional dysregulation through a rather tenuous direct effect on the disruption of individual genes. Given the highly reproduceable nature of the transcriptional dysregulation seen in HD mouse models, it seems unlikely that a small number of largely random double strand breaks throughout the genome would coalesce on a reproducible effect on transcription. More importantly, the authors present no data that correcting the DSB defect has any effect on transcription.
9) I am not sure how to interpret the 10^5 signal for DSBs per cell, but I thought DSBs were highly damaging and even a handful would result in programmed cell death?

Minor points
i) Too many obscure abbreviations e.g., STR for striatum saves 5 letters and is confusing given that the HTT repeat is also a simple tandem repeat and that this is the most common use of the STR abbreviation.
ii) Remove redundant acronyms such as SDS-PAGE gels

References
1. Genetic Modifiers of Huntington’s Disease Consortium, et al., Genetic modifiers of somatic expansion and clinical phenotypes in Huntington’s disease reveal shared and tissue-specific effects. bioRxiv, 2024: p. 2024.06.10.597797.
2. Ciosi, M., et al., A genetic association study of glutamine-encoding DNA sequence structures, somatic CAG expansion, and DNA repair gene variants, with Huntington disease clinical outcomes.EBioMedicine, 2019. 48: p. 568–580.
3. Flower, M., et al., MSH3 modifies somatic instability and disease severity in Huntington's and myotonic dystrophy type 1. Brain, 2019. 142: p. 1876–1886.
4. Mätlik, K., et al., Cell-type-specific CAG repeat expansions and toxicity of mutant Huntingtin in human striatum and cerebellum. Nature Genetics, 2024. 56(3): p. 383–394.
5. Mason, A.G., et al., Expression levels of DNA replication and repair genes predict regional somatic repeat instability in the brain but are not altered by polyglutamine disease protein expression or age.Human Molecular Genetics, 2014. 23(6): p.

1606-1618.

Reviewer #2

(Remarks to the Author)
The present manuscript by Polyzos et al. provided strong evidence supporting the role of NHEJ, the dominant DSBR pathway in brain cells, in the development of Huntington's disease (HD) pathogenesis. Multiple studies and reports indicate that somatic CAG repeat expansion in the HTT gene is a significant driver of HD pathogenesis. However, the authors have argued that DSBR deficiency also can independently contribute to disease onset and progression. Using biochemical, cell biological and in vivo mice experiments, it has been shown that mutant huntingtin impaired NHEJ that led to similar transcriptional pathology, as was observed with conventional CAG repeat expansion. This is an important observation and consistent with several recent reports. Over the last few years, it has been shown that impaired PNKP-mediated NHEJ repair is a key factor in polyglutamine diseases, including Spinocerebellar ataxia type 3 (SCA3) and HD (PMIDs: 32205441; 39298485). Studies have convincingly shown that PNKP activity is severely abrogated in disease cells, mice and patient tissue extracts leading to persistent DSB accumulation in HD, which is consistent with the data presented in this manuscript. Hence, such findings can be discussed in a proper context.
Overall, the study provided a deeper understanding of the mechanistic basis of the disease, paving the way for potential treatments of HD, a currently incurable neurodegenerative disorder.
Despite the comprehensive nature of the study, some additional clarifications and more details on the methods are needed before it becomes suitable for publication in Nature communications.
Other comments:
MSH3 (driver of somatic expansion) is present relatively in a higher amount in striatal neurons; in contrast, glial cells show negligible MSH3 expressions. What is the possible mechanism of CAG repeat expansion in glial cells? Some discussion will be helpful for common readers.
For the IP-MS experiment (Fig 3), the authors have used a "non-specific control antibody" to correct the background signal. However, no specific information is provided about this control antibody. This could be clarified. The method section could be more elaborated on providing sufficient detail for readers to understand and potentially replicate the study. Specifically, how the interaction intensities were calculated, and how they were normalized with any background signal?
To measure the activity of various DNA repair pathways (NHEJ and HR) the reporter plasmid has been used (Fig 4); however, the design is not very clear. For NHEJ, a blunt ended DSB was used while for HR an overhang-containing DSB was used. Without specific DNA/Amino acid sequence information at the break site, it is not possible to assess potential consequences of repair synthesis. Since this DSBR assay involves expression of GFP protein; hence maintenance of proper GFP sequence would be crucial for successful expression of the protein. Therefore, a schematic with proper sequence description will be very helpful for the common readers to follow the assay system.
The authors have selected APE1 as a representative protein involved in BER pathway for the reporter plasmid-based assay. However, oxidized DNA bases are abundant DNA lesions, and their repair is initiated by one of many DNA glycosylases. Moreover, an independent and parallel NEIL-PNKP mediated BER sub-pathway is also operative. Therefore, the diversity of these enzymes and their overlapping substrate specificities make it challenging to select a single representative protein. AP sites are also quite abundant DNA lesions in mammalian genomes. So, we suggest rephrasing the rationale for using THF-containing plasmid substrate.
No statistical analysis is provided for Figure 4C. Inhibition of HR is as significant as that of NHEJ, given that NHEJ is the major DSB repair pathway in the brain cells?
For in vivo staining of mice brain (Fig. 5E), 53BP1 was used as DSB marker. While 53BP1 is known for its role in NHEJ, assessing phosphorylation status (p53BP1) can provide more specific information. The authors should clarify whether they used phospho-53BP1 or total 53BP1. The in vivo staining experiment is difficult to follow. Though 53BP1 was used as a marker, no quantification data is shown for such analysis. A representative panel at 2 h post-recovery is shown (Fig 5E); it is recommended that the authors display all relevant time points. Finally, the quantification of γH2AX foci in HD mice shows a significant decrease by 4 h post-irradiation, indicating efficient DSB repair, while such repair is not very prominent in WT mice. Some clarification could be provided about this in vivo staining data.
Fig. 7-8: Here it should also be clarified whether p53BP1 or total 53BP1 Ab was used for these experiments. The presence of 53BP1 foci in such cells further confirmed that the DNA repair mechanism used in those cells is NHEJ. However, we would like to suggest investigating RAD51 foci, a marker for homologous recombination (HR), as well. Therefore, by examining both foci formations, researchers can determine the repair pathway being utilized in the cell and will help to understand the DNA damage response.
Minor comments:
Line 78: reference citation needs to be corrected.
Line 123: "However, MutSβ drives somatic expansion of CAG triplet repeats in knockout mouse
crosses": It is better to clarify the term "knockout mouse crosses".
Line 261: Rewrite: "The activity of both HR and NHEJ activity is naturally low in the WT brain".

Reviewer #3

(Remarks to the Author)
I co-reviewed this manuscript with one of the reviewers who provided the listed reports. This is part of the Nature Communications initiative to facilitate training in peer review and to provide appropriate recognition for Early Career Researchers who co-review manuscripts.

Version 1:

Reviewer comments:

Reviewer #2

(Remarks to the Author)
The authors in the revised version of the manuscript mostly addressed the critiques and provided explanations of how MSH3 level in glial cells correlates with the CAG repeat expansion in those cells. They have also provided rationale for considering APE1 as a potential standard for evaluating BER pathway. The authors have now cited a large body of literature that showed a shared mechanism of end processing enzyme PNKP inactivation and accumulation of DSBs in polyQ diseases. Additionally, they have clarified the experimental details, rephrased and modified the results and discussion sections and expanded the Methods.
Overall, the manuscript reads well and may now be considered for acceptance in Nature communications.

Reviewer #3

(Remarks to the Author)
I co-reviewed this manuscript with one of the reviewers who provided the listed reports. This is part of the Nature Communications initiative to facilitate training in peer review and to provide appropriate recognition for Early Career Researchers who co-review manuscripts.

Reviewer #4

(Remarks to the Author)
This study presents an interesting and potentially important story, aiming to separate the contributions of CAG repeat expansion from other DNA damage in Huntington's disease. The idea that double-strand breaks may act as an independent driver of pathology is a valuable perspective. Precisely because this distinction is important, the data supporting it must be especially solid.

The authors have improved the manuscript by clarifying genetic background and zygosity, expanding the introduction with appropriate human references, and explaining discrepancies around Purkinje cell instability and XJB-5-131 activity. However, they did not add substantial new data, and the central issues of replication and statistical robustness remain unresolved. Biological replication is extremely limited: several key datasets are based on n = 1 (e.g. Figure 6J/K, parts of Figure 4, Supplemental Figure 1) or n = 2 animals (Figures 1–3, Figure 7, Figure 8, Supplemental Figures 2–5). In Figure 5F, dozens of datapoints are drawn from only three mice, inflating technical over biological replication. In addition, figure legends frequently fail to state n clearly, making interpretation difficult. For n = 1, no statistics are valid and p-values should be removed; for n = 2, parametric tests are not appropriate and results should be presented descriptively. Without adequate biological replication, the statistical conclusions remain insecure.

Further methodological concerns also remain. For example, for the FM-HCR assays (Figure 4) lack pathway-specific controls, making it unclear whether the reporters are functioning as intended in these cells. The interpretation of 53BP1 immunofluorescence (Figure 7) as a readout of DNA repair is problematic, since bulk nuclear intensity does not capture the relevant foci dynamics (similarly for gamma-H2Ax). The link to transcriptional dysregulation is still correlative and not demonstrated by rescue experiments.

While the manuscript is clearer and better referenced, the central findings are undermined by insufficient replication, over-representation of technical replicates, and weak mechanistic validation. The story is interesting and timely, but the data in its current form are not sufficiently robust to support the strong conclusions drawn.

Version 2:

Reviewer comments:

Reviewer #4

(Remarks to the Author)
As I began to reassess the manuscript point-by-point, I noted that in their rebuttal, the authors referred to the source data files, which I had not scrutinised in detail during the first round, trusting that the figure representations accurately reflected the underlying data. Upon reviewing these files now, I have identified major discrepancies between the source data values, the plotted figure outputs, the sample labelling (including timepoints), and the described normalisation procedures.

I would strongly recommend that the authors carefully re-examine all quantitative results presented in the manuscript — including raw data, figure annotations, plotted values, normalisation procedures, statistical treatment, and sample metadata — to ensure full internal consistency and traceability.

For example, the source data file provided for Figures 1–3 uses timepoints labelled as 10–11 weeks and 75–76 weeks, whereas Figure 1 is labelled as 7–10 weeks and 70–90 weeks (Figure 2 labels it correctly for the two datapoints where blots are shown). In addition, the third "replicate" used for quantification in some cases originates from 19–20 weeks, which is

biologically not part of either of the two age groups depicted and therefore should not be used as a biological replicate. If included, this would artificially expand the age range and potentially confound the results. To maintain biological consistency, the labelling should more accurately reflect what was actually used (e.g., 10–20 weeks vs 75–76 weeks), or the dataset should be corrected to include only valid age-matched replicates.

Additionally, the quantification appears to be normalised only to WT without applying loading control correction, despite GAPDH bands being shown in some supplementary blots (no blots shown for the third replicate). The source data file does not contain loading-control–adjusted numerical values, nor any indication of background subtraction or target/loading ratios. As a result, the plotted values cannot be fully reconciled with the raw data shown in the blots.

There are also cases where the numerical data provided DOES NOT match the plotted values. For instance, for MSH3 at 10–11 weeks, the source data report:

CBL STR
WT HD WT HD
Rep 1 1 0.9917 0.3170 0.2735
Rep 2 1 1.0304 0.3149 0.2185

However, in the figure, HD CBL is shown close to 2.0, which is not supported by the reported values (0.99 and 1.03).

Similar discrepancies appear for XPA: the plots for 10-week and 75-week XPA data look nearly identical, yet the source data file reports clearly different numerical values for those timepoints:

10 weeks (source data):

CBL STR
WT HD WT HD
Rep 1 1 2.0895 0.9757 1.6014
Rep 2 1 2.8115 0.9282 1.3066

75 weeks (source data):

CBL STR
Rep 1 1 1.2277 0.3906 0.8229
Rep 2 1 2.2005 0.4764 0.7493

Yet, the plotted bars appear nearly the same across both ages, for example, in STR-HD at 10 weeks, the values are about 1.1 and 2.2.

Furthermore, some error bars are truncated (including datapoints, for example, MRE11 at 10 weeks), axis labels appear to have been manually edited (with earlier labels faintly visible beneath), and y-axis ranges are inconsistently adjusted across panels, limiting reliable comparison across conditions.

Considering these various issues, verifying the derivation of the final plotted data is challenging. At this point, I recommend that the authors thoroughly review all data processing steps, including normalisation procedures, figure plotting, and labelling, to verify that the raw data, source files, and visual representations are fully consistent and traceable. I offer these comments in a constructive spirit, recognising the valuable biological questions addressed. Nonetheless, improving clarity, consistency between raw and plotted data, and methodological transparency would significantly strengthen the manuscript and improve its reproducibility. Due to these fundamental concerns, I have not assessed the rest of the manuscript in detail, as these issues should be resolved first.

Version 3:

Reviewer comments:

Reviewer #4

(Remarks to the Author)
All my requests for correction have been addressed.

Open Access This Peer Review File is licensed under a Creative Commons Attribution 4.0 International License, which permits use, sharing, adaptation, distribution and reproduction in any medium or format, as long as you give appropriate credit to the original author(s) and the source, provide a link to the Creative Commons license, and indicate if changes were made.
In cases where reviewers are anonymous, credit should be given to 'Anonymous Referee' and the source.
The images or other third party material in this Peer Review File are included in the article’s Creative Commons license, unless indicated otherwise in a credit line to the material. If material is not included in the article’s Creative Commons license and your intended use is not permitted by statutory regulation or exceeds the permitted use, you will need to obtain permission directly from the copyright holder.
To view a copy of this license, visit https://creativecommons.org/licenses/by/4.0/

Reviewer Response

Rev, 1

"Numerous lines of evidence now strongly support the contention that mismatch repair driven somatic expansion of the HTT CAG repeat is a key driver of disease onset in Huntington disease in humans. Indeed, recent evidence from post-mortem human brains supports the concept that in HD patients typically inheriting 40 to 50 CAGs, somatic expansion is absolutely necessary to mediate the disease and that striatal neurons do not die until they acquire somatically expanded alleles > 150 repeats. Nonetheless, it remains possible that, somatic expansion is not absolutely required but acts as an accelerant. Either way, it is generally assumed that the expanded CAG repeat at the DNA level is not in and of itself inherently toxic, but that toxicity is mediated via a second step in the pathway via a toxic RNA, or most likely an effect of the mutant HTT protein that contains an expanded polyglutamine domain encoded by the CAG repeat. Notably, mouse models inheriting alleles of 40 to 50 repeats, the size range typically observed in humans, do not develop abnormal pathology within the lifetime of the mouse. However, mouse models in which alleles of > 100 repeats have been introduced do usually develop symptoms and a wide array of cellular process have been demonstrated to be dysregulated in these animals. Importantly, although somatic expansion is observed in these animal models, several analyses have revealed that in mice inheriting such large expansions, somatic expansion is not required to generate pathology. The most credible interpretation of these data is that alleles of 40 to 50 repeats are insufficiently large to cause pathology in the lifetime of a mouse and that alleles >> 100 repeats are already sufficiently large to cause pathology without a requirement for additional somatic expansion. Thus, whilst somatic expansion is recognised as a key driver of disease onset in humans, the precise nature of the downstream toxic elements and the pathway from the CAG repeat in the DNA to cellular dysfunction and death remain unresolved. A key issue here is that in mouse models inheriting >> 100 repeats, a wide variety of cellular processes have been observed to be dysregulated, but the relative importance and order of events, and which are truly critical

"In this study the authors present data that in homozygous mice inheriting alleles > 150 CAG repeats there is evidence for the accumulation of double stranded DNA breaks (DSBs), possibly mediated by an abnormal interaction between mutant HTT protein and components of the double strand break repair pathway (DSBR). The authors also observe that in a genetic background deficient for the DNA mismatch repair gene Msh3, in which somatic expansions do not occur, the accumulation of double strand breaks is still observed. The authors interpret this observation as evidence that the DSBR defect is independent of somatic expansion. Whilst this may be true in the animal models used, the authors present no data from heterozygous humans that the DSBR phenotype is observed in cells expressing HTT with non-somatically expanded alleles at endogenous levels. The implication therefore that DSBR is as an important target as somatic expansion in humans may be premature. Of course, even, if as seems likely, the DSBR defect is secondary to somatic expansion in humans, this does not preclude the possibility that the DSBR defect may make a good therapeutic target. However, the authors data are largely correlative, and they have not established a direct cause and effect relationship. Thus, where these defects may rank relative to the many other defects observed in HD remains unclear. In this context, the title of the manuscript is highly misleading. In addition to the generalisability of the results and their relative importance, there are a number of other issues the authors should address:

1)The authors need to confirm clearly in the manuscript the genetic background, zygosity and numbers of all the mice used. It appears to me that wild-type mice were not littermate controls, but rather derived from a separate colony of C57Bl6 mice. Given the very small number of biological replicates utilised, mostly only three, but as few as a single mouse in some experiments,

this is a major concern and renders it difficult, if not impossible, to separate background effects from effects of the expanded HTT allele. Given the known hypomorphic nature of the HTT expansion how do we know these are not HTT loss of function effects? Where any of these effects observed in heterozygous Hdh150 mice? (The authors also need to clearly justify and delineate how they accounted for the biological and technical replicates in their statistical analyses. In particular the authors should comment on: 1.1) The authors claim that NHEJ and HR were down in HD mutant mice (Figure 4C), but there are no stats to support this, and looking at the high degree of variance and the relatively low number of biological and technical replicates I assume none of these are significant, especially after correcting for multiple testing. 1.2) What are the stats for Figure 5F though? There are only three mice, yet dozens of points? The key difference here is that between wt and HD mice, are these differences statistically significant? 1.3) the data in Figure 6K are from a single mouse of each genotype, and whilst the data suggest that the two mice are different from each other, without additional biological replicates we can draw no inferences about the degree of variance in the mouse population and the driver of this difference. 2) The authors show data that the mutant HTT protein binds to various components of the DSBR pathway, and that the DSBR appears to be dysregulated, but there is no direct link. How do we know that DSBR dysfunction is not a common feature of polyglutamine toxicity independent of the HTT interactions? (3) The introduction is not well written, argued and very poorly referenced. The data on the impact and direction of effects of the human genetic modifiers is particularly unclear and frequently conflates genetic associations with clinical phenotypes and those with somatic expansion directly. For instance, "The DNA repair machinery are strong modifiers of somatic expansion, with MMR proteins being perhaps the most important enhancers (41-45)." This is vague and not supported by the references. Refs 41-43 are reviews of CRISPR based therapeutic approaches, and while refs 44 and 45 identify genetic variants in MMR genes as modifying clinical phenotypes, they contain absolutely no data on somatic expansion. In addition to the bountiful mouse data, references supporting a robust role for MMR gene variants in modifying HTT somatic expansion in humans are limited to [1-3]. And, "in humans and in model systems for disease, the longer tracts have earlier onset and more severe HD phenotypes, which appear to have a greater dependence on the somatic tract rather than the encoded polyglutamine protein." This is unclear. The data reveal that inherited pure CAG length is the best predictor of age at onset and is more informative that the inherited polyglutamine length encoded. The interpretation of these findings is that as inherited pure CAG length is also the best predictor of the rate of somatic expansion, that somatic expansion is an important part of the disease process. This needs to be explained much more clearly. The authors also need to much more clearly set out the presumed the role of somatic expansion in driving the DNA to a level that mediates downstream pathology as two potentially separable stages of the disease process. 4) The first results section looking at cell type specific protein expression of MSH3 and MSH6 is generally nice, although not clear it tells us much over and above the Matlik paper [4] which had more details on different types of neurons. Of particular interest which neurons were detected in the cerebellum samples and were the ultra-sensitive Purkinje cells assessed? The authors data should also be compared to similar previous analyses in HD mouse models [5]. 5) How do the authors data relate to the data of the Truant lab that have also postulated a role for HTT in various aspects of non-MMR DNA repair?1.6) Throughout the authors suggest that the HTT CAG repeat is somatically expanded in Purkinje cells, yet these cells are relatively spared in HD setting this up as a major dichotomy. What they fail to note however, is that although the repeat is more somatically unstable in Purkinje cells than other cells in the cerebellum, the actual degree of somatic expansion in Purkinje cells is very modest compared to that observed in the medium striatal neurons that are the primary target of neurodegeneration in HD. (1.7) The authors have published a series of papers investigating the protective role of XJB-5-131 in which they suggest that is an "efficient" suppressor of somatic expansion and attribute its activity to this effect. Yet here, they see only a "small effect", and even that claim is not supported by visual inspection

of the data or by any statistical analyses. How do the authors explain these discrepancies? 1.8) The authors postulate that the DSBs mediate transcriptional dysregulation through a rather tenuous direct effect on the disruption of individual genes. Given the highly reproduceable nature of the transcriptional dysregulation seen in HD mouse models, it seems unlikely that a small number of largely random double strand breaks throughout the genome would coalesce on a reproducible effect on transcription. More importantly, the authors present no data that correcting the DSB defect has any effect on transcription. 1.99) I am not sure how to interpret the 10^5 signal for DSBs per cell, but I thought DSBs were highly damaging and even a handful would result in programmed cell death?

Minor points
i) Too many obscure abbreviations e.g., STR for striatum saves 5 letters and is confusing given that the HTT repeat is also a simple tandem repeat and that this is the most common use of the STR abbreviation.
ii) Remove redundant acronyms such as SDS-PAGE gels

RESPONSE TO GENERAL COMMENTS IN PARAGRAPHS 1 AND 2

(a) Our models is consistent with current thinking, as summarized by the reviewer, *"it is generally assumed that the expanded CAG repeat at the DNA level is not in and of itself inherently toxic, but that toxicity is mediated via a second step in the pathway via a toxic RNA, or most likely an effect of the mutant HTT protein that contains an expanded polyglutamine domain encoded by the CAG repeat"* We find the CAG somatic expansion is not in itself inherently toxic (no toxicity is observed before 1.2 years), but toxicity is mediated in a second step in which mhtt (polyglutamine protein) inhibits DNA repair through an interaction action with the DSBR machinery.

(b) "*Notably, mouse models inheriting alleles of 40 to 50 repeats, the size range typically observed in humans, do not develop abnormal pathology within the lifetime of the mouse. The most credible interpretation of these data is that alleles of 40 to 50 repeats are insufficiently large to cause pathology in the lifetime of a mouse and that alleles >> 100 repeats are already sufficiently large to cause pathology without a requirement for additional somatic expansion.*

In our opinion, this interpretation does not adequately consider the damage load for humans and mice within their distinct lifetimes. Humans of 40-50 CNG repeats have disease onset at roughly 40-60 years, providing a long-time frame to accumulate damage, while mice live only 1-3 years. This would be like comparing a mouse of 40-50 repeats to a 2-year-old HD human, who would not display toxicity. We envision that if the mouse lived for 50-60 more years (comparable to humans), pathology would be likely. Our best interpretation of the data is that mice of 40-50 repeats do not accumulate enough damage to induce toxicity during its life, despite the tract reaching the human threshold of disease. To test this idea, our strategy was to use a late onset mouse with a longer CAG tract and delayed onset, thereby creating a pathology-free window and an age-dependent pathology window in the same mouse within their lifetime._We find that DSBs are low in the early in window and there is no toxicity, while damage is substantially higher at later ages in the pathology window (Fig. 6). At the same time, when we suppress DSBs in mice by XJB-5-131 treatment, neuropathology is blocked for the longer repeat and even if somatic expansion is active (Fig. 8) (i.e. without the need for further somatic expansion).

(c) *"whilst somatic expansion is recognised as a key driver of disease onset in humans, the precise nature of the downstream toxic elements and the pathway from the CAG repeat in the DNA to cellular dysfunction and death remain unresolved" A key issue here is that in mouse models inheriting >> 100 repeats, a wide variety of cellular processes have been observed to be dysregulated, but the relative importance and order of events, and which are truly critical*

Since not all functions of mhtt and htt scaffolds are known or well characterized, we did not attempt to order them or to exclude them. Rather, we focused on testing DNA repair, which is central in promoting CAG expansion and disease. MSH3 drives expansion but is involved in other DNA repair pathways, which had not been thoroughly examined. In testing possible importance of other DNA repair pathways, we discovered that DSBR was inhibited in HD mice and that suppression of the resulting DSBs blocked neuropathology, despite possible disturbances in other cellular processes. DSBR is an important disease-relevant process, which was followed.

[Added to text]

(d) *The authors observe that in a genetic background deficient for the DNA mismatch repair gene Msh3, in which somatic expansions do not occur, the accumulation of double strand breaks is still observed. The authors interpret this observation as evidence that the DSBR defect is independent of somatic expansion. However, the authors data are largely correlative,*

The mechanism for neurotoxicity in HD cannot be solved in a single publication, but every publication contributes to our understanding of the disease. Thus, our goal as scientists is to generate testable models based on experimental data and the best interpretation of the results. The model proposed here was derived from direct and strong results from many experiments including DNA repair activity, protein analysis, mouse mutational analysis, in vitro pull-down analysis, and other published work, together with cause-effect reversibility studies in transgenic animals. Collectively, integration of these data suggests a reasonable and testable model in which DSBs, and somatic expansion coexist as independent components of toxicity, driving the downstream pathology as two potentially separable stages of the disease process. As with any model, it will evolve with further testing or as more in known.

We outline the experimental basis for features of the model:

- DSBR is inhibited in HD animals (Fig. 4 and 5) and DSBs rise (Fig. 6) and accumulate in expansion-capable mice.

- Gene knockout tests are among the most rigorous functional tests of relevance. In HD/MSH3(-/-) crosses, somatic expansions do not occur, but the accumulation of double strand breaks is still observed (Fig. 8). DSBs and somatic expansion are independent.

- In the absence of somatic expansion, transcriptional dysfunction measured in *HD/MSH3(-/-)* crosses increases with the rise in DSBs (Fig.7). Features of neuropathology correlates with DSBs without the need for somatic expansion and despite other possible disturbances in ceullar processes.

- XJB-5-131 reduces DSBs and reverses HD toxicity even though somatic expansion is active. Toxicity is rescued when DSBs are removed, independent of somatic expansion and despite possible disruption of other cellular processes. This is observed not only by DNA markers

but also by direct reversal of the physical DNA breaks (comets). DSBs promote neurotoxicity despite other possible disturbances in cellular processes.

- That XJB-5-131 establishes a direct cause-effect relationship between DSBs and neurotoxicity and was validated by the remaining data in published work *(Polyzos et al., NatComm. 2024.)*

  XJB-5-131 blocks damage from physiological causes of neuronal damage and DSBs. We oxidized cells with peroxide treatment to induce SSB to DSB conversion would elevate DSBs. If they were caused by oxidation, these DSBs would be reversible by XJB-5-131. Indeed, treatment with XJB-5-131 suppresses DSBs in peroxide treated cells; XJB-5-131 also reduced DSBs in animal brains *in vivo (Polyzos et al., NatComm. 2024)*

  XJB-5-131 blocks damage from physiological causes of neuronal damage and DSBs. To confirm, SSBs were generated enzymatically by transient transfection of recombinant Cas9D10A. This mutation converts the (CRISPR) Cas9 nuclease into a "nickase" capable of introducing only one SSB per reaction in genomic DNA of cells. The closely spaced SSBs converted to DSBs within 30 minutes, but the enzymatically generated DSBs were not reversible with XJB-5-13. *(Polyzos et al., NatComm. 2024*

  Reduction in DSBs by XJB-5-131 did not occur by another mechanism. XJB-5-131 had no impact on formation of DSBs themselves. The Cas9 nuclease enzymatically induces DSBs without oxidation or a SSB intermediate. DSBs formed after Cas9 transcfection were not reversible by XJB-5-131 treatment. *(Polyzos et al., NatComm. 2024)*

  We find it difficult to interpret the set of experiments in any other way than somatic expansion and DSBs coexist but are separable process.

(e) *Whilst this may be true in the animal models used, the authors present no data from heterozygous humans that the DSBR phenotype is observed in cells expressing HTT with non-somatically expanded alleles at endogenous levels*.

Testing in heterozygous HD patients, while desirable, it is not required for validity of the results.

(e1) HD requires a single mutant allele. Even in siblings, early experiments showed that the disease onset and progression in HD patients does not depend on allele number. The same is true in mouse.

- *Myers RH, Leavitt J, Farrer LA, Jagadeesh J, McFarlane H, Mastromauro CA, Mark RJ, Gusella JF. Homozygote for Huntington disease. Am. J. Hum. Gen. 1989;45:615–618.*

- *Wexler NS, Young AB, Tanzi RE, Travers H, Starosta-Rubinstein S, Penney JB, Snodgrass SR, Shoulson I, Gomez F, Ramos Arroyo MA, Penchaszadeh GK, Moreno H, Gibbons K, Faryniarz A, Hobbs W, Anderson MA, Bonilla E, Conneally PM, Gusella JF. Homozygotes for Huntington disease. Nature. 1987;326:194–197*

- *Dürr A., Hahn-Barma V., Brice A., Pêcheux C., Dodé C., Feingold J. Homozygosity in Huntington's disease. J Med Genet. 1999 Feb;36(2):172–173.*

- *Narain Y, Wyttenbach A, Rankin J, Furlong RA, Rubinsztein DC. A molecular investigation of true dominance in Huntington's disease. J Med Genet. 1999 Oct;36(10):739-46.*

- Somatic expansion in mouse models also does not depend on allele number *(Budworth et al., 2016)*

(e2) Most DNA repair genes that were eventually validated in humans were first identified in the mouse. In other studies, mouse experiments drove discovery for human testing.

Mouse is a classic and primary model for testing mechanisms of HD. Decades of published ådependence on DNA repair in the mouse and human systems mechanistically reflect each other, the importance of the uninterrupted allele on disease onset. Humans and mice use the same repair machinery and have the lesion which they correct. The mismatch repair is a particularly good example because of its influence on somatic expansion of CAG repeats in both mouse and in humans.

- M Swami, AE Hendricks, T Gillis, T Massood, J Mysore, RH Myers, VC Wheeler. Somatic expansion of the Huntington's disease CAG repeat in the brain is associated with an earlier age of disease onset Hum Mol Genet, 18 (2009), pp. 3039-3047

- PF Shelbourne, C Keller-McGandy, WL Bi, *et al.* Triplet repeat mutation length gains correlate with cell-type specific vulnerability in Huntington disease brain. Hum Mol Genet, 16 (2007), pp. 1133-1142

- RM Pinto, E Dragileva, A Kirby, *et al.* Mismatch repair genes Mlh1 and Mlh3 modify CAG instability in Huntington's disease mice: genome-wide and candidate approaches PLoS Genet, 9 (2013), p. e1003930.

- Usdin, K., House, N.C. & Freudenreich, C.H. Repeat instability during DNA repair: Insights from model systems. *Crit Rev Biochem Mol Biol*. 50, 142-67 (2015).

- Genetic Modifiers of Huntington's Disease (GeM-HD) Consortium. Identification of Genetic Factors that Modify Clinical Onset of Huntington's Disease. Cell, 162 (2015), pp. 516-526

- J-M Lee, MJ Chao, D Harold, *et al.*a modifier of Huntington's disease onset at the MLH1 locus Hum Mol Genet, 26 (2017), pp. 3859-3867

- C Bettencourt, D Hensman-Moss, M Flower, *et al.* DNA repair pathways underlie a common genetic mechanism modulating onset in polyglutamine diseases. Ann Neurol, 79 (2016), pp. 983-990

- F Morales, M Vásquez, C Santamaría, P Cuenca, E Corrales, DG Monckton. A polymorphism in the MSH3 mismatch repair gene is associated with the levels of somatic instability of the expanded CTG repeat in the blood DNA of myotonic dystrophy type 1 patients DNA Repair, 40 (2016), pp. 57-66

(e3) **DSBR operates by a similar mechanism, and the error-prone DNA repair deficits we propose underlie other human neurodegenerative diseases.**

- DSBR machinery is the same in mouse and in human HD heterozygous cells and are genotype interchangeable when transfected in cells.

- Human brain cannot be collected in the same person over time and there is a highly variable or uncertain postmortem delay among samples (which can result in genome damage by oxidation). Therefore, DSBs may be measurable in a human brain, but the accumulation of DSBs cannot be quantified over time in the same individual. Testing is typically and quantitatively performed in genetically identical mouse lines, where these conditions can be normalized.

- There is substantial evidence that persistent DSBs, and neuronal death is caused if DSBR is disabled by mutation in both human disease and in their mouse models, with or without somatic expansion. DSBs arise from mutations in Tyrosyl DNA-phosphodiesterase 1 (TDP1), aprataxin (APTX), or polynucleotide kinase/phosphatase (PNKP). The latter is DSBR enzyme that impairs the ability to repair DSBs and are associated with spinocerebellar ataxia with axonal neuropathy (SCAN1) and ataxia-oculomotor apraxia 1 (AOA1), and spinocerebellar ataxia type 3 (SCA3), respectively.

- While in HD, there is no mutation in DNA repair machinery, particularly important, is that a similar mechansim of DSBR reduction has been reported for SCA3. In that gene, the end joining activity of PNPK activity is inhibited by the expanded ataxin 3, the gene product in SCA3, in mice and patient tissue extracts, also leading to DSB accumulation.

  [Last two points added in the revised text in discussion]

  Overall, DNA repair mechanisms and somatic expansion in mouse reasonably predict those in humans whether they express one or two alleles. Based on a vast body of literature, it would be difficult to support the claim that mechanisms identified in the mouse are unrelated to those in humans.

(f) The implication therefore that DSBR is as an important target as somatic expansion in humans may be premature. Of course, even, if as seems likely, the DSBR defect is secondary to somatic expansion in humans, this does not preclude the possibility that the DSBR defect may make a good therapeutic target.

We did not rank the relative importance of somatic expansion and DSBs as therapeutic targets They both are important and play distinct roles

(g) the authors data are largely correlative, and they have not established a direct cause and effect relationship. Thus, where these defects may rank relative to the many other defects observed in HD remains unclear. In this context, the title of the manuscript is highly misleading.

See point d above for cause-effect relationship.

See Point c above. We focused on testing DNA repair, which is central in promoting CAG expansion and disease. In testing pathways, we discovered that DSBR was inhibited in HD mice and that suppression of the resulting DSBs blocked neuropathology, despite possible disturbances in other cellular processes. The relationship between DNA repair and neuropathology is reflected in the title and does not exclude other contributors.

[Text altered to express this point]

REVIEWER #1 (RESPONSE TO 9 SPECIFIC COMMENTS IN PARAGRAPH 3)

(1) (1.1) *The authors need to confirm clearly in the manuscript the genetic background, zygosity and numbers of all the mice used. It appears to me that wild-type mice were not littermate controls, but rather derived from a separate colony of C57Bl6 mice.*

We previously reported a 1200 animal study in which the het and homozygous *HdhQ(150/150)* and C57Bl6 animal were crossed for 2-3 years to create congenic strains (*Budworth et al., Plos Genetics, 2016)*. Consistent with human analysis, the hets and homozygous animals did not differ in onset *(Budworth et al., Plos Genetics, 2016).* Because DSBS are influenced by the presence of mtt, in this set of experiments, we maintained the genetic background but continued to breed congenic hets to homozygosity (*HdhQ(150/150)* for an additional 5 generation in parallel with C57Bl6 the same genetic background isolated from the same breeding colony. Since DSB are elevated in mhtt expressing animals, this scheme allowed us to compare DSB formation in HD mice relative to control animals of the same background which did not see the mutant HD allele for 5 generations.

{This has been added to the methods}.

(1.2) *Given the n=3 very small number of biological replicates this is a major concern and renders it difficult, if not impossible, to separate background effects from effects of the expanded HTT allele.*

Statistics are adapted as appropriate for the experiment. Unlike human GWAS studies where each brain has a highly variable background and distinct genetics, three biological and technical replicates is a standard to establish an effect in a congenic strain of genetically identical animals. Per statistical principles, 3-6 animals are reliably used *when the important aspect of the measurements (i.e., in this case, the DNA repair content) is a characteristic of the population from which the individuals are obtained and not the individuals themselves*. In our experiments, the DNA repair content (Fig. 3) from five major pathways is characteristic of the line. Thus, DNA repair in our experiments is a feature of the strain, and animal-to-animal variations do NOT arise from the genetics of animal itself (which is a clonal). Thus, animal 1 establishes the effect, and was replicated in 2-6 additional genetically identical samples.

(1.3) *Given the known hypomorphic nature of the HTT expansion how do we know these are not HTT loss of function effects?*

HD is a gain of function mutation:

"*Duyao MP, Auerbach AB, Ryan A, Persichetti F, Barnes GT, McNeil SM, Ge P, Vonsattel JP, Gusella JF, Joyner AL, et al. Inactivation of the mouse Huntington's disease gene homolog Hdh. Science. 1995 21;269(5222):407-10. Also see Ambrose et al., Structure and expression of the Huntington's disease gene: evidence against simple inactivation due to an expanded CAG repeat. Somat Cell Mol Genet. 1994 Jan;20(1):27-38.*

As stated in Duyao and Gusella: "To distinguish between "loss of function" and "gain of function" models of HD, the murine HD homolog Hdh was inactivated by gene targeting. Mice

heterozygous for Hdh inactivation were phenotypically normal, whereas homozygosity resulted in embryonic death. Homozygotes displayed abnormal gastrulation at embryonic day 7.5 and were resorbing by day 8.5. Thus, huntingtin is critical early in embryonic development, before the emergence of the nervous system. That Hdh inactivation does not mimic adult HD neuropathology indicating that the human disease involves a gain of function."

***(1.4)** The authors also need to clearly justify and delineate how they accounted for the biological and technical replicates in their statistical analyses.*

(In Fig. 4C) The authors claim that NHEJ and HR were down in HD mutant mice (Figure 4C), but there are no stats to support this, and looking at the high degree of variance and the relatively low number of biological and technical replicates I assume none of these are significant, especially after correcting for multiple testing.

Good point. The statistical comparison was not thoroughly described in the results. The relative repair activity in both WT and HD cells was measured by FM-HCR but DSBR activity too low and too variable in transfected cells to determine a statistical difference in genotype. Therefore, significance of DSBR reduction was established using two additional and complementary studies-one in cells (Fig. 5A-C) and one in mouse (Fig. 5D-G).

[We have revised the text to clarify this point].

(Figure 5F) There are only three mice, yet dozens of points? The key difference here is that between wt and HD mice, are these differences statistically significant?

In old Fig. 5, we statistically compared intensity of DSBR over the time course for both WT and HD but did not statistically compare WT and HD directly to each other at each time point. In the revision, we include a new Fig. 5F and 5G, which includes that comparison. WT and HD differences are significant by 1-way ANOVA. Data in Fig. 5F were displayed as box and whisker plots. The points shown per tissue section are individual scored γH2AX signals from roughly n=50 cells for each triplicate measure. Therefore, dozens of points are expected.

[new Fig. 5F and 5G]

-(in Fig. 6K). The data in Figure 6K are from a single mouse of each genotype, and whilst the data suggest that the two mice are different from each other, without additional biological replicates we can draw no inferences about the degree of variance in the mouse population and the driver of this difference.

Fig. 6K did not arise from a single mouse. Figure 6 describes the comet analysis, which was derived from quantification of comet tail moments from dispersed cells from brain sections described in Fig, 6I (n=3 mice of each genotype and n=3 for each brain region). We have re-checked the legend to make sure this is clear.

'In legend 6I, (H,I) Single cell quantification of DSBs in neurons and glia from the IF intensity in tissue section from WT and HD mice at 7-10 wks (H) and 70-90 wks (I). Neurons were identified as NeuN(+) cells in n=3 tissue sections for n=3 mice of each genotype (see F). WT is green, HD is red. γH2AX staining was quantified from n=50 randomly selected cells of each genotype in n=3 tissue sections of 7-10 wks (left) or (n=3) 70-90 wks (right) animals, respectively. (K) Quantification of broken DNA as comet tail moments for the STR of WT (light gray) or HD (dark gray) mice from (I). Points shown are individual scored comets from at least n=550-890 cells per

tissue section per brain region. Data are displayed as a box and whisker plot, where the box is 50% of the values, the line is the median value, and 25% maximum and 25% minimum values are indicated by whiskers above and below the box, respectively. Genotype comparisons in STR of WT (light gray) and HD * dark gray) animals were determined for signifiance using a 1-way ANOVA. **** , P < 0.0001 for the comparison'

(2) *The authors show data that the mutant HTT protein binds to various components of the DSBR pathway, and that the DSBR appears to be dysregulated, but there is no direct link. How do we know that DSBR dysfunction is not a common feature of polyglutamine toxicity independent of the HTT interactions?*

This is an experiment we would have liked to perform if the allele interactions had been separable. However, by IP-MS, the DSBR machinery binds equally well to both htt and to mhtt, which share most of their structure (see Fig. 3). Thus, interfering with htt interaction with Ku70-Ku80 will also interfere with mhtt interaction under normal physiological conditions. Therefore, we relied genetics: toxicity and DSBs are elevated in animals that express only mhtt, while there is little impact on toxicity and DSBs in mice expressing only htt.

(3) *The introduction is not well written, argued and very poorly referenced. The data on the impact and direction of effects of the human genetic modifiers is particularly unclear and frequently conflates genetic associations with clinical phenotypes and those with somatic expansion directly. For instance, "The DNA repair machinery are strong modifiers of somatic expansion, with MMR proteins being perhaps the most important enhancers (41-45)." This is vague and not supported by the references. Refs 41-43 are reviews of CRISPR based therapeutic approaches, and while refs 44 and 45 identify genetic variants in MMR genes as modifying clinical phenotypes, they contain absolutely no data on somatic expansion*.

We have revised the text to more clearly state a potential role of somatic expansion in driving the downstream pathology as two potentially separable stages of the disease process. We (1) adjusted for most relevant citations, (2) clarified the language with respect to inherited pure CAG length as the best predictor of age at onset and the rate of somatic expansion. (3) We also strengthened the connection between DNA repair and somatic expansion by discussing the recent paper by V. Wheeler, who tested many of the known GWAS-associated DNA repair modifiers in mouse knockout lines and determined their impact on somatic expansion { *Mouro Pinto, R., Murtha, R., Azevedo, A. et al. In vivo CRISPR–Cas9 genome editing in mice identifies genetic modifiers of somatic CAG repeat instability in Huntington's disease. Nat Genet* ***57****, 314–322 (2025). In* every case, modifiers that accelerated HD onset in GWAS studies increased CAG tract length and those associated with delayed onset decreased CAG tract length.

[Introduction altered in text]

(4) *The first results section looking at cell type specific protein expression of MSH3 and MSH6 is generally nice, although not clear it tells us much over and above the Matlik paper which had more details on different types of neurons.*

We did not try to replicate the findings of the Matlik paper. Our goal here was only to establish that our mouse model expressed the needed MMR components to drive somatic expansion in the striatum. This had not been previously reported.

(5) How do the authors data relate to the data of the Truant lab that have also postulated a role for HTT in various aspects of non-MMR DNA repair?

Many reviews (including Truant) have discussed the many normal functions of Htt in cellular biology including non-MMR. His 2016 review is cited. However, Truant has been one of the strongest proponents of DNA damage and repair as a major underlying causes in neurodegenerative disease. Since we tested the relationship between mhtt and DNA repair, his review which highlights aspects of both normal disease effects is also cited. "Maiuri, T. *et al.* DNA Damage Repair in Huntington's Disease and Other Neurodegenerative Diseases. *Neurother.* **16**, 948-956 (2019).

From his review,,"A potential universal trigger of oxidative DNA damage shared in these late age–onset diseases is the increase of reactive oxygen species (ROS) in human aging, defining an age-related mechanism that has defied other hypotheses of neurodegeneration. We discuss the potential commonality of DNA damage repair pathways in HD and other neurodegenerative diseases".

(6) Throughout the authors suggest that the HTT CAG repeat is somatically expanded in Purkinje cells, yet these cells are relatively spared in HD setting this up as a major dichotomy. What they fail to note however, is that although the repeat is more somatically unstable in Purkinje cells than other cells in the cerebellum, the actual degree of somatic expansion in Purkinje cells is very modest compared to that observed in the medium striatal neurons that are the primary target of neurodegeneration in HD.

The somatically expanded CAG tract in Purkinje cells is a not major point in this paper. Thus, we have re-written text to remove any emphasis. Our experiments were largely performed in the striatum.

[Text is altered to remove them]

(7) The authors have published a series of papers investigating the protective role of XJB-5-131 in which they suggest that is an "efficient" suppressor of somatic expansion and attribute its activity to this effect. Yet here, they see only a "small effect", and even that claim is not supported by visual inspection of the data or by any statistical analyses. How do the authors explain these discrepancies?

We do not attribute the effects of XJB-5-131 to suppression of somatic expansion, but we can see how this could be confusing. XJB-5-131 binds to the mitochondrial membrane and operates as an efficient suppressor of toxicity by reducing oxidative DNA damage *(Wipf J. et al. 2022)* and blocks SSB to DSB conversion *(Polyzos et al., Nat Comm, 2024).* In the late onset *HdhQ150/150* animals we utilized, the reported protective role of XJB-5-131 was on mitochondrial function (somatic expansion was not measured in these studies) *(Polyzos, 2016) (Xun et al., 2012).* Although knockout of OGG1 had suppressive effects on somatic expansion in *HdhQ150/150* animals, XJB-5-131 treatment had only modest effects *(Budworth et al., 2016).* XJB-5-131 has marked suppression effects on expansion in only one mouse model, the very severe early onset R6/2 mice, at late stages as they approach death (These mice die rapidly typically within 12 weeks)*(Polyzos et al., 2018),* The R6/2 model is not physiological -- it overexpresses a short peptide containing the polyQ region (90 amino acids of the 3144aa in the htt protein). We presume, in this severe setting, that oxidative stress near death is so high and frequent that BER is overtaxed such that a reduction in ROS has a measurable suppressive effect on somatic

expansion. Under physiological conditions, however, in the mild, late onset disease models that we use, the endogenous full-length protein is expressed, the animals live for years, and under those conditions, the level of oxidation does not overtax BER and the impact of XJB-5-131 on expansion is modest, as we have reported in several publications.

(8) The authors postulate that the DSBs mediate transcriptional dysregulation through a rather tenuous direct effect on the disruption of individual genes. Given the highly reproducible nature of the transcriptional dysregulation seen in HD mouse models, it seems unlikely that a small number of largely random double strand breaks throughout the genome would coalesce on a reproducible effect on transcription. More importantly, the authors present no data that correcting the DSB defect has any effect on transcription.

- This is a highly relevant and important point, and we have revised the discussion to highlight this issue. We agree. It is unlikely that a small number of largely random double strand breaks throughout the genome would coalesce on a reproducible effect on transcription. However, the number DSBs is likely to be higher than estimated since a snapshot of time does not include those that have been made and repaired. In the new discussion, we point out that there are well described hotspots for SSBs, DSBs and oxidation. Thus, over decades (in humans), it is likely to be somatic changes at hotspot sites that result in a reproducible pattern; the effects of random DSBs forming at other locations would be too small to be detected.

  [Added to discussion]

  "The patterns of transcriptional alterations in HD are somewhat reproducible[60,111-113], which is unlikely to derive from largely random DSBs throughout the genome. However, clustered signatures of single nucleotide variants (SNVs) display recurrent patterns of damage that is shared among neurodegeneration diseases[114,115] at hotspots for SSBs[104,105,116,117], DSBs[118,119], and oxidation[120-122]. Thus, over decades, we envision that DSBR at preferred sites is likely to provide at least some reproducible somatic variants in active genes, while the effects of random DSBs forming at other locations would be too small to be detected"

(9) *I am not sure how to interpret the 10^5 signal for DSBs per cell, but I thought DSBs were highly damaging and even a handful would result in programmed cell death?*

The toxicity of DSBs arose from studies in cancer cells, whose life depends on DNA replication. Any breaks will block replication, and if not repaired will be toxic. However, neurons do not divide, and it is now well recognized that DSBs reversibly form and play roles in gene regulation in the brain. Thus, DSBs are well tolerated in brain cells under normal conditions if the gene is non-essential, redundant, or with functional backup pathways. However, if DSB disrupt transcription of an essential genes, it will lead to death. in HD, DSBS rise and there is an increasing risk of terminating transcription of an essential gene.

- Dileep, V, Tsai, LH. Neuronal enhancers get a break. *Neuron.* **109**, 1766-1768 (2021).
- Madabhushi, R. *et al.* Activity-Induced DNA Breaks Govern the Expression of Neuronal Early Response Genes. *Cell* **161**, 1592-1605 (2015).
- Suberbielle, E. *et al.* Physiologic brain activity causes DNA double-strand breaks in neurons, with exacerbation by amyloid-β. *Nature neuroscience* **16**, 613-621 (2013).
- Sutormin, D. A. *et al.* Diversity and Functions of Type II Topoisomerases. *Acta Naturae* **13**, 59-75 (2021).

- Boutros, S.W, Unni, V. K. & Raber, J. An Adaptive Role for DNA Double-Strand Breaks in Hippocampus-Dependent Learning and Memory. *Int J Mol Sci* **23**, 8352 (2022).
- Konopka, A., Atkin, J,D. The Role of DNA Damage in Neural Plasticity in Physiology and Neurodegeneration. *Front Cell Neurosci.* **16**, 836885 (2022).
- Alt, F.W. and Schwer B. DNA double-strand breaks as drivers of neural genomic change, function, and disease. *DNA Repair (Amst).* **71**,158-163 (2018).
- Pollina, E. A. *et al.* A NPAS4-NuA4 complex couples synaptic activity to DNA repair. *Nature* **614**, 732-741 (2023).
- Min, S., Ji, J.-H., Heo, Y. & Cho, H. Transcriptional regulation and chromatin dynamics at DNA double-strand breaks. *Exp Mol Med* **54**, 1705-1712 (2022).
- Yu, L. *et al.* A novel copper-sensing two-component system for inducing Dsb gene expression in bacteria. *Science Bulletin* **67**, 198-212 (2022).
  Milano, L., Gautam, A,. Caldecott, K.W. DNA damage and transcription stress. *Mol Cell.,* **84,**70-79 (2024).
- Caldecott, K.W. Causes and consequences of DNA single-strand breaks. *Trends Biochem Sci.* **49**, 68-78 (2024).
- Álvarez-Lindo, N., Suárez, T. & de la Rosa, E. J. Exploring the Origin and Physiological Significance of DNA Double Strand Breaks in the Developing Neuroretina. *International journal of molecular sciences* **23**, 6449 (2022).
- Yao, H., Wu, Y., Zhong, Y., Huang, C., Guo, Z., Jin, Y., and Wang, X. Role of c-Fos in DNA damage repair. *J Cell Physiol.* **239**, e31216 (2024).

Minor points

i) Too many obscure abbreviations e.g., STR for striatum saves 5 letters and is confusing given that the HTT repeat is also a simple tandem repeat and that this is the most common use of the STR abbreviation.

ii) Remove redundant acronyms such as SDS-PAGE gels.

We are happy to remove obscure abbreviations according to the reviewer's comments. However, we are uncertain which should be changed. Are you saying STR can be mistaken for HTT and should be written as striatum? Similarly, it is not clear what SDS-PAGE should be changed. There are multiple kinds of protein gels. We appreciate the guidance of the reviewer to make these changes.

Reviewer #2 (Remarks to the Author)

The present manuscript by Polyzos et al. provided strong evidence supporting the role of NHEJ, the dominant DSBR pathway in brain cells, in the development of Huntington's disease (HD) pathogenesis. Multiple studies and reports indicate that somatic CAG repeat expansion in the HTT gene is a significant driver of HD pathogenesis. However, the authors have argued that DSBR deficiency also can independently contribute to disease onset and progression. Using biochemical, cell biological and in vivo mice experiments, it has been shown that mutant huntingtin impaired NHEJ that led to similar transcriptional pathology, as was observed with conventional CAG repeat expansion. This is an important observation and consistent with several recent reports. Over the last few years, it has been shown that impaired PNKP-mediated NHEJ repair is a key factor in polyglutamine diseases, including Spinocerebellar ataxia type 3 (SCA3) and HD (PMIDs: 32205441; 39298485). Studies have convincingly shown that PNKP activity is severely

abrogated in disease cells, mice and patient tissue extracts leading to persistent DSB accumulation in HD, which is consistent with the data presented in this manuscript. Hence, such findings can be discussed in a proper context. Overall, the study provided a deeper understanding of the mechanistic basis of the disease, paving the way for potential treatments of HD, a currently incurable neurodegenerative disorder. Despite the comprehensive nature of the study, some additional clarifications and more details on the methods are needed before it becomes suitable for publication in Nature communications. Studies have convincingly shown that PNKP activity is severely abrogated in disease cells, mice and patient tissue extracts leading to persistent DSB accumulation in HD, which is consistent with the data presented in this manuscript. Hence, such findings can be discussed in a proper context. Overall, the study provided a deeper understanding of the mechanistic basis of the disease, paving the way for potential treatments of HD, a currently incurable neurodegenerative disorder. Despite the comprehensive nature of the study, some additional clarifications and more details on the methods are needed before it becomes suitable for publication in Nature communications.

MSH3 (driver of somatic expansion) is present relatively in a higher amount in striatal neurons; in contrast, glial cells show negligible MSH3 expressions. What is the possible mechanism of CAG repeat expansion in glial cells? Some discussion will be helpful for common readers. For the IP-MS experiment (Fig 3), the authors have used a “non-specific control antibody” to correct the background signal. However, no specific information is provided about this control antibody. This could be clarified. The method section could be more elaborated on providing sufficient detail for readers to understand and potentially replicate the study. Specifically, how the interaction intensities were calculated, and how they were normalized with any background signal? To measure the activity of various DNA repair pathways (NHEJ and HR) the reporter plasmid has been used (Fig 4); however, the design is not very clear. For NHEJ, a blunt ended DSB was used while for HR an overhang-containing DSB was used. Without specific DNA/Amino acid sequence information at the break site, it is not possible to assess potential consequences of repair synthesis. Since this DSBR assay involves expression of GFP protein; hence maintenance of proper GFP sequence would be crucial for successful expression of the protein. Therefore, a schematic with proper sequence description will be very helpful for the common readers to follow the assay system. The authors have selected APE1 as a representative protein involved in BER pathway for the reporter plasmid-based assay. However, oxidized DNA bases are abundant DNA lesions, and their repair is initiated by one of many DNA glycosylases. Moreover, an independent and parallel NEIL-PNKP mediated BER sub-pathway is also operative. Therefore, the diversity of these enzymes and their overlapping substrate specificities make it challenging to select a single representative protein. AP sites are also quite abundant DNA lesions in mammalian genomes. So, we suggest rephrasing the rationale for using THF-containing plasmid substrate. No statistical analysis is provided for Figure 4C. Inhibition of HR is as significant as that of NHEJ, given that NHEJ is the major DSB repair pathway in the brain cells? For in vivo staining of mice brain (Fig. 5E), 53BP1 was used as DSB marker. While 53BP1 is known for its role in NHEJ, assessing phosphorylation status (p53BP1) can provide more specific information. The authors should clarify whether they used phospho-53BP1 or total 53BP1. The in vivo staining experiment is difficult to follow. Though 53BP1 was used as a marker, no quantification data is shown for such analysis. A representative panel at 2 h post-recovery is shown (Fig 5E); it is recommended that the authors display all relevant time points. Finally, the quantification of γH2AX foci in HD mice shows a significant decrease by 4 h post-irradiation, indicating efficient DSB repair, while such repair is not very prominent in WT mice. Some clarification could be provided about this in vivo staining data.Fig. 7-8: Here it should also be clarified whether p53BP1 or total 53BP1 Ab was used for these experiments. The presence of 53BP1 foci in such cells further confirmed that the DNA repair mechanism used in those cells is NHEJ. However, we would like to suggest investigating RAD51 foci, a marker for homologous

recombination (HR), as well. Therefore, by examining both foci formations, researchers can determine the repair pathway being utilized in the cell and will help
to understand the DNA damage response.

Minor comments:

Line 78: reference citation needs to be corrected.

Line 123: "However, MutSβ drives somatic expansion of CAG triplet repeats in knockout mouse crosses": It is better to clarify the term "knockout mouse crosses".

Line 261: Rewrite: "The activity of both HR and NHEJ activity is naturally low in the WT brain".

RESPONSE TO COMMENT IN PARAGRAPH 1: *"Over the last few years, it has been shown that impaired PNKP-mediated NHEJ repair is a key factor in polyglutamine diseases, including Spinocerebellar ataxia type 3 (SCA3) and HD. Studies have convincingly shown that PNKP activity is severely abrogated in disease cells, mice and patient tissue extracts leading to persistent DSB accumulation in HD, which is consistent with the data presented in this manuscript. Hence, such findings can be discussed in a proper context"*

This was an oversight on our part. In the revision, we have taken care to include a greatly expanded discussion of the literature for involvement of PNPK inhibition in SCA3 and HD. Indeed, there is substantial evidence that persistent or complete loss of DNA repair by mutations and polynucleotide kinase/phosphatase (PNKP) in human disease impairs the ability to repair DSBs associated with spinocerebellar ataxia with axonal neuropathy (SCAN1) and ataxia-oculomotor apraxia 1 (AOA1), and spinocerebellar ataxia-3 (SCA3). While in HD, there is no mutation in DNA repair machinery, particularly important, is that a similar finding has been reported for SCA3. In that gene, the end joining activity of PNPK activity is inhibited by the expanded ataxin 3 gene product in SCA3 in mice and patient tissue extracts, also leading to DSB accumulation.

- Chakraborty A, Tapryal N, Venkova T, Mitra J, Vasquez V, Sarker AH, Duarte-Silva S, Huai W, Ashizawa T, Ghosh G, Maciel P, Sarkar PS, Hegde ML, Chen X, Hazra TK. Deficiency in classical nonhomologous end-joining-mediated repair of transcribed genes is linked to SCA3 pathogenesis. Proc Natl Acad Sci U S A. 2020

- Chakraborty A, Sreenivasmurthy SG, Miller W, Huai W, Biswas T, Mandal SM, Boscá L, Krishnan B, Ghosh G, Hazra T. Fructose-2,6-bisphosphate restores DNA repair activity of PNKP and ameliorates neurodegenerative symptoms in Huntington's disease. Proc Natl Acad Sci U S A. 121, e2406308121 (2024).

{Text included in revised discussion--Refs already included}

RESPONSE TO SPECIFIC COMMENTS OF REV 2

(1) *MSH3 (driver of somatic expansion) is present relatively in a higher amount in striatal neurons; in contrast, glial cells show negligible MSH3 expressions. What is the possible mechanism of CAG repeat expansion in glial cells? Some discussion will be helpful for common readers.*

A good point to clarify. In other mouse models, the level of expressed MSH3 determines the degree of expansion, e.g. mice with high genetic backgrounds of MSH3 expand the most (see

refs 69-71). Thus, expansion in glial cells is expected to occur by the same MSH3-driven mechanism as in neurons, but to a lesser extent. This has been measured experimentally (Gonitel, R. et al. DNA instability in postmitotic neurons. *Proc Natl Acad Sci U S A.* 105, 3467–3472 (2008).

[Comment added in results]

(2) *For the IP-MS experiment (Fig 3), the authors have used a "non-specific control antibody" to correct the background signal. However, no specific information is provided about this control antibody. This could be clarified. The method section could be more elaborated on providing sufficient detail for readers to understand and potentially replicate the study. Specifically, how the interaction intensities were calculated, and how they were normalized with any background signal?*

The nonspecific antibody is the mouse anti-Goat IgG bivalent antibody (ThermoScientific cat# 31107) which contains no epitope for proteins. It is common "negative" control antibody. We have included a description of the antibody in the text and legend of Supplementary Fig. 4, and in the methods. An enhanced methods section for the IP-MS experiments is also included in the revision. Basically, the MS analysis was performed and quantified by standard liquid chromatography–tandem mass spectrometry [LC-MS/MS] in the UC Davis proteomics core. The IP products from htt and mhtt expressing cells were measure in independent reactions, and bars represent the MS-MS m/z peak areas after trypsin digestion relative to column standards. These methods were described in methods: Mass spectrometry

[Revision includes these descriptions in the text and methods.]

To measure the activity of various DNA repair pathways (NHEJ and HR) the reporter plasmid has been used (Fig 4); however, the design is not very clear. For NHEJ, a blunt ended DSB was used while for HR an overhang-containing DSB was used. Without specific DNA/Amino acid sequence information at the break site, it is not possible to assess potential consequences of repair synthesis. Since this DSBR assay involves expression of GFP protein; hence maintenance of proper GFP sequence would be crucial for successful expression of the protein. Therefore, a schematic with proper sequence description will be very helpful for the common readers to follow the assay system.

We have revised the text as suggested by the reviewer. Included are (1) an updated Fig. 4 showing the exact DNA sequences around the relevant cleavage site in each reporter plasmid; (2) complete vector maps (Supplemental Fig. 5) and (3) complete sequences of the reporter plasmids provided to GenBank, attached as source data. All reporters were confirmed by sequencing. This information is included in the revised methods. A brief description of the strategy for preparing the reporter plasmids is included in the revised methods, but the methodology has been described in detail in our published work. (PMID 38906885, 34363069, 24757057, 29122935).

[Added] New Fig. 4, New Supplemental Fig. 5 and New Supplemental Fig. 6; Entire GeneBank sequences included in source files].

(3) *The authors have selected APE1 as a representative protein involved in BER pathway for the reporter plasmid-based assay. However, oxidized DNA bases are abundant DNA lesions, and their repair is initiated by one of many DNA glycosylases. Moreover, an independent and parallel NEIL-PNKP mediated BER sub-pathway is also operative. Therefore, the diversity of*

these enzymes and their overlapping substrate specificities make it challenging to select a single representative protein. AP sites are also quite abundant DNA lesions in mammalian genomes. So, we suggest rephrasing the rationale for using THF-containing plasmid substrate.

As suggested by the reviewer, we have rephrased the rationale for the THF BER reactions used. Indeed, BER involves two major subpathways (long and short patch repair), which have overlapping substrate specificities. LP-BER and SP-BER also share components; long patch utilizes a DNA glycosylase, APE 1, pol β, DNA ligase I and FEN1, and a short patch typically includes a DNA glycosylase, APE 1, PNK, polβ. Thus, APE 1 protein was included only as a representative protein to show that the HD brain tissue expressed the major BER machinery (Fig.3). Given overlapping specificities and shared use of some BER enzymes, either pathway could be used to probe BER activity. We chose to measure long patch BER (LP-BER) subpathway, using the THF lesion, as a model of BER activity in the context of intact cells, where all other BER enzymes were present. These details and rephrased rationale have been incorporated in the revised text together with the following references.

[Revision text added to Fig. 4 description]

- Hegde, M., Hazra, T. & Mitra, S. Early steps in the DNA base excision/single-strand interruption repair pathway in mammalian cells. *Cell Res* **18**, 27–47 (2008).

- Prasad R, Shock DD, Beard WA, Wilson SH. Substrate channeling in mammalian base excision repair pathways: passing the baton. J Biol Chem. 2010 285(52):40479-88.

[These refs were added]

(4) (*a) No statistical analysis is provided for Figure 4C. (b) Inhibition of HR is as significant as that of NHEJ, given that NHEJ is the major DSB repair pathway in the brain cells?*

(a) The statistical comparison was not thoroughly described in the results. The relative repair activity in both WT and HD cells was measured by FM-HCR but DSBR activity too low and too variable to determine a statistical difference in genotype. Therefore, significance of DSBR reduction was established using two additional and complementary studies- one in cells (Fig. 5A-C) and one in mouse (Fig. 5D-G).

[We have revised the text to clarify and emphasize this point].

(b) The FM-HCR assays require transfection in glia (which divide). Since the machinery for HR and NHEJ is abundant (Fig. 3), we were able to measure both NHEJ and HR pathway activity using FM-HCR templates. However, in non-dividing neurons, there is no homologous chromosome. Thus, conventional HR is unlikely to be used, and we focused on NHEJ activity.

(5) *For in vivo staining of mice brain (Fig. 5E), 53BP1 was used as DSB marker. While 53BP1 is known for its role in NHEJ, assessing phosphorylation status (p53BP1) can provide more specific information. The authors should clarify whether they used phospho-53BP1 or total 53BP1.*

In the revised methods, we have added a sentence to define total as 53BP1 and p53BP1 as phospho-53BP1. We used only 53BP1 in the analysis. In the text, we refer to the label "53BP1" to specify the form to avoid confusion.

[Included in the revised text and methods]

(6) *The in vivo staining experiment is difficult to follow. Though 53BP1 was used as a marker, no quantification data is shown for such analysis. A representative panel at 2 h post-recovery is shown (Fig 5E); it is recommended that the authors display all relevant time points. Finally, the quantification of γH2AX foci in HD mice shows a significant decrease by 4 h post-irradiation, indicating efficient DSB repair, while such repair is not very prominent in WT mice. Some clarification could be provided about this in vivo staining data.*

In the revised text, we have included new Supplemental Fig. 8, which provides the IF staining for the entire time course for all antibodies used. A slight decrease is also observed in WT at 4 hours. However, the DSB level in WT is much lower; and the WT decrease is proportional. Importantly, elevation of DSBs remains significantly higher in HD cells than in WT at 4 hours, supporting the major point of slower repair in HD cells.

[New Supplemental Fig 8 added showing entire time course]

(7) *Fig. 7-8: Here it should also be clarified whether p53BP1 or total 53BP1 Ab was used for these experiments. The presence of 53BP1 foci in such cells further confirmed that the DNA repair mechanism used in those cells is NHEJ. However, we would like to suggest investigating RAD51 foci, a marker for homologous recombination (HR), as well.*

53BP1, see 6 above)
For RAD51, see comment 5b above. (HR is not used in non-dividing neurons (although their activity can be measured in transfection assays if the protein is expressed).

Minor comments:

Line 78: reference citation needs to be corrected.

We rewrote and completed the cut-off ref in line 78

Line 123: "However, MutSβ drives somatic expansion of CAG triplet repeats in knockout mouse crosses": It is better to clarify the term "knockout mouse crosses".

We have entirely removed "mouse crosses". Since MutSβ drives somatic expansion in humans and in mouse and there is no need to specify the mouse.

""However, MutSβ drives somatic expansion of CAG triplet repeats"

Line 261: Rewrite: "The activity of both HR and NHEJ activity is naturally low in the WT brain".

We have re-written this sentence: "The activity of DSBR was low in WT cells[90] and tended to be further reduced in HD cells (Supplemental Fig. 5A-C)"

Reviewer #3 (Remarks to the Author)

I co-reviewed this manuscript with one of the reviewers who provided the listed reports. This is part of the Nature Communications initiative to facilitate training in peer review and to provide appropriate recognition for Early Career Researchers who co-review manuscripts.

Reviewer 4 (Remarks to the Author):

The central issues of replication and statistical robustness remain unresolved. Biological replication is extremely limited: several key datasets are based on n = 1 (e.g. Figure 6J/K, parts of Figure 4, Supplemental Figure 1) or n = 2 animals (Figures 1–3, Figure 7, Figure 8, Supplemental Figures 2–5). In Figure 5F, dozens of datapoints are drawn from only three mice, inflating technical over biological replication. In addition, figure legends frequently fail to state n clearly, making interpretation difficult. For n = 1, no statistics are valid and p-values should be removed; for n = 2, parametric tests are not appropriate and results should be presented descriptively. Without adequate biological replication, the statistical conclusions remain insecure.

Further methodological concerns also remain. For example, for the FM-HCR assays (Figure 4) lack pathway-specific controls, making it unclear whether the reporters are functioning as intended in these cells. The interpretation of 53BP1 immunofluorescence (Figure 7) as a readout of DNA repair is problematic, since bulk nuclear intensity does not capture the relevant foci dynamics (similarly for gamma-H2Ax). The link to transcriptional dysregulation is still correlative and not demonstrated by rescue experiments.

While the manuscript is clearer and better referenced, the central findings are undermined by insufficient replication, over-representation of technical replicates, and weak mechanistic validation. The story is interesting and timely, but the data in its current form are not sufficiently robust to support the strong conclusions drawn.

Response to Reviewer #4

The response is divided into two sections: a **general response** addressing the main points raised, followed by **point-by-point responses**.

General Response to Reviewer #4

I. Statistical robustness

<u>There are no instances of n = 1 or n = 2 mice in any experiments.</u> The low n values suggested by the reviewer do not correspond to the biological replicates described in the figure legends, nor do they reflect the dozens of technical replicates used in single-cell analyses.

It appears the confusion may arise from a misunderstanding of box plots. Box plots display central tendencies and variability for a pooled set of samples per genotype (original "Tukey" analysis). "WT" and "HD" do not refer to a single animal. The distribution of values for all WT and all HD animals tested are groups per genotype and plotted together in the box plots marked "WT and "HD". Each point in the plot is an individual scored value. For example, Fig. 2C is MSH3- or MSH6 antibody-

stained NeuN(+) neurons (top plot) or NeuN(-) glia (GL) cell (bottom plot) in the striatal tissue sections. The median (the line) shows that MSH3 is expressed equally well per genotype in neurons and is not statistically significant by ANOVA. In Figure 6K, roughly 100 comet tail moments were measured for each of n = 3 animals per genotype, giving a "cloud" of ~300 points per genotype in the box plots marked "WT and "HD". The median value show clearly that the number of comets (DSBs) is higher in the HD group relative to the WT group. We are carefully editing the text and legends to make sure that n is clear for each experiment.

All experiments presented here are statistically robust. The selection of biological replicates (n) in mouse studies is not arbitrary; the choice is grounded on well-established statistical principles. By definition, the number of biological replicates is determined by the expected variability in the experimental outcome. In this study, we-by design-generated a congenic mouse line, ensuring that all animals are genetically identical (clonal)—a process that required years of careful breeding. The great value of using a congenic strain lies in its near-zero inter-animal variability, which (by definition) dramatically reduces the number of animals needed to achieve statistical significance. Importantly, because the genetic background is identical, gene expression profiles are effectively the same across animals. Consequently, n = 3 congenic mice per genotype is sufficient to accurately characterize the biochemical features present in their tissues, such as DNA repair protein expression. By contrast, more animals would be necessary for complex behavioral analyses, where factors like handling, geometry, and physical cues can introduce variability. In our experiments, each biochemical feature observed in one mouse was consistently replicated in the other genetically identical congenic mice (up to six in some cases) across genotypes and ages. Box plots display the full distribution of individual measurements, and statistical significance is rigorously determined using ANOVA. This approach ensures that all conclusions drawn are both statistically sound and biologically meaningful.

The high-density single-cell data in Fig. 5F do not inflate or affect biological n; these are entirely separate parameters and should not be conflated. The number of biological replicates (n) is determined by the expected variability in the outcome (see above). In this study, we by design used congenic strains throughout, in which all animals are genetically identical (clonal), ensuring negligible variation in their biochemical features, such as protein expression. In our case, n = 3 congenic mice per genotype was statistically sufficient to define the biochemical properties measured in their tissues (see below). In contrast, single-cell measurements represent technical replicates that capture the distribution of individual data points, and benefits from high-density sampling. This enhances the robustness of median values and provides precise estimates of variability within each group. This allows a strong test of whether one group differs from another, visualized by the median. Both biological and technical replicates are essential, and this combined approach was appropriate and rigorous in all experiments.

II. Methodology

The methodology for the repair assays used (e.g., antibody staining, FM-HCR, comet assay, radiation-induced foci) are well-established in the DNA repair field. We follow the conventions and controls, which are laid out in the point-by-point response. Indeed, all the methodologies used in this manuscript were previously reviewed and published last year in a companion paper (Polyzos et al Nat Comm, 2024), which is cited. They have not changed and remain valid in this paper.

III. Modifications in the Revised Manuscript

We have submitted a manuscript revision with careful attention to methods and legends, to clarify the experimental design, statistical validity, and methodology while addressing each point raised by Reviewer 4.

1. Rewritten figure legends and main text for clarity, explicitly stating biological and technical replicates. We highlight that “WT” and “HD” Box plots display the full distribution of measurements for all animals tested in each group, not a single animal.
2. Added citations for well-established methodologies.
3. Provided a description of congenic lines in the husbandry section.
4. Added new supplementary information where indicated.

All changes in the revised manuscript are highlighted in red.

POINT-BY-POINT RESPONSE

1. Statistical robustness

Comment: Several datasets reportedly based on n = 1 (Figure 6J/K, parts of Figure 4, Supplemental Figure 1). Several datasets reportedly based on n = 2 *(Figures 1–3, Figure 7, Figure 8, Supplemental Figures 2–5).*

Response: <u>There are no instances of n = 1 or n=2 in the cited figures.</u> The perceived n = 1 likely arises from misinterpretation of the visual representation of pooled data in the box plot. Boxes designated “WT” and “HD” display combined comet data from all animals in the genotype (not one animal).

Figure 6J/K (comet assays)

Response: <u>There are no instances of n = 1 in the cited figures.</u>

Fig, 6J follow comet analysis—direct measures of DNA breaks in dispersed cells from brain tissue of multiple animals. The DSBs are identified after resolution on non-denaturing gels to reveal the broken DNA seen as trailing behind the rest of the genome DNA. The comet tails and heads (bright DNA intensity) are quantified and digitized by instrument software. (Fig. 6J) is a schematic of the method and examples of the comets from WT and HD in two brain regions. It is possible that the example comets were taken as the only samples that were measured, but they are examples and do not reflect the actual number measured (which were hundreds).

(Fig. 6K) There are no instances of n = 1. Misinterpretation may arise from the visual representation of pooled data in the box. The boxes labeled “WT” and “HD” are not single animals. The single-cell data of comet tail moments from all n = 3 animals (from J) per genotype are pooled and displayed together in the same box. The box plot represents 50–100 comets per animal, which are plotted together for each genotype (up to 300 per genotype) in boxes labeled “WT” and “HD”. This classic

representation is done to increase the data density for a more robust comparison of the distribution. The median value of the data shows that the DSB in HD are higher than in WT. Collectively, the comets are significant and the DSBs rise in HD animals with age.

Modification. To emphasize this point, we have included a new Supplement 10D,E. Shown are the comets visualized on the non-denaturing gels from three individual animals (in 10D). We have included additional amplified comets from these gels (10E). The comet assay mirrors the DSB levels observed by both γH2AX and 53BP1 antibody results (in Fig. 6DH,I). Collectively, we find that DSBs rise in HD animals with age by any of these detection methods using a meaning number of animals.

Figure 4 (FM-HCR assays, cell culture)

There was no n-=1. However, Figure 4 describes primary cell culture transfection experiments for five repair reporters in cells isolated from the CBL or STR of either WT or HD. Cell transfections were reported as independent technical replicates: n = 6 for CBL, n = 5–12 for STR. The results are provided as source data on the journal website. The cultures themselves were derived from multiple embryos (4–8 per genotype) and 6-12 pregnant females (depending on litter size) per culture. There is no "n=1" here.

Modification: In the original submission, n for technical replicates was included in the legends and the embryo pooling was described in the cell isolation methods. In the revision, we have combined the statements of technical replicates and embryo collection together in the same legend in case it was confusing. The n technical replicate values are provided on the journal website as source data.

Supplemental Figure 1 (Genescans)

The comment differs from what is shown in the Figure. Paired scans of four animals are shown, and the individual animals are indicated by their IDs: 668, 621, 162, 163).

Modification: Enlarged animal IDs for clarity.

Reviewer Comment 2: Several datasets reportedly based on n = 2 *(Figures 1–3, Figure 7, Figure 8, Supplemental Figures 2–5).*

Response: No instances of n = 2 in any Figure

Figures 1-3 are quantified data for MSH2, MSH3 and MSH6 by western analysis or (in Fig. 3) quantified data for other DNA repair pathways. Supplemental Figures 2 and 3 are gel representations of the primary data).

Confusion likely arose from the gels in Supplemental Fig. 2. Western analysis of the DNA repair machinery (in Fig, 1 and Fig 3) was evaluated from n=3 samples. *Due to well limitation, however, only two of the three samples could be loaded side by side on the same gel, indicated by the numbers 1 and 2 (see Supplemental Fig. 2A). Data for the third sample was provided in the source data on the journal website.* The same is true for the other DNA repair proteins shown Fig. 3 and in Supplemental 3. *Three samples were used in all analysis.* There were NO significant differences between genotypes.

Modification. We better highlight-in the text and in the legends that the third protein sample was resolved on a different gel. We include the protein quantification for the third protein sample in the source data on the journal website.

Figures 2, 7, 8. Misinterpretation may have arisen from the visual representation of pooled data in the box. Boxes designated "WT" and "HD" display combined data from all animals in genotype (not two animals), as described below.

Figure 2:

This is a single-cell experiment showing the cell distribution of MSH3 or MSH6 expression in individual neurons and glia in brain tissue sections. Box plots display (~50-100 single cells) pooled from all n=3 animals per genotype and displayed together in boxes plots labeled "WT" or "HD", where each point in the box plot is an individual scored MSH3 or MSH6 stained cell. Three were no significant genotype-dependent changes,

Modification: In the revision, the legends have been revised for clarity. To help the reader, we provide Fig. 2 A-C, as examples of antibody staining to show that MSH3 (or MSH6) and NeuN co-stain (i.e., neurons) in the tissue fields. Additional images are provided in single cells staining in Supplemental Fig, 2A-C.

Figure 7

No instances of n = 2. The results in Fig 7 measure DSBs from 53BP1 staining in single cells in four genotypes: C57Bl6, MSH3(-/-). zQ175 and zQ175/MSH3(-/-) at two ages (3 and 6 months). However, there are two comparison groups. C57Bl6 ("WT") is the genetic background control for zQ175(HD) and MSH3(-/-) ("WT") is the genetic background controls for zQ175/MSH3(-/-)("HD"). Thus, the perceived n = 2 likely arises from misinterpretation of the visual presentation of pooled data in the box plot.

Modification. We have revised Fig. 7 to make sure the labeling is clear. We show paired box plots where C57Bl6 ("WT") and zQ175(HD) are white bars while MSH3(-/-) ("WT") and zQ175/MSH3(-/-)("HD") are in black bars. We have clarified biological n. For each genotype, the box plots show 53BP1 stained cells (~50 foci per animal), which are pooled from (n=3) test animals per genotype and displayed together as paired box plots for each comparison. For the antibody images, we have separated them and added "WT" and "HD" in the figure to mark which strains are WT and which are HD in the two comparisons.

Figure 8:

The is no instance of n=2.

We provide two complementary experiments to assess DSBs and their inhibition by XJB-5-131: (1) γH2AX antibody detection ± XJB-5-131 (shown in 8F) and (2) comet assay (±XJB-5-131) (shown in 8G). In 8F and 8G, there are three groups measured: WT, (Vh), HD(Vh) and HD +XJB-5-131. Reversibility of DSBs across methods mirror each other.

Confusion may have arisen since data are collected from two genotypes, two brain regions and in two cell types. However, the meaning of the box plots is the same in every experiment: (Fig. 8F)

The box plots for γH2AX antibody detection includes stained cells (~50 foci per animal) pooled from (n=3) test animals for each genotype and displayed together in boxes mark either "WT", "HD" or HD+XJB-5-131". Each point in the plot is an individual scored γH2AX stained cells. The same analysis was performed in the CBL and STR, in both NeuN(+) and NeuN(-) glia of the STR and CBL and are specified.

We backed-up the γH2AX analysis with comet assays (Fig. 8G). We matched DSBs measured in striatal neurons by γH2AX antibody detection (in Fig. 8F, STR, Neu) to comets in the same tissues (Fig. 8G). For comet assays, the box plots include (~100 comets per animal) pooled from all (n=3) animals per group: "WT(Vh)", HD(Vh)" or HD+XJB-5-131". Each point in the plot is an individual scored comet. To show comet reversibility more visually, we have included a new Supplemental Fig. 13 to show images of XJB-5-131-reversible comets from "WT(Vh)", HD(Vh)" or HD+XJB-5-131 groups directly.

Modification. We rewritten the legend and text for clarification. We have included a new Supplemental Fig. 13. Shown are side-by-side amplified images of comets for "WT(Vh)", HD(Vh)" or HD+XJB-5-131". Since comets are broken DNA, there is obvious reduction of tail moments in the presence of XJB-5-131, showing clear reversibility of DSBs.

Comment: Figure 5F reportedly inflates technical over biological replicates.

Response: (see general comments) High-density single-cell data do not inflate or influence biological n. They are separate parameters and should not be misunderstood. The number of biological replicates (n) is determined based on the expected differences in the outcome (**discussed in the general section), In our congenic strain, there are no expected differences in outcomes among clonal animals. Thus, n=3 congenic mice of each genotype was statistically sufficient to define a biochemical feature such as expression of gene products. The single cell comet measurements (technical replicates) define the point distribution of values, which benefits from high density information and ensure robustness of the median values. ANOVA indicates a robust probability that the mean number of DSBs is higher in HD than for WT.

---

2. Methodology.

Comment: *Further methodological concerns also remain. For example, for the FM-HCR assays (Figure 4) lack pathway-specific controls, making it unclear whether the reporters are functioning as intended in these cells.*

The assays used (e.g., antibody staining, FM-HCR, comet assay, radiation-induced foci) are well-established in the DNA repair field. We are experts in using these techniques, and throughout, we have implemented established methods and controls which have been previously published and thoroughly validated over a decade.

FM-HCR assays (Figure 4)

The FM-HCR assay is not new. It was invented by co-author Nagal[1-3] The well-established vectors and controls are described in the detailed methods. Indeed, over the years, the controls have been "baked into" the FM-HCR cocktails themselves and the results are automated (i.e., automatically

normalized in the flow cytometry output of each experiment). The assay and controls work, as follows. The fluorescent reporter assay monitors the recovery of a fluorescent signal from a template harboring a preferred substrate lesion for a particular repair pathway. For example, if a DSB exists in the coding sequence of the reporter gene, it cannot be expressed unless the DSB is repaired by DSBR pathway. (The site of the DSB is indicated in Fig. 4B). Thus, the assay is performed, as follows: Two cocktails are prepared. The first contains the set of damaged plasmids, i.e., the lesioned templates that are the substrates for repair. In parallel, a second cocktail is generated which contains the set of "undamaged" templates or intact reported gene plasmids, which retain full gene activity. Both cocktails are prepared and transfected separately but include the same transfection efficiency control. Because the lesion disrupts the coding sequence and its expression, recovery of reporter expression serves as a measure of pathway-specific lesion repair. Repair activity is expressed as % reporter expression, calculated from the ratio of activity of the damaged (+lesion)(cocktail 1) and undamaged (no lesion)(cocktail 2) plasmid reporters. Each plasmid has been characterized by us and others in over decade of published work, for the effects of the lesion on repair, transcription, and the chemistry of the lesion and their functionality. See for example.

- Margulies CM, et al., Multiplexed DNA repair assays for multiple lesions and multiple doses via transcription inhibition and transcriptional mutagenesis;
- Nagel ZD et al., Fluorescent reporter assays provide direct, accurate, quantitative measurements of MGMT status in human cells. PloS one. 2019;14(2):e0208341).
- Piett CG, Pecen TJ, Laverty DJ, Nagel ZD. Large-scale preparation of fluorescence multiplex host cell reactivation (FM-HCR) reporters. Nat Protoc. 2021;16(9):4265-98.

For clarity, we have re-written the legnds to Figure 4 legnd in the revised text to inlcude a more detailed description of the method in part A.

Fig. 4: BER, NER/TCR, and MMR activities are not genotype dependent.

(A) Schematic of the fluorescence multiplex host cell reactivation (FM-HCR) assay (as described in the text). FM-HCR measures DNA repair activity in cells using a set of reporter plasmids, each harboring specific lesions corresponding to one of the five major DNA repair pathways. *Cocktail A* contains damaged plasmid reporters for all five DNA repair pathways, whereas *Cocktail B* contains a matched set of fully active, undamaged reporter controls (no lesion). Both cocktails are prepared and transfected separately but include the same transfection efficiency control. Because the lesion disrupts the coding sequence and its expression, recovery of reporter expression serves as a measure of pathway-specific lesion repair. Repair activity is expressed as % reporter expression, calculated from the ratio of activity between damaged (+lesion) and undamaged (no lesion) plasmid reporters. The overall HD/WT activity ratios for each repair pathway are shown in Supplemental Fig. 5, and full assay details are described in the *Methods*. (B) Schematic diagram of the color-coded reporter plasmids used to assess the activities of the five indicated pathways: NHEJ (blue), HR (red), MMR (orange), NER (purple), and BER (green). The lesion locations and nucleotide sequences surrounding each lesion site are shown above the reporter. For NHEJ and HR reporters, the vertical bar ("|") indicates the cleavage site that generates the double-strand break (DSB). Full vector schematics are shown in Supplemental Fig. 6A,B. (C) Quantification of % reporter activity for the five DNA repair pathways in primary glial cultures derived from WT and HD animals in the cerebellum (CBL) and striatum (STR). HD/WT expression ratios are provided in Supplemental Fig. 5. Each glial culture was generated from dissected tissue of 4–8 P1–P3 pups (see *Methods*), requiring 3–12 pregnant females per genotype, depending on litter size. Repair activities were confirmed in independent transfection experiments (n = 6 for CBL and n = 5–12 for STR for each set of five reporters per genotype). Source data are provided. All repair pathways were active, with

no apparent genotype-dependent differences in activity. Statistical analysis using one-way ANOVA indicated some significant regional differences ($P < 0.01$).

The plasmids work as expected (see 1-22 below). The properties and suitability of our reporters have also been validated against most known DNA repair assays and extensive testing is provide in numerous papers (1-22). Testing includes cell lines with genetically defined defects in the target reporter pathway, biochemical assays for direct reversal of alkylation damage [7], analysis of base excision repair gene expression [3], reporter systems for double strand break repair that are integrated into genomic DNA [8], immunohistochemical analysis of foci that are associated with double strand breaks [9], and many genetic models of DNA repair deficiency caused by somatic mutations in nucleotide excision repair genes [10], integrated reporter systems for microsatellite instability associated with mismatch repair deficiency[11]. Our FM-HCR assay has been widely adapted by others to identify DNA repair mechanism underlying therapy resistance in cancer cell lines[12-15], determine the effects of nanomaterials on genome integrity [16], establish functional markers of synthetic lethal interactions[17], determine the functional consequences of deleting chromatin remodeling proteins that are involved in genome maintenance[18], confirm the specificity of small molecule inhibitors of DNA repair enzymes [8, 19], determine the activity of large numbers of DNA repair protein variants in multiple DNA repair pathways [10, 20] and to measure human variation in DNA repair [21, 22].

1. Piett CG, Pecen TJ, Laverty DJ, Nagel ZD. Large-scale preparation of fluorescence multiplex host cell reactivation (FM-HCR) reporters. Nat Protoc. 2021;16(9):4265-98. Epub 2021/08/08.
2. Nagal, ZD, Margulies CM, Chaim IA, McRee SK, Mazzucato P, Ahmad A, Abo RP, Butty VL, Forget AL, Samson LD. Multiplexed DNA repair assays for multiple lesions and multiple doses via transcription inhibition and transcriptional mutagenesis. Proceedings of the National Academy of Sciences of the United States of America. 2014;111(18):E1823-32. Epub 4/22/14.
3. Chaim IA, Nagel ZD, Jordan JJ, Mazzucato P, Ngo LP, Samson LD. In vivo measurements of interindividual differences in DNA glycosylases and APE1 activities. Proc Natl Acad Sci U S A. 2017;114(48):E10379-E\10.1073/pnas.1712032114.
4. Athas WF, Hedayati MA, Matanoski GM, Farmer ER, Grossman L. Development and field-test validation of an assay for DNA repair in circulating human lymphocytes. Cancer Res. 1991;51(21):5786-93.
5. Qiao Y, Spitz MR, Guo Z, Hadeyati M, Grossman L, Kraemer KH, Wei Q. Rapid assessment of repair of ultraviolet DNA damage with a modified host-cell reactivation assay using a luciferase reporter gene and correlation with polymorphisms of DNA repair genes in normal human lymphocytes. Mutat Res. 2002;509(1-2):165-74..
6. Qiao Y, Spitz MR, Shen H, Guo Z, Shete S, Hedayati M, Grossman L, Mohrenweiser H, Wei Q. Modulation of repair of ultraviolet damage in the host-cell eactivation assay by polymorphic XPC and XPD/ERCC2 genotypes. Carcinogenesis. 2002;23(2):295-9.
7. Nagel ZD, Beharry AA, Mazzucato P, Kitange GJ, Sarkaria JN, Kool ET, Samson LD. Fluorescent reporter assays provide direct, accurate, quantitative measurements of MGMT status in human cells. PloS one. 2019;14(2):e0208341.
8. Laverty DJ, Gupta SK, Bradshaw GA, Hunter AS, Carlson BL, Calmo NM, Chen J, Tian S, Sarkaria JN, Nagel ZD. ATM inhibition exploits checkpoint defects and ATM-dependent double strand break repair in TP53-mutant glioblastoma. Nature communications. 2024;15(1):5294. Epub 20240621.

9. Vassel FM, Laverty DJ, Bian K, Piett CG, Hemann MT, Walker GC, Nagel ZD. REV7 Monomer Is Unable to Participate in Double Strand Break Repair and Translesion Synthesis but Suppresses Mitotic Errors. Int J Mol Sci. 2023;24(21).
10. Blee AM, Li B, Pecen T, Meiler J, Nagel ZD, Capra JA, Chazin WJ. An Active Learning Framework Improves Tumor Variant Interpretation. Cancer Res. 2022;82(15):2704-15.
11. Rona G, Miwatani-Minter B, Zhang Q, Goldberg HV, Kerzhnerman MA, Howard JB, Simoneschi D, Lane E, Hobbs JW, Sassani E, Wang AA, Keegan S, Laverty DJ, Piett CG, Pongor LS, Xu ML, Andrade J, Thomas A, Sicinski P, Askenazi M, Ueberheide B, Fenyö D, Nagel ZD, Pagano M. D-type cyclins regulate DNA mismatch repair in the G1 and S phases of the cell cycle, maintaining genome stability. bioRxiv. 2024. Epub 20240113.
12. Xiao AY, Maynard MR, Piett CG, Nagel ZD, Alexander JS, Kevil CG, Berridge MV, Pattillo CB, Rosen LR, Miriyala S, Harrison L. Sodium sulfide selectively induces oxidative tress, DNA damage, and mitochondrial dysfunction and radiosensitizes glioblastoma (GBM) cells. Redox biology. 2019;26:101220. Epub 2019/06/09.
13. Shen J, Ju Z, Zhao W, Wang L, Peng Y, Ge Z, Nagel ZD, Zou J, Wang C, Kapoor P, Ma X, Ma D, Liang J, Song S, Liu J, Samson LD, Ajani JA, Li GM, Liang H, Shen X, Mills GB, Peng G. ARID1A deficiency promotes mutability and potentiates therapeutic antitumor immunity unleashed by immune checkpoint blockade. Nature medicine. 2018;24(5):556-62. Epub 2018/05/08.
14. Russo M, Crisafulli G, Sogari A, Reilly NM, Arena S, Lamba S, Bartolini A, Amodio V, Magrì A, Novara L, Sarotto I, Nagel ZD, Piett CG, Amatu A, Sartore-Bianchi A, Siena S, Bertotti A, Trusolino L, Corigliano M, Gherardi M, Lagomarsino MC, Di Nicolantonio F, Bardelli A. Adaptive mutability of colorectal cancers in response to targeted therapies. Science (New York, NY). 2019;366(6472):1473-80.
15. Lee KJ, Piett CG, Andrews JF, Mann E, Nagel ZD, Gassman NR. Defective base excision repair in the response to DNA damaging agents in triple negative breast cancer. \PloS one. 2019;14(10):e0223725. Epub 20191009.
16. Toprani SM, Bitounis D, Huang Q, Oliveira N, Ng KW, Tay CY, Nagel ZD, Demokritou P. High-Throughput Screening Platform for Nanoparticle-Mediated Alterations of DNA Repair Capacity. ACS Nano. 2021;15(3):4728-46. Epub 20210312.
17. Chan EM, Shibue T, McFarland JM, Gaeta B, Ghandi M, Dumont N, Gonzalez A, McPartlan JS, Li T, Zhang Y, Bin Liu J, Lazaro JB, Gu P, Piett CG, Apffel A, Ali SO, Deasy R, Keskula P, Ng RWS, Roberts EA, Reznichenko E, Leung L, Alimova M, Schenone M, Islam M, Maruvka YE, Liu Y, Roper J, Raghavan S, Giannakis M, Tseng YY, Nagel ZD, D'Andrea A, Root DE, Boehm JS, Getz G, Chang S, Golub TR,Tsherniak A, Vazquez F, Bass AJ. WRN helicase is a synthetic lethal target in microsatellite unstable cancers. Nature. 2019;568(7753):551-6. Epub 20190410.
18. Moore S, Berger ND, Luijsterburg MS, Piett CG, Stanley FKT, Schräder CU, Fang S, Chan JA, Schriemer DC, Nagel ZD, van Attikum H, Goodarzi AA. The CHD6 chromatin remodeler is an oxidative DNA damage response factor. Nature communications. 2019;10(1):241. Epub 2019/01/18. doi: 10.1038/s41467-018-08111-y.
19 Nguyen MT, Moiani D, Ahmed Z, Arvai AS, Namjoshi S, Shin DS, Fedorov Y, Selvik EJ, Jones DE, Pink J, Yan Y, Laverty DJ, Nagel ZD, Tainer JA, Gerson SL. An effective human uracil-DNA glycosylase inhibitor targets the open pre-catalytic active site conformation. Prog Biophys Mol Biol. 2021;163:143-59.
20. Cheong A, Fisher A, Bashyam A, Forget A, Peters R, Nagel ZD. Identifying active and inhibitor-resistant MGMT variants for gene therapy. Am J Hum Genet. 2025;112(6):1430-46. Epub 20250520.

21. Zhai T, Mazzucato P, Ricciardi C, Christiani DC, Liang L, Samson LD, Chaim IA, Nagel ZD. Comprehensive Measurement of Inter-Individual Variation in DNA Repair Capacity in Healthy Individuals. medRxiv. 2025. E
22. Toprani SM, Mordukhovich I, McNeely E, Nagel ZD. Suppressed DNA repair capacity in f light attendants after air travel. Sci Rep. 2025;15(1):16513. Epub 20250513. d=å

53BP1 immunofluorescence (Figure 5)

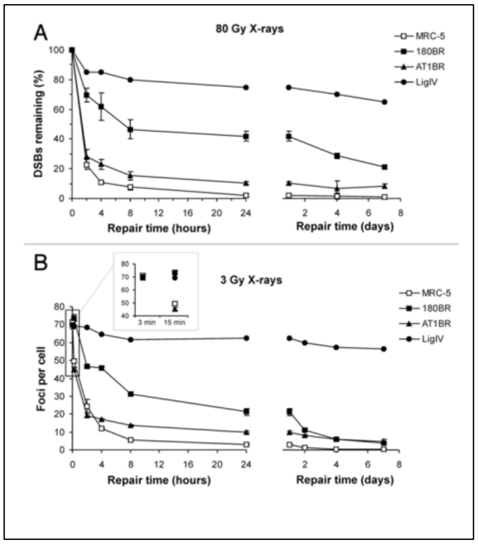

Figure 3. The rate of loss of γH2AX foci correlates with the physical joining of DSBs assessed by PFGE. Cells were grown to confluency, and DSBs in irradiated cells at 3 Gy were measured and analysed by PFGE (A) or with the γH2AX foci assay (B). Wild-type human fibroblasts (MRC-5) exhibit a fast repair component during the first 2–4 h and a second much slower component during the following hours.

Comment. *The interpretation of 53BP1 immunofluorescence (Figure 7) as a readout of DNA repair is problematic, since bulk nuclear intensity does not capture the relevant foci dynamics (similarly for gamma-H2Ax).*

The antibody quantification methodology we use is the standard convention, which has been long established for reliable and robust DSBs detection after radiation damage. As discussed below, the differences in radiation-induced foci (RIF) is commonly visualized and quantified by fluorescent microscopy, in fixed samples, at discrete time points after IR.

Seminal works, described below, form the basis for the established measurement methodology:

Singh, P., et al. (2018). "Radiation dosimetry and repair kinetics of DNA damage foci in mouse pachytene spermatocyte and round spermatid stages." Mutagenesis 33(3): 231-239.

Schultz and Halazonetis. p53 Binding Protein 1 (53BP1) Is an Early Participant in the Cellular Response to DNA Double-Strand Breaks. The Journal of Cell Biology, Volume 151, Number 7, December 25, 2000 1381–1390.

As shown in classic and fundamental experiment of Jeggo and co-workers (Singh, 2018)(Fig. 3 to the left), the time course of foci formation defines the conditions under which foci detection is robust. She (and others) established that loss of γH2AX foci after radiation damage occurs with two-component kinetics (Fig. 3). Cells exhibit a fast repair component during the first 2–4 h and a second much slower component during the following hours and days **(Fig. 3A, left).** While the magnitude of the antibody signal depends on the distinct repair capacity, which may differ among cell lines, kinetics of foci formation are similar (Fig. 3A, left). Moreover, the yield of γ-H2AX foci is linearly related to dose over a very wide dose range (see A and B). The classic work by Halazonetis demonstrated that 53BP1 and γ-H2AX foci co-localize at DSB induced by radiation and showed similar kinetic patterns. Because the recruitment of these two markers to DSB have similar kinetic patterns, differences in repair are reliably measured between two different cell lines/tissues and compared at the same time point. In our cell experiments, radiation-induced foci (RIF) damage was detected by antibodies immediately after exposure, the first phase of repair peaked around 1-2 hours, and decay continues over 24 hours post irradiation. The kinetics of foci dynamics replicate those of Jeggo and others.

Michael Lassmann et al. In Vivo Formation of γ-H2AX and 53BP1 DNA Repair Foci in Blood Cells After Radioiodine Therapy of Differentiated Thyroid CancerNucl Med 2010; 51:1318–1325

The same parameters apply *in vivo.* Lassmann et al., based on *in vivo* irradiation of humans, concluded that after whole-body irradiation, the γ-H2AX and 53BP1 foci manifest a high number of foci, as detected by antibodies shortly (at 2 h) after irradiation (similar to cell experiments); the foci count subsequently decreased over time but tended to remain elevated above baseline levels for at least 144 h after therapy. As in cells, there was a clear and reliable dose–response relationship between the absorbed dose and number of induced foci within individual patients. Thus, the detection of radiation-induced foci (RIF) by γ-H2AX and 53BP1 antibody quantification that we use is a well suited method to analyze DSBs after radiation exposure *in vivo*. We tested DSB foci formation in WT and *HdhQ(150/150)* mice by exposing adult animals (40-50 wks) to (5Gy) (Fig. 5D). The animals were sacrificed immediately after irradiation or at 2 or 4 hours post irradiation, at the maximum of the *in vivo* foci formation observed by Lassmann.

Our experiments demonstrate that DSBs induced by radiation in HD animals are repaired slowly compared to WT animals both *in vivo* and *in vitro.* Importantly, we have supported this conclusion with a direct measure of DNA breaks in ”comet assay”, as a third assay. All *three analyses with very different approaches yield the same answer and back up the conclusions.*

Penninckx, S., et al. (2021). "Quantification of radiation-induced DNA double strand break repair foci to evaluate and predict biological responses to ionizing radiation." NAR Cancer 3(4): zcab046.

For the reviewer’s reference, we highlight additional papers and reviews which describe the most robust and common methodological approaches to quantifying DSB formation by antibodies. As discussed, DSBs is typically quantified either by counting foci or integrating the fluorescent intensity most commonly in fixed samples, at discrete time points. The number of foci per nucleus correlates with the number of DSBs (especially at lower damage levels).

- Nakamura A, Sedelnikova OA, Redon C, Pilch DR, Bonner WM. **Techniques for γ-H2AX detection** (book chapter / methods; 2006).
- Rogakou EP, Pilch DR, Orr AH, Ivanova VS, Bonner WM. DNA double-stranded breaks induce histone H2AX phosphorylation on serine 139. J Biol Chem. 1998 Mar 6;273(10):5858-68.
- Garty G, Chen Y, Salerno A, Turner H, Zhang J, Lyulko O, Bertucci A, Xu Y, Wang H, Simaan N, Randers-Pehrson G, Yao YL, Amundson SA, Brenner DJ. The RABIT: a rapid automated biodosimetry tool for radiological triage. Health Phys. 2010 Feb;98(2):209-17.
- Gallant, K., Bektas, A., Kaileh, M. *et al.* HiIDDD: a high-throughput imaging pipeline for the quantitative detection of DNA damage in primary human immune cells. *Sci Rep* **12**, 6335 (2022).
- Markova, E., et al. (2007). "Kinetics and dose-response of residual 53BP1/gamma-H2AX foci: co-localization, relationship with DSB repair and clonogenic survival." Int J Radiat Biol 83(5): 319-329.
- Kleiner, R. E., et al. (2015). "Chemical proteomics reveals a gammaH2AX-53BP1 interaction in the DNA damage response." Nat Chem Biol 11(10): 807-814.
- Bonner WM, Redon CE, Dickey JS, et al. γH2AX and cancer. Nat Rev Cancer. 2008;8(12):957–967.

- Ivashkevich AN, Martin OA, Smith AJ, Redon CE, Bonner WM, Martin RF, Lobachevsky PN. γH2AX foci as a measure of DNA damage: a computational approach to automatic analysis. Mutat Res. 2011 Jun 3;711(1-2):49-60.

Transcriptional dysfunction and DSBs (Figure 8)

Comment. *The link to transcriptional dysregulation is still correlative and not demonstrated by rescue experiments.*

The conclusions of the paper do not hinge on a correlation between transcriptional dysregulation and DSBs. In this work, we present a testable model in which DSBs, and somatic expansion contribute independently as disease drivers. Thus, the goal of these two reversibility experiments was to test whether the DSBs and somatic expansion have separable effects in promoting disease pathology, one of the major conclusions of the paper.

Two Rescue experiments:

(Experiment 1) Pharmacological reversibility: XJB-5-131 suppresses DSBs and toxicity without substantial effects on somatic expansion.

(Experiment 2) Genetic reversibility: Loss of MSH3 attenuates somatic expansion but does not alter the transcriptional dysregulation or the DSBs which accompany transcriptional dysregulation

Conclusion: These two experiments demonstrate that DSBs and somatic expansion contribute independently to neurotoxicity. We conclude that somatic expansion and DSBs act as two separable processes at potentially distinct stages of the disease. The exact mechanistic relationship between transcription and DSB is a biological question, which will require further analysis. The relationship is presented as a discussion point.

In discussion:

"*Transcriptional dysfunction is a prominent feature of neuropathology in both HD patients and in mouse models[60,111-113]. Whether NHEJ-mediated nucleotide variants and DSBs drive transcriptional dysfunction in the genomes of HD patients remains to be validated. However, our results from "separation of function" mice confirm that DSBs accumulate together with progressive transcriptional dysfunction in z175Q/MSH3(-/-) animals that cannot expand their alleles somatically (Fig. 7).*"…..

Reviewer #4 (Remarks to the Author):

As I began to reassess the manuscript point-by-point, I noted that in their rebuttal, the authors referred to the source data files, which I had not scrutinised in detail during the first round, trusting that the figure representations accurately reflected the underlying data. Upon reviewing these files now, I have identified major discrepancies between the source data values, the plotted figure outputs, the sample labelling (including timepoints), and the described normalisation procedures.

I would strongly recommend that the authors carefully re-examine all quantitative results presented in the manuscript — including raw data, figure annotations, plotted values, normalisation procedures, statistical treatment, and sample metadata — to ensure full internal consistency and traceability. (**1)** For example, the source data file provided for Figures 1–3 uses timepoints labelled as 10–11 weeks and 75–76 weeks, whereas Figure 1 is labelled as 7–10 weeks and 70–90 weeks (Figure 2 labels it correctly for the two datapoints where blots are shown). **(2)** In addition, the third "replicate" used for quantification in some cases originates from 19–20 weeks, which is biologically not part of either of the two age groups depicted and therefore should not be used as a biological replicate. If included, this would artificially expand the age range and potentially confound the results. To maintain biological consistency, the labelling should more accurately reflect what was actually used (e.g., 10–20 weeks vs 75–76 weeks), or the dataset should be corrected to include only valid age-matched replicates. **(3)** Additionally, the quantification appears to be normalised only to WT without applying loading control correction, despite GAPDH bands being shown in some supplementary blots (no blots shown for the third replicate). The source data file does not contain loading-control–adjusted numerical values, nor any indication of background subtraction or target/loading ratios. As a result, the plotted values cannot be fully reconciled with the raw data shown in the blots. (**4)** There are also cases where the numerical data provided DOES NOT match the plotted values. For instance, for MSH3 at 10–11 weeks, the source data report:

CBL STR

	WT	HD
Rep 1	1 0.9917	0.3170 0.2735
Rep 2	1 1.0304	0.3149 0.2185

However, in the figure, HD CBL is shown close to 2.0, which is not supported by the reported values (0.99 and 1.03). Similar discrepancies appear for XPA: the plots for 10-week and 75-week XPA data look nearly identical, yet the source data file reports clearly different numerical values for those timepoints:

10 weeks (source data):

CBL STR
WT HD
Rep 1 1 2.0895 0.9757 1.6014
Rep 2 1 2.8115 0.9282 1.3066

75 weeks (source data):

CBL STR
Rep 1 1 1.2277 0.3906 0.8229
Rep 2 1 2.2005 0.4764 0.7493

Yet, the plotted bars appear nearly the same across both ages, for example, in STR-HD at 10 weeks, the values are about 1.1 and 2.2.

Furthermore, some error bars are truncated (including datapoints, for example, MRE11 at 10 weeks), axis labels appear to have been manually edited (with earlier labels faintly visible beneath), and y-axis ranges are inconsistently adjusted across panels, limiting reliable comparison across conditions.

Considering these various issues, verifying the derivation of the final plotted data is challenging. At this point, I recommend that the authors thoroughly review all data processing steps, including normalisation procedures, figure plotting, and labelling, to verify that the raw data, source files, and visual representations are fully consistent and traceable. I offer these comments in a constructive spirit, recognising the valuable biological questions addressed. Nonetheless, improving clarity, consistency between raw and plotted data, and methodological transparency would significantly strengthen the manuscript and improve its reproducibility. Due to these fundamental concerns, I have not assessed the rest of the manuscript in detail, as these issues should be resolved first.

Response to reviewer 4 comments.

We are hopeful for a fresh start in 2026 and appreciate Reviewer's stated commitment to a fair review. We fully agree that a rigorous process requires clearly presented, well-analyzed data as well as a good understanding of stated hypotheses, experiments, and methods. Towards this end, we have revisited the entire manuscript with attention to clarity and hope the new version is improved. Since minor edits were made to the text and Figures, it will be important to use this new version for the review.

We numbered the comments for easy reference to the points raised.

(1) The reviewer raised a valid concern regarding the source data files, and we are grateful for this feedback. We identified an error stemming from a student re-running western blots and plotting some values that were inconsistent with earlier source file. We have corrected them, re-plotted Figures 1 and 3 so that blots, plots, and source data are fully aligned, removed any error bar truncation issues, and reviewed the manuscript to improve clarity throughout. None of the methods used in the manuscript are new and citations are provided. However, we have rewritten the methods section to include significant detail so that the information is readily available and easy to access during the review

(2) The reviewer raises questions about reproducibility due to inclusion of a single 19–20-week mouse in Fig. 3. While this age group is distinct from the other two animals, reproducibility in this experiment refers to the presence or absence of specific proteins among samples, i.e., is the protein present in the samples measured. Each age group represents an independent test, as protein bands are observed in all animals regardless of age. However, importantly, the data from the 19–20-week animals were not averaged with other groups or included in plotted intensities. Only intensities from young and old groups (samples 1 and 2 at the same ages) were used. The text and source files have been revised to clarify this explicitly.

We have re-organized the source data to highlight this point. The source data for all proteins are now tabulated in a single file. New Fig. 1 and Fig. 3 plots have been redrawn using only raw intensity data, as suggested by the reviewer. Added is a color code where (19,20wk) is "greyed-out" to indicate that-while it was used to show proteins were present in the sample-the intensities were not used in plotting. We added notation to indicate that it was not used in the plots. We have added the uncropped gels for GADPH antibody staining in sample 3. The calculation for n used in the western is provided in (4).

(3) The reviewer's comments on normalization applies to quantitative analyses but not qualitative data. The westerns are qualitative data that demonstrate that the proteins exist in the tissues. This is a binary (yes-no) determination, based on detectable bands, not relative abundance. GAPDH normalization and background correction for gels are required only for quantitative comparisons, when accuracy and precision in relative or absolute protein abundance are required. For example, such corrections would be necessary if the goal were to determine exact protein concentrations, calculate binding affinities, or perform quantitative comparisons of expression levels between conditions. There was no attempt at any of these types of measurements in our experiments. In this qualitative case (presence or absence), GAPDH serves to confirm protein loading in the lane and the test protein bands confirm the presence of the expressed protein. It cannot be argued that the proteins are absent. Thus, the quantitative assessment of mismatch repair protein (where expression level and age-related trends are addressed) were presented instead in the single-cell assays in Fig. 2, which were explicitly designed for that quantitative purpose.

(4) The biological replicates (n) we used in the congenic mouse studies are smaller than for more common condition of variable colony backgrounds but are grounded on well-established statistical principles. By definition, the number of biological replicates is determined by the variability in the experimental outcome. In this study, we-by design-generated a congenic mouse line, ensuring that all animals are genetically identical (clonal)—a process that required years of careful breeding. The great value of using a congenic strain lies in its near-zero inter-animal variability, which (by definition) dramatically reduces the number of animals needed to achieve statistical significance (below). Indeed, because the genetic background is identical in a congenic strain, gene expression profiles are effectively the same across animals. Statistical treatments for congenic strains are well developed, as described below (which has been added to the manuscript methods section).

The sample-size formula for congenic condition.

Cohen, J. (1988). *Statistical Power Analysis for the Behavioral Sciences* (2nd ed.). Hillsdale, NJ: Erlbaum.

For a simple two-group comparison, the biological sample size per group is:

$$n = \frac{2\sigma^2 \, (z_{1-\alpha/2} + z_{1-\beta})^2}{\delta^2}$$

Where:

- σ^2= biological variance
- δ= true mean difference (effect size)
- α= significance level

- β= Type II error (1 − power)
- z= score for the number of standard deviations from the mean that captures probability

In a congenic condition, the samples are genetically identical, and there is essentially no expected difference among protein expression among animals. Thus, δ=0.

$$n = \frac{2\sigma^2\,(z_{1-\alpha/2} + z_{1-\beta})^2}{0}$$
$$\Rightarrow n \rightarrow \infty$$

An infinite number of samples is needed to disprove the null hypothesis if the strain is congenic (i.e., the observed data are inconsistent with what the null hypothesis predicts (that there is no protein in the sample**).** Thus, it is the presence of band that provides direct evidence that the target protein Is present in the sample. For our western experiments, we only ask this yes-no qualitative question. Thus, two clonal congenic animals are sufficient to determine the answer. One congenic animal establishes the effect (protein is present), which is replicated in 1 or 2 additional animals. *When the true effect size is zero, increasing sample size only increases precision around a null effect and* any "significant" result in a technical replicate would be due to random sampling error. No significance is reported in the new plots.

In quantitative single cell experiments, where the biological sample is an animal tissue or cell, the statistical logic for congenic animals does not change**.** Given that a comparison is being made between genotypes in those experiments, however, it is desirable to increases precision around a null effect. Thus, in Figs. 5-8, we use minimally n = 3 (up to six in some cases) congenic mice to reduce the chance of sampling error and sample dozen-hundreds of individual points to determined significance of the means.

It is worth mentioning that the case for measuring protein expression is distinct from biological n that would be needed if behavior were being tested—even for congenic strains. That is because animal handling, testing geometry/equipment, room temperature and testing schedule can impose variability, and more animals would be needed for validating significance (i.e., δ is no longer 0). We have included a new section in the methods for this statistical discussion of congenic strains.

(5) Finally, a minor point. We are confused about the comment about the two blots in Fig 2. There are no blots in Fig. 2. All quantitative analysis begins in each figure with examples of the data, to show what kind of signal that is being measured. They are not the n used in the experiment. Shown in Fig. 2, for example, are examples of IF images of NeuN(+) or NeuN(-) cells, which co-stain with MSH3 or MSH6 (establishes cell type). The images were randomly selected among many tissue fields from the three WT or HD mice used in the analysis (another 16 WT and 16HD NeuN(+) examples are shown in Supple. Fig 2B). The MSH3 for MSH6 intensity from all cells (hundreds) in all animals are pooled according to genotype and plotted together per age group in the box plot, where each datapoint (a single cell) is shown in the plot. This allow a distribution and mean to be compared between the two groups.
